# Uncovering metabolic pathways relevant to phenotypic traits of microbial genomes

**DOI:** 10.1186/gb-2009-10-3-r28

**Published:** 2009-03-10

**Authors:** Gabi Kastenmüller, Maria Elisabeth Schenk, Johann Gasteiger, Hans-Werner Mewes

**Affiliations:** 1 Institute of Bioinformatics and Systems Biology, Helmholtz Zentrum München - German Research Center for Environmental Health, Ingolstädter Landstraße, D-85764 Neuherberg, Germany; 2 Computer-Chemie-Centrum, Universität Erlangen-Nürnberg, Nägelsbachstraße, D-91052 Erlangen, Germany; 3 Molecular Networks GmbH, Henkestraße 91, D-91052 Erlangen, Germany; 4 Chair for Genome-oriented Bioinformatics, Technische Universität München, Life and Food Science Center Weihenstephan, Am Forum 1, D-85354 Freising-Weihenstephan, Germany

## Abstract

Identifying the biochemical basis of microbial phenotypes is a main objective of comparative genomics. Here we present a novel method using multivariate machine learning techniques for comparing automatically derived metabolic reconstructions of sequenced genomes on a large scale. Applying our method to 266 genomes directly led to testable hypotheses such as the link between the potential of microorganisms to cause periodontal disease and their ability to degrade histidine, a link also supported by clinical studies.

## Abstract 2

A new machine learning-based method is presented here for the identification of metabolic pathways related to specific phenotypes in multiple microbial genomes.

## Background

Understanding complex phenotypic phenomena at the molecular level is a major goal in the post-genomic era. In particular, disease-related phenotypes of microorganisms are of interest, as a clear understanding of the underlying molecular processes can help to develop new drug/target combinations. Besides the phenotypes that directly cause particular diseases, another type of association, health-related phenotypes - where microorganisms living in a particular habitat (such as the human oral cavity or gut) affect human health - attracts more and more interest in this context [1-6].

In previous studies it has been shown that comparative genome analysis is well suited to assess interesting gene-phenotype associations for several phenotypic traits, such as hyperthermophily [7,8], flagellar motility [8-11], Gram-negativity [10-12], oxygen respiration [10,11], endospore formation [10,11], intracellularity [10] and for a variety of phenotypes extracted from the literature [13]. Except for the methods described by Slonim *et al*. [10] and Tamura and D'haeseleer [11], these methods do not provide any information on the biochemical context of the identified genes. Slonim *et al*. [10] clustered the genes associated with a phenotype and demonstrated that many of these clusters (gene modules) correspond to known metabolic or signaling pathways. Tamura and D'haeseleer [11] formed association networks of COGs (the National Center for Biotechnology Information's Clusters of Orthologous Groups of proteins [14]) based on multiple-to-one associations of COGs and phenotypes. These networks can be considered as functional modules.

In analogy to the concept of phylogenetic profiles introduced by Pellegrini *et al*. [15], the approaches mentioned above are based on the assumption that genomes that share a phenotypic property also share a set of orthologous genes. This implies that this method will miss associations with pathways if genes that catalyze the same sort of processes are not homologous, or if the loss of a relevant metabolic function results from the loss of different parts of a pathway. In these cases, no common aspects among phenotypically related species can be identified at the level of genes.

Recently, three systems have been described that provide both information on phenotypic properties of genomes and information on their metabolic pathways [16-18]. However, the Genome Properties system [16] and the PUMA2 system [17] list all pathways shared by the phenotypically related species rather than extracting only those pathways that are, in fact, associated with the phenotype. Therefore, the list contains many pathways that are not typical of the trait, but are, for example, very common in all genomes. Liu *et al*. [18] integrated clinical microbiological laboratory characterizations of bacterial phenotypes with various genomic databases, including the KEGG (Kyoto Encyclopedia of Genes and Genomes) pathway database [19]. The authors investigated univariate, pairwise associations of these phenotypes with KEGG pathways using the hypergeometric distribution. The approach thereby relies on the correlation of COGs [14] to phenotypes [20] and on the mapping of COGs to pathways. The COG database includes only manually annotated proteins, restricting the approach by Liu *et al*. to 59 prokaryotic organisms for which a time-consuming manual annotation has been achieved.

Our method goes beyond listing all pathways that are present in species showing a specific phenotype, as it uncovers pathway-phenotype associations. Based on the prediction and statistical analysis of metabolic pathways for 266 sequenced genomes, our method automatically finds pathways that are supposed to be relevant for a special phenotypic trait. Here, relevant means that the absence or presence or, more generally, the degree of completeness of these pathways in a genome is an important indicator for the trait. Moreover, our method shifts the univariate, pairwise association analysis to a multivariate analysis involving dependencies among pathways. In contrast to univariate statistics, multivariate statistical methods are able to identify pathways that are not individually associated with the phenotypic trait but become relevant in the context of other pathways. This allows for the identification of sets of pathways associated with a phenotype rather than individual pathway-phenotype associations. Finally, our method completely relies on annotation that has been automatically derived from genomic sequence data. Thus, it is not limited by the bottleneck of manual genome and protein annotation.

In general, shifting the focus of the analysis of phenotypes from genes to metabolic pathways (and thus assuming that genomes that share a phenotypic trait also share specific metabolic capabilities) not only facilitates functional interpretation of the results, but is also expected to be especially advantageous in cases of convergent evolution of taxonomically unrelated species towards a phenotype, since, for these species, sharing metabolic capabilities does not necessarily imply sharing orthologous genes.

We demonstrate here that our method is well suited to uncover the metabolic processes relevant for such phenotypic traits. Investigating periodontal disease [21] as a phenotype of the causative bacteria (which are taxonomically diverse), we also demonstrate that our method allows direct generation of hypotheses about the mechanism of the disease. These hypotheses are in good agreement with clinical studies and can give hints to new targets for the antibacterial treatment of periodontal disease. We also show that the identified relevant pathways can be used to classify genomes into traits with high selectivity. This classification goes beyond the assignment of functions to individual genes and the analysis of their phylogenetic profiles. Considering the growing number of sequencing projects on microorganisms and microbial ecosystems, the biochemical classification of genomes will become a valuable technique for the interpretation of genomic data.

## Results

In order to reveal a set of metabolic features typical of a phenotypic trait, we compared the completeness of metabolic pathways in genomes showing a particular phenotype and in genomes lacking it. For the comparison of metabolic pathways in different genomes, we had to consider that most known pathways (reference pathways) have been experimentally investigated only for a few model organisms. Many microbial organisms, pathogens in particular, are difficult to cultivate in the laboratory. Thus, a comparative method has to rely on metabolic reconstructions of completely sequenced genomes. Here, metabolic reconstruction means prediction of the metabolic complement of a genome in terms of reference pathways based exclusively on its genomic sequence information.

**Figure 1:**
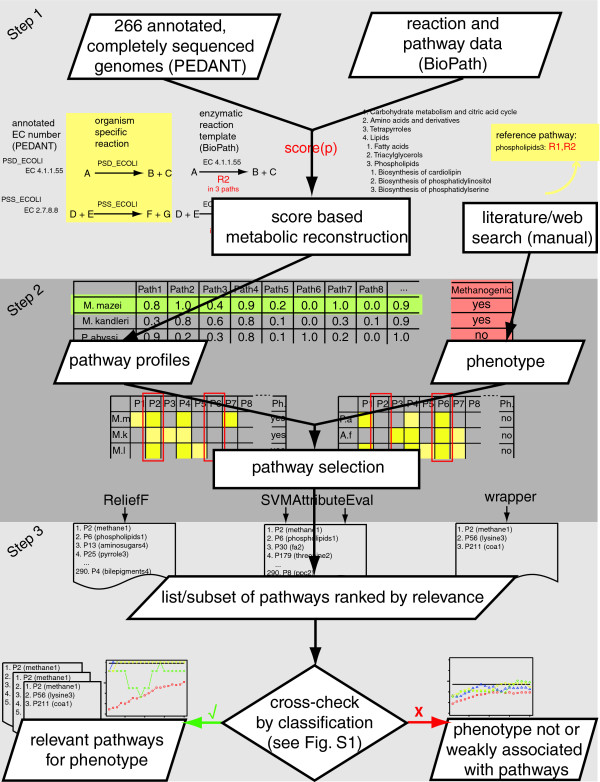
Overview of the approach. The three major steps of our approach are: metabolic reconstruction of completely sequenced genomes resulting in pathway profiles; pathway selection resulting in lists of pathways ranked by relevance; and cross-checking of the resulting pathway rankings by classification in order to estimate their significance (Figure S1 in Additional data file 2).

Assessing the metabolic complements of completely sequenced genomes, therefore, represents the first of the three major steps of our approach. For each phenotype under consideration, we then selected the subset of metabolic pathways that are most relevant in distinguishing the genomes showing the phenotype and the genomes lacking it. For this step we used (multivariate) statistical attribute selection methods. In a third step, we cross-checked the resulting sets of relevant pathways by classifying the genomes (into those showing a specific phenotype and those lacking it) based only on our predictions for the relevant pathways in the respective genomes. Figure 1 shows an overview of the method delineated in the following. A detailed description of each of its three steps is given in Materials and methods.

### Automatic metabolic reconstruction

In order to demonstrate the robustness of our machine learning approach, we based our analyses on a comparatively simple metabolic reconstruction procedure using automatic Enzyme Commission (EC) number [22] annotations. EC numbers for proteins and reactions are provided by most (automatic) annotation systems and most collections of reference pathways. Thus, the data basis used for our analyses can be considered as the least common denominator of such systems and collections.

In our studies, we compared the metabolic reconstructions of genomes on a large scale. In order to guarantee the comparability of the genomes' reconstructions, the EC number annotations on which the reconstructions are based had to be standardized, that is, derived by the same means for all genomes. (In cases of non-uniform annotations, we might select pathways that, for example, are more relevant in distinguishing annotation systems or authors than they are in distinguishing phenotypes.) The PEDANT system [23] provides standardized automatic genome and protein annotations for a large number of genomic sequences (see Materials and methods). For our analyses, we used all 266 completely sequenced genomes (28 eukaryotes, 23 archaea, 215 bacteria) that had been automatically annotated by PEDANT at the time of our study.

Based on the EC number assignments provided in PEDANT, we assessed the metabolic complement of each genome by scoring the completeness of each reference pathway (out of a set of reference pathways, which are defined by the EC numbers of the reactions involved) for the respective genomes. This reconstruction method is similar to the PathoLogic algorithm [24], which is used for the reconstructions in BioCyc [25]. In analogy to PathoLogic, our prediction procedure considers the ratio of enzymes in a pathway that are encoded in the genome and the uniqueness of these enzymes with respect to their occurrence in other pathways. (PathoLogic additionally uses the following criterion for pathway prediction: degradation and biosynthesis processes are considered as present only if the last two reaction steps or the first two reaction steps, respectively, are present.) In contrast to PathoLogic, our method results in a single score value for each reference pathway estimating the probability of the pathway to be present in a certain genome. Based on these pathway scores, the metabolic reconstruction of a genome can be represented by a numeric vector of scores in the form of a 'pathway profile'. On the one hand, this representation facilitates the comparison of metabolic capabilities by statistical methods. On the other hand, using the pathway score instead of a simple binary value (which can only indicate the presence or absence of a pathway in a genome) is advantageous for the analysis of parasitic genomes. Since these genomes often cover only parts of known reference pathways, a decision about presence or absence is often not appropriate. (Pathway profiles containing binary values or the ratios of available enzymes in pathways have been used in large scale analyses of metabolic complements, such as the evolutionary analyses by Liao *et al*. [26] and Hong *et al*. [27].)

Though our approach is not limited to a special pathway database, the choice of the underlying database is a critical point for any method that relies on pathway analysis. Green and Karp [28] showed that the outcome of any pathway analysis strongly depends on the conceptualization of the pathway database applied. Based on their studies, the authors recommended selecting the pathway database - and thus the conceptualization - that fits to the idea of the analysis planned. Our approach focuses on the comparative analysis of metabolic capabilities of organisms. For this type of analysis, the ability of an organism to degrade, for instance, L-histidine to L-glutamate, is of more interest than the specific enzyme variants used for this degradation. Thus, for our purposes, such enzyme variants should be included in the same reference pathway. In contrast, the degradation and the biosynthesis of L-histidine correspond to different metabolic capabilities and thus should be separated in distinct reference pathways. (Degradation (biosynthesis) processes that result in (start from) different products (educts) should also be separated in this context.)

KEGG [19] and MetaCyc [29] presumably are the most comprehensive sources for reference pathways available to date. KEGG provides a metabolite-centered, multi-organism view of metabolic pathways. This implies that a single KEGG reference pathway typically comprises several organism-specific enzyme variants in a single pathway. However, KEGG reference pathways as such are inapplicable for the kind of analysis considered in our approach, since they combine too many different biological processes, such as 'biosynthesis of L-histidine' and 'degradation of L-histidine', in a single reference pathway ('histidine metabolism'). MetaCyc pathways, on the other hand, represent distinct biological processes, but each pathway variant corresponds to a separate reference pathway. As an example, the degradation of L-histidine to L-glutamate is represented by three reference pathways in MetaCyc: 'histidine degradation I', 'histidine degradation II', and 'histidine degradation III'. These pathways overlap in three of four (or three of five in the case of histidine degradation II) reaction steps. Thus, by using MetaCyc, the focus of our analysis would slightly change to the identification of phenotype-related pathway variants.

For our studies, we chose BioPath [30], a free, publicly available electronic representation of the well known Roche Applied Science's Biochemical Pathways wall chart [31,32] as the source for reference pathways. BioPath reference pathways include alternative enzyme variants. Different biological processes, such as degradation and biosynthetic processes related to the same metabolite, are separated into distinct reference pathways. Hence, BioPath matches the pathway conceptualization required for our analysis. However, compared to MetaCyc, BioPath is less comprehensive with respect to the number of pathways and pathway variants.

### Pathway selection using machine learning

Applying our metabolic reconstruction method, the comparison of the metabolic capabilities of genomes is reduced to the comparison of their pathway profiles. However, due to the high number of genomes (266 in PEDANT) and reference pathways (290 in BioPath) it is almost impossible to sort out the pathways that are most relevant just by visual inspection of the profiles. Thus, we made use of machine learning methods in our approach. We applied statistical attribute selection in order to automatically extract the pathways (attributes) that are most relevant to a phenotype.

In general, attribute (here, pathway) selection results in a list of attributes (here, pathways) ranked by their significance for the distinction between instances (here, genomes represented by their pathway profiles) of class A (here, showing a specific phenotype) and class B (here, lacking this phenotype). If the investigated phenotype is caused by or otherwise related to special metabolic capabilities of genomes (and not only to regulatory or other effects), the top-ranking pathways are excellent indicators for functional peculiarities of the trait. Thus, these pathways can be used for both the functional classification of genomes and the interpretation of the biochemical basis of the phenotype.

Different attribute selection methods focus on different aspects of the data analyzed [33]. In order to get a reliable and (biologically) comprehensive collection of phenotype-associated pathways, we applied three (multivariate) attribute selection methods with different characteristics and joined their results: the filter method ReliefF [34-36], the embedded method SVMAttributeEval [37], and a wrapper method using a naïve Bayes classifier [38]. In general, filters remove irrelevant attributes based on the intrinsic characteristics of the data (that is, they remove attributes with low relevance weights according to univariate (for example, gain ratio, chi square) or multivariate (for example, ReliefF) criteria). Wrappers, on the other hand, evaluate attributes by using accuracy estimates provided by a certain classification algorithm. Embedded methods are also specific to a given learning machine. But these methods select attribute subsets during the training of the learning machine. ReliefF does not remove statistically dependent attributes. As we are interested in all relevant pathways rather than in the smallest subset of pathways providing the highest classification accuracy, this makes ReliefF well suited for our purposes. In contrast naïve Bayes is very sensitive to dependent attributes. Therefore, a wrapper using naïve Bayes is expected to omit these attributes. Thus, it should complement the results of ReliefF. (For more details see Materials and methods.)

### Cross-check of relevant pathways by classification

In order to estimate the significance of the pathway rankings resulting from pathway selection for a phenotype, we cross-checked the rankings by classifying the genomes (into those showing the phenotype and those lacking it) based only on the pathway scores for the selected pathways. In order to do so, we represented the genomes by pathway profiles that have been reduced to the best ranking 1, 2, 3, ..., 20 pathways. These reduced pathway profiles (that is, vectors with 1, 2, 3, ..., 20 dimensions) and the phenotypic information on the genomes have been used as input for four different classification algorithms (J48, IB1, naïve Bayes, and SMO). After cross-validation, we compared the achieved classification quality of the resulting classifiers to the quality reached by classification based on all pathways (that is, complete pathway profiles) and based on randomly chosen 1, 2, 3, ..., 20 pathways (average quality of 25 times). In order to assess the quality of classification, we calculated the product of classification selectivity and sensitivity. In addition, we determined the receiver operating characteristic (ROC) area under the curve (AUC) value; for details see Materials and methods.

**Figure 2:**
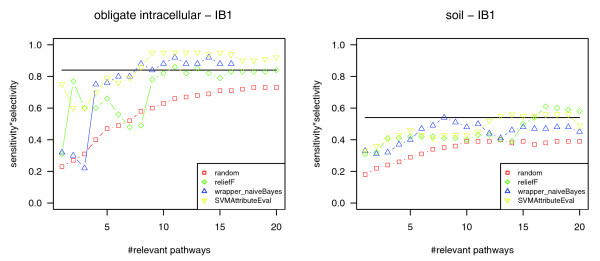
Estimating the significance of pathway rankings provided by pathway selection. For phenotypes that are weakly associated with the presence or absence of specific metabolic pathways, the classification quality should be within the same range for classification based on randomly picked pathways (red), all pathways (marked by a horizontal line), and pathways highly ranked in attribute subset selection (green, ReliefF; yellow, SVMAttributeEval; blue, wrapper (naïve Bayes)). As an example, the right diagram shows the classification quality for the phenotype 'habitat: soil' (depending on the number of top-ranking pathways used for classification). In this case, the top-ranking pathways provided by attribute subset selection are considered as not significant for the phenotype. The left diagram shows the classification quality values for the phenotype 'obligate intracellular'. Using the most relevant pathways for classification results in higher classification quality compared to using all pathways or randomly picked pathways. Furthermore, the quality values lie above 0.6. In this case, the most relevant pathways derived by attribute subset selection are considered as significant.

Phenotypes that are not or only weakly associated with specific metabolic capabilities might, nonetheless, be developed by species that are similar in their complete metabolism. In this case any set of randomly picked pathways might have nearly the same (high) predictive power as the selected ones. Similarly, if a phenotype is due to any effect that is not covered by our method (for example, if there are many completely different metabolic patterns that lead to the same phenotype or if the phenotype is related to regulatory effects), we expect that the (in this case low) classification quality lies within the same range for classification based on randomly picked pathways, all pathways, and pathways highly ranked in pathway selection. We are not able to associate (significantly) relevant pathways with any of these types of phenotypes. The results for the phenotype 'habitat: soil' using the classifier IB1 are shown in Figure 2 (right) as an example of such cases. As a consequence, we considered the high-ranking pathways as relevant for the phenotype only if the following applied to at least one of the four classifications: the quality of classification based on the top-ranking pathways (i) was considerably better than random, (ii) at least reached the classification quality achieved for all pathways, and (iii) at least reached a value of 0.6. As an example, Figure 2 (left) shows the resulting classification quality values depending on the number of considered pathways for the phenotype 'obligate intracellular' using the nearest neighbor classifier (IB1).

### Metabolic analysis of phenotypic traits

For our analyses, we used all 266 completely sequenced genomes (28 eukaryotes, 23 archaea, 215 bacteria) that had been automatically annotated by PEDANT at the time of our study (see Materials and methods). For each genome, we collected information about presence or absence of different phenotypic traits related to Gram stain, oxygen usage, habitat (soil, oral cavity), relation to diseases, and intracellularity. (For the complete list of genomes and phenotypes see Additional data file 1.) To infer the metabolic complements of these genomes, we applied our metabolic reconstruction method to each genome using the automatic genome annotation provided by PEDANT and the (organism unspecific) metabolic reaction and pathway data given by BioPath (for details see Materials and methods). The reconstruction results in a 290-dimensional pathway profile for each genome. Each dimension corresponds to the weighted completeness of a reference pathway described by a pathway reconstruction score. This score is normalized to values ranging from 0 (no reaction of the pathway is catalyzed) to 1 (pathway is complete).

For each phenotype, we applied the attribute subset selection methods ReliefF, SVMAttributeEval, and wrapper (naïve Bayes) to the pathway profiles of the complete set of genomes. After cross-validation we received a list of pathways (attributes) ranked by the relevance of the pathway for each selection method. Whereas ReliefF and SVMAttributeEval provide a complete ranking of all pathways, the wrapper yields partially ranked subsets of pathways. The results of each attribute selection were cross-checked by classification using IB1, J48, naïve Bayes, and SMO, respectively.

In the following, we first show the applicability of our method for a relatively simple example, the phenotype 'methanogenesis'. This rare phenotype is mainly defined by the common pathway of methanogenesis from H_2 _and CO_2_. Thus, we expected that our method would determine this pathway to be the most relevant pathway. Then, we present our results for a more sophisticated example, the phenotype 'periodontal disease causing'. The results for the phenotypes 'Gram-positive', 'obligate anaerobe', 'obligate intracellular', and 'habitat: soil' are available in Additional data file 2.

### Methanogenesis

Methanogens are strictly anaerobic archaea producing methane as a major product of their energy metabolism [39]. Apart from methanogenesis, they are quite diverse in their metabolic capabilities. Only six completely sequenced genomes showing this phenotype are available within PEDANT (*Methanococcus jannaschii*, *Methanococcus maripaludis*, *Methanopyrus kandleri AV19*, *Methanosarcina acetivorans C2A*, *Methanosarcina mazei Goe1*, *Methanothermobacter thermoautotrophicus*). Nonetheless, they cover all four phylogenetically different classes of methanogens: Methanobacteria, Methanococci, Methanomicrobia, Methanopyri.

**Figure 3:**
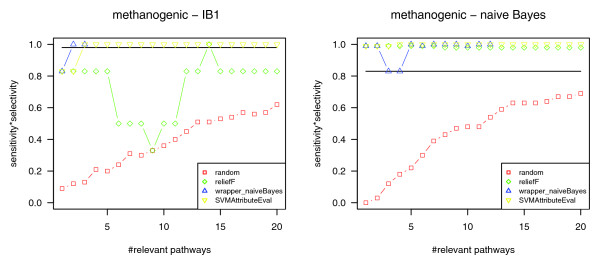
Cross-checking for the phenotype methanogenesis. The classification quality diagrams for nearest neighbor classifier (IB1) and the naïve Bayes classifier show that the identified most relevant pathways are well suited to distinguish methanogens and non-methanogens (sensitivity × selectivity = 1.0). According to the cross-check, the most relevant pathways identified by pathway selection are considered as significant. Apart from using ReliefF top-ranking pathways (green) for the classification with IB1, the maximum classification quality is already reached for the (up to) five most relevant pathways (these pathways are listed in Table 1).

As expected, pathway selection and the following cross-check for the complete dataset (266 genomes) of pathway profiles confirmed that methanogenesis is reflected at the level of metabolism. Figure 3 shows the resulting classification quality values for the nearest neighbor classifier IB1 and the naïve Bayes classifier depending on the number of (most relevant) pathways (1-20) that have been considered for classification (the corresponding classification quality diagrams for the classifiers J48 and SMO are available in Additional data file 2). According to the cross-check, the phenotype 'methanogenesis' is significantly associated with the identified relevant pathways. As one can see from the classification quality diagrams, for any combination of attribute selection method (ReliefF, SVMAttributeEval, wrapper (naïve Bayes)) and classifier except the combination ReliefF/IB1, the maximum classification quality is already reached using the (up to) five most relevant pathways (for the respective pathways, see Table 1). Therefore, we focus on these pathways in the following.

**Table 1:** Relevant pathways for methanogenesis

Dataset	ReliefF	SVMAttributeEval	Wrapper (naïve Bayes)
Complete (266)	Reduction of CO_2 _to CH_4 _(methane1) ↑	Reduction of CO_2 _to CH_4 _(methane1) ↑	Reduction of CO_2 _to CH_4 _(methane1) ↑
	Biosynthesis of cardiolipin (phospholipids1) ↓	Biosynthesis of cardiolipin (phospholipids1) ↓	Degradation of L-lysine to crotonyl-CoA (lysine3) ↓
	Biosynthesis of peptidoglycan I (aminosugars4) ↓	beta-Oxidation of fatty acids (fa2) ↓	Biosynthesis of coenzyme A (coa1) ↓
	Heme biosynthesis (pyrrole3) ↓	Degradation of L-threonine to L-2-aminoacetate (threonine2) ↓	
	Pentose phosphate cycle (non-oxidative branch) (ppc3) ↓	Biosynthesis of phosphatidylserine (phospholipids3) ↑	

Archaea (23)	Biosynthesis of 2'-deoxythymidine-5'-triphosphate (dtn1) ↑	Biosynthesis of 2'-deoxythymidine-5'-triphosphate (dtn1) ↑	Reduction of CO_2 _to CH_4 _(methane1) ↓
	Reduction of CO_2 _to CH_4 _(methane1) ↑	Biosynthesis of L-phenylalanine from chorismate (aaa4) ↑	Biosynthesis of 2'-deoxythymidine-5'-triphosphate (dtn1) ↑
	Biosynthesis of phosphatidylserine (phospholipids3) ↑	Reduction of CO_2 _to CH_4 _(methane1) ↑	Degradation of L-threonine to L-2-aminoacetate (threonine2) ↓
	Degradation of L-threonine to L-2-aminoacetate (threonine2) ↓	Degradation of dGMP to deoxyguanosine (dgn2) ↓	Degradation of L-lysine to crotonyl-CoA (lysine3) ↓
	Degradation of tryptophane to 6-hydroxymelatonin (trp5) ↑	Biosynthesis of phosphatidylserine (phospholipids3) ↑	Biosynthesis of coenzyme B12 (coba1) ↑

Archaea (23) (without methane1)	Biosynthesis of 2'-deoxythymidine-5'-triphosphate (dtn1) ↑	Biosynthesis of 2'-deoxythymidine-5'-triphosphate (dtn1) ↑	Biosynthesis of 2'-deoxythymidine-5'-triphosphate (dtn1) ↑
	Biosynthesis of phosphatidylserine (phospholipids3) ↑	Biosynthesis of L-phenylalanine from chorismate (aaa4) ↑	Biosynthesis of coenzyme B12 (coba1) ↑
	Degradation of L-threonine to L-2-aminoacetate (threonine2) ↓	Degradation of L-threonine to L-2-aminoacetate (threonine2) ↓	Degradation of L-valine (vas4) ↓
	Degradation of tryptophane to 6-hydroxymelatonin (trp5) ↑	Biosynthesis of phosphatidylserine (phospholipids3) ↑	Degradation of L-threonine to L-2-aminoacetate (threonine2) ↓
	Biosynthesis of coenzyme B12 (coba1) ↑	Odd-numbered fatty acid metabolism (glf2) ↓	Degradation of L-lysine to crotonyl-CoA (lysine3) ↓

The relevant pathways for methanogenesis were determined by applying three different attribute selection methods (ReliefF, SVMAttributeEval, and a wrapper for the naïve Bayes classifier) to three datasets. The (up to) five most relevant pathways received for the complete set of pathway profiles (266 genomes), the archaeal pathway profiles (23 genomes), and the archaea profiles (23 genomes) without the attribute 'methane1' are shown. An upwards pointing arrow denotes pathways that are relevant due to higher pathway scores (that is, pathways are more complete) in methanogens compared to the other genomes in the investigated dataset. In analogy, a downwards pointing arrow denotes pathways that are relevant due to lower pathway scores (that is, pathways are less complete) in methanogens.

As expected, our method found the pathway of methane synthesis from H_2 _and CO_2 _(methane1) to be the most relevant pathway for the phenotype 'methanogenesis'. In addition, we found the following pathways to be relevant by showing either specifically higher or lower pathway scores for genomes showing the phenotype (Table 1): biosynthesis of phosphatidylserine (phospholipids3) (higher); biosynthesis of cardiolipin (phospholipids1) (lower); biosynthesis of peptidoglycan (part I) (aminosugars4) (lower); beta-oxidation of fatty acids (fa2) (lower); pentose phosphate cycle (non-oxidative branch) (ppc3) (lower); heme biosynthesis (pyrrole3) (lower); degradation of L-lysine to crotonyl-CoA (lysine3) (lower); degradation of L-threonine to L-2-aminoacetate (threonine2) (lower); and biosynthesis of coenzyme A (coa1) (lower).

#### Biosynthesis of phosphatidylserine and cardiolipin

Phosphatidylserine and cardiolipin are both components of biological membranes. Differences in membrane lipids led to the distinction of the domain of archaea from the domain of bacteria [40]. Furthermore, composition and biosynthetic pathways of polar lipids in methanogens differ from those of other groups of archaea [41,42]. Among the archaea, phospholipids with amino groups, such as phosphatidylserine, only occur in methanogens and some related Euryarchaeota. This is reflected by the pathway score. For all six methanogens in our dataset as well as for five other archaea (*Haloarcula marismortui ATCC43049*, *Halobacterium salinarum NRC1*, *Archaeoglobus fulgidus*, *Thermoplasma acidophilum*, *Natronomonas pharaonis DSM 2160*), the pathway score is ≥ 0.75, whereas it is ≤ 0.25 for all other archaea in the dataset. For phosphatidylserine, Morii and Koga [42] suggested a pathway consisting of five steps (starting from glyceraldehyde-3-P) analogous to the pathway in bacteria. The phosphatidylserine synthase, which catalyzes the last step of this pathway in methanogens and some related Euryarchaeota, is similar to the corresponding enzyme in Gram-positive bacteria. Thus, the authors speculated that the ancestral encoding gene was transferred from a Gram-positive bacterium. This is in good agreement with our results, as our method found the pathway of biosynthesis of phosphatidylserine to be relevant also in distinguishing Gram-positive and Gram-negative bacteria (Additional data file 2). In contrast to the biosynthesis of phosphatidylserine, the synthesis of cardiolipin is not operative in most archaea in the dataset (except *Halobacterium salinarum NRC1*) according to our predictions. Cardiolipin is related to oxidative processes and is known to be synthesized by *Halobacterium salinarum *[43].

#### Biosynthesis of peptidoglycan (part I: biosynthesis of N-acetylmuramic acid)

Peptidoglycan (murein) is a cell wall polymer common to most eubacteria [31]. In the first phase of its biosynthesis N-acetylmuramate is formed. Members of the domain archaea lack peptidoglycan in their cell wall. Some archaea have developed a polymer called pseudopeptidoglycan (pseudomurein), which is functionally and structurally similar, but chemically different from eubacterial murein. Instead of N-acetylmuramic acid, pseudomurein contains N-acetyltalosaminuronic acid (the biosynthetic pathway of N-acetyltalosaminuronic acid is not included in BioPath). The relevance of the N-acetylmuramic acid pathway in distinguishing methanogens from non-methanogens presumably represents the differences in cell wall composition of archaea compared to eubacteria and identifies methanogens as archaebacteria.

#### Biosynthesis of coenzyme A

Coenzyme A is an acyl group carrier and plays a central role in cellular metabolism. In BioPath, the biosynthetic pathway 'biosynthesis of coenzyme A' (coa1) includes both the biosynthesis of coenzyme A from pantothenate and the *de novo *synthesis of pantothenate. In several non-methanogenic archaea, the set of enzymes for the synthesis of pantothenate is conserved with the corresponding bacterial or eukaryotic enzymes. In methanogenic archaea, however, neither homology nor non-homology based methods could identify enzymes for the synthesis of pantothenate. Thus, autotrophic methanogens follow a unique pathway for *de novo *biosynthesis of coenzyme A [44].

#### Pentose phosphate cycle (non-oxidative branch)

In the non-oxidative branch of the pentose phosphate cycle, various sugars with three, four, five, six, or seven carbon atoms are interconverted to each other. But genes for this pathway are missing in most archaeal genomes [45]. Analogous to the peptidoglycan pathway, the occurrence of this pentose phosphate cycle branch indicates that all methanogens show properties of archaea.

#### Heme biosynthesis (part II)

Heme is the prosthetic group of many important heme proteins, which are involved in electron transfer or gas transport. Heme proteins such as cytochromes a, b, and c and catalase are also known for archaea. For the first part of heme synthesis from delta-aminolevulinic acid to uroporphyrinogen III, the homologs of the corresponding eukaryotic and bacterial enzymes are present in many archaea. But for the conversion of uroporphyrinogen III to protoheme, most archaea (except *Thermoplasma volcanium*) lack homologs [46]. The relevance of this pathway for the phenotype 'methanogenesis' presumably arises from the fact that all methanogens known so far are members of the archaea domain.

#### (Aerobic) beta-oxidation of fatty acids

This pathway depends on aerobic conditions and is missing in the six methanogens contained in PEDANT. Thus, its occurrence in the list of relevant pathways may refer to the anaerobic lifestyle of methanogens. Our results for distinguishing obligate anaerobes and obligate aerobes also support this assumption, as the pathway of beta-oxidation of fatty acids is one of the five most relevant pathways for this phenotype (Additional data file 2).

#### Degradation of L-threonine to L-2-amino-acetoacetate and degradation of L-lysine to crotonyl-CoA

In general, degradation of amino acids can be used either to gain energy or to generate fatty acids [47]. Both degradation pathways, which our method identified as relevant, are not operative in methanogens according to our metabolic reconstructions. In some anaerobic microorganisms, degradation of several amino acids is coupled to methanogens by a syntrophic relationship: hydrogen, which is produced by the oxidation of the amino acid in the degrading organism, is consumed in methanogenesis by the methanogenic organism [48]. Thus, looking at these degradation processes presumably helps to distinguish methanogens from other anaerobic genomes.

### Methanogens among archaea

**Figure 4:**
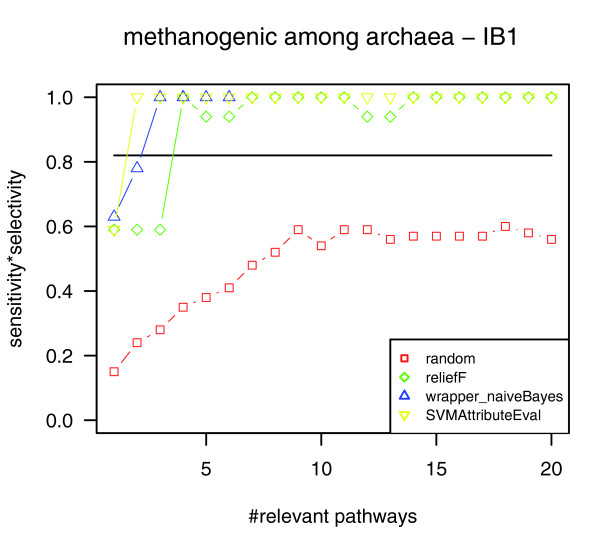
Classification quality for the classification of archaea into methanogens and non-methanogens using the nearest neighbor classifier. The classification based on the four most relevant pathways yields a perfect separation of methanogenic archaea and non-methanogenic archaea for all attribute subset selection methods used (green, ReliefF; yellow, SVMAttributeEval; blue, wrapper (naïve Bayes)). Classification based on all pathways (marked by a horizontal line) and based on randomly picked pathways (red) show lower classification quality.

In order to determine pathways that reflect methanogenic rather than archaeal properties, we also applied our method to the subset of archaeal genomes (23 pathway profiles). The classification of archaea into methanogens and non-methanogens based on the newly derived five most relevant pathways yielded a classification quality above 0.8 for all attribute selection methods and all classifiers except J48 for the five most relevant pathways determined by the wrapper (0.59; Table 2 and Figure 4). The resulting rankings of relevant pathways still contained methane1, phospholipids3, threonine2, and lysine3 within the top five positions. Additionally, the pathway of 'biosynthesis of 2'-deoxythymidine-5'-triphosphate' (dtn1) ranked among the five most relevant pathways for each attribute selection method applied. (For further pathways that rose in rank for only one of the attribute selection methods, see Table 1.) In contrast to the results for all genomes, pathways related only to archaeal or anaerobic properties (ppc3, pyrrole3, aminosugars4) did not occur among the five most relevant pathways any more.

**Table 2:** Classification quality for the classification of 23 archaeal genomes into methanogens and non-methanogens using the 5 most relevant pathways

Classifier	ReliefF	SVM	Wrapper	All pathways	Random
J48	0.88	0.88	0.59	0.83	0.17
IB1	0.94	1.00	1.00	0.29	0.31
Naïve Bayes	0.94	1.00	0.83	0.83	0.38
SMO	1.00	1.00	1.00	1.00	0.01

The 23 archaeal genomes were classified into methanogens and non-methanogens using only the five most relevant pathways from Table 1. We applied four different classifiers (J48, IB1, naïve Bayes, and SMO) with tenfold cross-validation. In addition, the genomes were classified based on all pathways (290) in the pathway profile as well as on five randomly chosen pathways. To estimate the quality of classification, we calculated the product of classification selectivity and sensitivity, which is shown in this table. In the case of randomly chosen pathways, the value was derived by averaging the classification quality of 25 sets of 5 randomly chosen pathways.

For the synthesis of thymidylate (2'-deoxythymidine-5'-monophosphate), which is the first step of dtn1, two alternative mechanisms are known so far. In these two mechanisms the synthesis is catalyzed by ThyA (2.1.1.45) and ThyX (2.1.1.148), respectively. Both, ThyA and ThyX show a broad phylogenetic distribution, but usually only one or the other is encoded by a genome [49,50]. In BioPath, the reference pathway for 'biosynthesis of 2'-deoxythymidine-5'-triphosphate' (dtn1) only contains the more classic route via ThyA. Using our reconstruction method, we predicted that all methanogens contained in our data follow this classic route, whereas most other archaea (except *Archaeoglobus fulgidus *and *Natronomonas pharaonis*) lack this pathway. Thus, in this case, the identified difference between methanogens and archaea is presumably due to differences in pathway variants rather than differences in the presence or absence of the respective metabolic capability.

### Methanogens among archaea disregarding methane1

**Figure 5:**
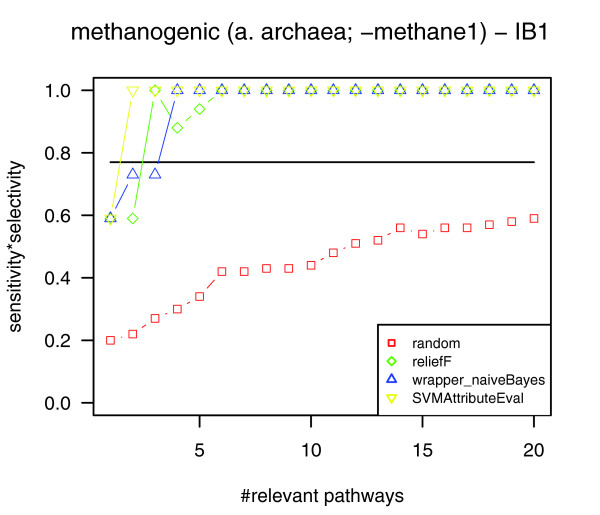
Classification quality for the classification of archaea into methanogens and non-methanogens using the nearest neighbor classifier while omitting the pathway of methane synthesis. Omitting the pathway of methane synthesis (methane1) in our analyses, the classification based on the most relevant pathways still reaches perfect separation of methanogenic archaea and non-methanogenic archaea for all attribute subset selection methods used (green, ReliefF; yellow, SVMAttributeEval; blue, wrapper (naïve Bayes)). Classification based on all pathways (marked by a horizontal line) and based on randomly picked pathways (red) show lower classification quality.

In order to ensure that the good classification quality was not mainly due to the high relevance of methane1, we deleted methane1 from the pathway profiles and repeated our analysis. Thereby, we received almost the same set of relevant pathways (Table 1) and an almost as high classification quality as with methane1 (Table 3 and Figure 5).

**Table 3:** Classification quality for the classification of 23 archaeal genomes into methanogens and non-methanogens using the 5 most relevant pathways derived from pathway profiles without methane1

Classifier	ReliefF	SVM	Wrapper	All pathways except methane1	Random
J48	0.59	0.88	0.59	0.67	0.01
IB1	0.94	1.00	1.00	0.29	0.40
Naïve Bayes	0.78	1.00	0.77	0.67	0.59
SMO	1.00	1.00	1.00	1.00	0.00

The 23 archaeal genomes were classified into methanogens and non-methanogens using only the five most relevant pathways from Table 1. These relevant pathways were derived by attribute subset selection based on pathway profiles without the pathway methane1. We applied four different classifiers (J48, IB1, naïve Bayes, and SMO) with tenfold cross-validation. In addition, the genomes were classified based on all pathways (290) in the pathway profile as well as on five randomly chosen pathways. To estimate the quality of classification, we calculated the product of classification selectivity and sensitivity, which is shown in this table. In the case of randomly chosen pathways, the value was derived by averaging the classification quality of 25 sets of 5 randomly chosen pathways.

### Causing periodontal disease

Periodontal disease is a bacterial infection of the tissues surrounding and supporting the teeth. Symptoms vary from inflammation and bleeding of the gums to teeth loss due to destruction of the bone around the teeth. In many studies, periodontal disease was related to an increased amount of *Fusobacterium nucleatum*, *Porphyromonas gingivalis*, *Treponema denticola*, *Tannerella forsythia*, *Prevotella intermedia*, and *Aggregatibacter actinomycetemcomitans *in the oral flora of patients compared to healthy controls [51-54].

The human oral flora consists of more than 700 species [55], of which less than half can be grown in the laboratory. At the time of our study, PEDANT contained 15 fully sequenced oral genomes (as annotated by NCBI and Karyn's genomes) including four (*F. nucleatum ATCC25586*, *P. gingivalis W83*, *T. denticola ATCC35405*, and *A. actinomycetemcomitans *(serotype b) *HK1651*) of the six periodontal pathogens.

**Figure 6:**
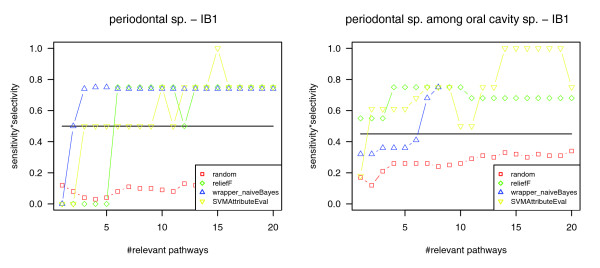
Classification quality for the phenotype periodontal disease causing. Left: classification of all genomes (266) into genomes related and not related to periodontal disease using the nearest neighbor classifier (IB1). Right: classification of oral genomes (15) into genomes related and not related to periodontal disease using the nearest neighbor classifier (IB1). Compared to classification based on all pathways (marked by a horizontal line) and based on randomly picked pathways (red), the classification based on the most relevant pathways yields better separation of periodontal species and other species in both genome datasets.

Analogous to the previous example of methanogenesis, we applied our method to the complete set of pathway profiles (266 species) as well as to the reduced set of 15 oral genomes to focus on periodontal-related rather than oral cavity-related biochemical features. Figure 6 shows the resulting classification qualities achieved with the nearest neighbor classifier. According to the cross-check, the phenotype 'periodontal disease causing' is reflected by the identified relevant pathways. In contrast to the phenotype 'methanogenesis', more highly ranking pathways must be considered for classification to reach the maximum classification quality. Therefore, we focus on the ten most relevant pathways in the following. Using these pathways, we obtained 0.75 as the maximum classification quality value in both genome sets compared to a maximum of 0.50 for all pathways and maximums of 0.08 and 0.29, respectively, for randomly chosen pathways (Table 4).

**Table 4:** Classification quality for the classification of genomes into genomes related and unrelated to periodontal disease using the corresponding 10 most relevant pathways

Classifier	ReliefF	SVM	Wrapper	All pathways	Random
J48	0.50	0.00	0.00	0.00	0.01
	(0.68)	(0.68)	(0.36)	(0.25)	(0.29)
					
IB1	0.75	0.75	0.74	0.50	0.08
	(0.75)	(0.50)	(0.75)	(0.18)	(0.28)
					
Naïve Bayes	0.65	0.50	0.72	0.50	0.05
	(0.61)	(0.75)	(0.75)	(0.45)	(0.29)
					
SMO	0.00	0.75	0.00	0.25	0.00
	(0.75)	(0.68)	(0.36)	(0.50)	(0.11)

The 266 (15 oral cavity) genomes of the complete data set were classified into genomes related and not related to periodontal disease using only ten (eight in the case of wrapper) most relevant pathways derived by the three attribute selection methods. We applied four different classifiers (J48, IB1, naïve Bayes, and SMO) with tenfold cross-validation. In addition, the genomes were classified based on all pathways (290) in the pathway profile as well as on ten randomly chosen pathways. To estimate the quality of classification, we calculated the product of classification selectivity and sensitivity, which is shown in this table. In the case of randomly chosen pathways, the value was derived by averaging the classification quality of 25 sets of 10 randomly chosen pathways. The data in parentheses are for the dataset containing the 15 oral cavity genomes.

**Figure 7:**
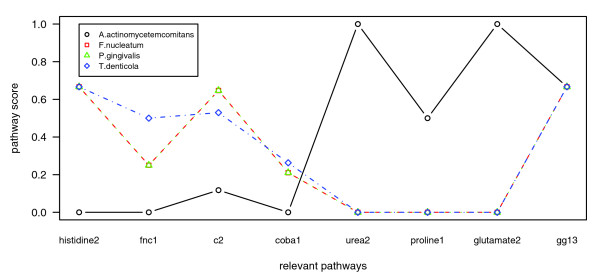
Pathway scores of the relevant pathways for the periodontal species. Plotting the pathway scores of the relevant pathways (from Table 6), the differences of *A. actinomycetemcomitans *(black) compared to *F. nucleatum *(red), *P. gingivalis *(green), and *T. denticola *(blue) become apparent. In contrast, the scores for *F. nucleatum*, *P. gingivalis*, and *T. denticola *are very similar.

The classification quality did not reach 1.00 for any combination of attribute selection method and classifier because *A. actinomycetemcomitans *was always misclassified. Plotting the pathway scores of the relevant pathways, the differences of *A. actinomycetemcomitans *compared to *F. nucleatum*, *P. gingivalis*, and *T. denticola *become apparent (Figure 7). In contrast, the scores for *F. nucleatum*, *P. gingivalis*, and *T. denticola *are very similar. This 'outlier' role of *A. actinomycetemcomitans *agrees with studies of Socransky *et al*. [52]. They investigated the co-occurrence of bacterial species in a large number of subgingival plaque samples (collected from hundreds of patients) and identified five major clusters of bacteria, which they designated by the colors red, orange, green, yellow, and purple. *A. actinomycetemcomitans *(serotype b) did not fall in one of these clusters. The cluster holding *P. gingivalis*, *T. denticola*, and *T. forsythensis *(called the 'red' cluster in [52]) and the cluster consisting of *F. nucleatum *and some Prevotella and not yet sequenced Centruroides species (called the 'orange' cluster in [52]), were associated with clinical measures of periodontal disease. *A. actinomycetemcomitans*, however, was not found to be significantly enhanced for periodontal disease in Socransky *et al*. [52]. Nonetheless, according to several studies, a high-toxic JP2 clone of *A. actinomycetemcomitans *(serotype b) (strain HK1651 is a representative of this clone) is strongly associated with localized juvenile periodontitis in adolescents of African origin [53]. Based on the major differences of *A. actinomycetemcomitans *compared to the other pathogens in our analyses, one could speculate that the mechanisms causing the disease might also differ.

**Figure 8:**
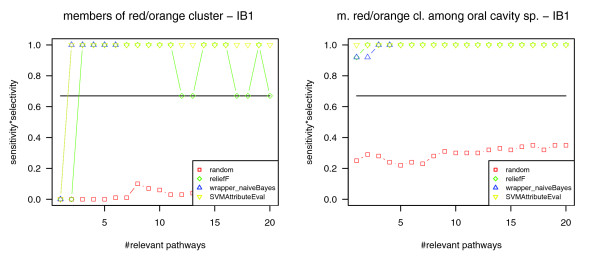
Classification quality for the phenotype member of red or orange cluster. Left: classification of all genomes (266) into genomes that are members and non-members of the 'red/orange' cluster using the nearest neighbor classifier (IB1). Right: classification of oral genomes (15) into genomes that are members and non-members of the 'red/orange' cluster related using the nearest neighbor classifier (IB1). Compared to classification based on all pathways (marked by a horizontal line) and based on randomly picked pathways (red), the classification based on the most relevant pathways yields better separation of the cluster members and other species in both genome datasets.

In order to get more specific insights for the three species of the 'red' and 'orange' clusters, we repeated the procedure described above for the phenotype 'member of the red or orange cluster'. (Since Socransky *et al*. [52] derived those clusters based on clinical measures for the co-occurrence of oral species, this phenotype can be considered as a clinical phenotype.) As expected, we received enhanced classification quality (Table 5 and Figure 8). The pathways that are among the ten most relevant pathways for at least one attribute selection method and for at least three of the four investigated datasets are listed in Table 6 and briefly described below (for all pathways, see Additional data file 2). In Table 6, these datasets are abbreviated by two characters. The first character denotes the phenotypic information used: 3 ='members of red or orange cluster' and 4 ='periodontal disease causing'. The second character denotes the set of genomes in the dataset: A = all genomes (266); O = oral cavity genomes (15). (This results in the following abbreviations for the four combinations of phenotypic information and genome sets that have been investigated: 4A, 'periodontal disease causing' genomes in the complete dataset (266 genomes); 4O, 'periodontal disease causing' genomes in the oral cavity dataset (15 genomes); 3A, 'members of red or orange cluster' in the complete dataset; 3O, 'members of red or orange cluster' in the oral cavity dataset.)

**Table 5:** Classification quality for the classification of genomes into members and non-members of the red or orange cluster by using the corresponding 10 most relevant pathways

Classifier	ReliefF	SVM	Wrapper	All pathways	Random
J48	0.67	0.00	0.67	0.00	0.00
	(0.92)	(0.92)	(0.92)	(0.00)	(0.20)
					
IB1	1.00	1.00	1.00	0.67	0.08
	(1.00)	(1.00)	(1.00)	(0.61)	(0.16)
					
Naïve Bayes	1.00	0.67	1.00	0.67	0.12
	(1.00)	(1.00)	(0.67)	(0.67)	(0.14)
					
SMO	1.00	1.00	0.67	0.00	0.00
	(1.00)	(1.00)	(0.92)	(0.67)	(0.08)

The 266 (15 oral cavity) genomes of the complete data set were classified into members and non-members of the red or orange cluster using only the ten (in the case of wrapper, 6 (266 genomes) or 4 (15 genomes)) most relevant pathways (Additional data file 2) derived by the three attribute selection methods, respectively. We applied four different classifiers (J48, IB1, naïve Bayes, and SMO) with tenfold cross-validation. In addition, the genomes were classified based on all pathways (290) in the pathway profile as well as on ten randomly chosen pathways. To estimate the quality of classification, we calculated the product of classification selectivity and sensitivity, which is shown in this table. In the case of randomly chosen pathways, the value was derived by averaging the classification quality of 25 sets of 10 randomly chosen pathways. The data in parentheses are for the dataset containing the 15 oral cavity genomes.

**Table 6:** Relevant pathways for the phenotype 'periodontal disease causing'

Relevant pathway	Dataset	Attribute selection method
Biosynthesis of coenzyme B12 (coba1) ↑	4A, 4O, 3A, 3O	R, S, W
Biosynthesis of L-proline (proline1) ↓	4A, 4O, 3A, 3O	R, S, W
Glutamate fermentation (fnc1) ↑	4A, 4O, 3A, 3O	R, S, W
Biosynthesis of 5-formimino-THF (c2) ↑	4A, 4O, 3A, 3O	R, S
Urea cycle (part) (urea2) ↓	4A, 4O, 3A, 3O	R, S
Conversion of L-glutamate to L-proline (glutamate3) ↓	4A, 4O, 3A, 3O	R, S
Conversion of L-glutamate to L-ornithine (glutamate2) ↓	4A, 4O, 3A, 3O	R
Degradation of L-histidine to L-glutamate (histidine2) ↑	4O, 3A, 3O	R, S, W
Glycolysis and Gluconeogenesis (part) (gg13) ↑	4A, 4O, 3O	R, S

The pathways that are among the ten most relevant pathways for at least one attribute selection method (R, ReliefF; S, SVMAttributeEval; W, wrapper(naïve Bayes)) and for at least three of the four investigated datasets (4A, 'periodontal disease causing' genomes in complete dataset (266 genomes); 4O, 'periodontal disease causing' genomes in oral cavity dataset (15 genomes); 3A, 'members of red or orange cluster' in the complete dataset; 3O, 'members of red or orange cluster' in oral cavity dataset) are listed. An upwards pointing arrow denotes pathways that are relevant due to higher pathway scores (that is, pathways are more complete) for the periodontal species compared to the other genomes in the investigated dataset. In analogy, a downwards pointing arrow denotes pathways that are relevant due to lower pathway scores (that is, pathways are less complete) for the periodontal species.

#### Glutamate fermentation (fnc1), degradation of histidine to L-glutamate (histidine2), and biosynthesis of 5-formimino-THF (c2)

**Figure 9:**
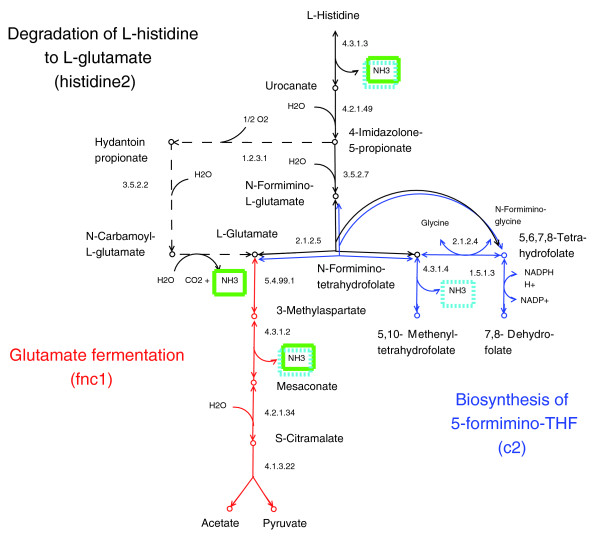
Degradation of histidine. The pathways glutamate fermentation (fnc1) (red) and degradation of histidine to L-glutamate (histidine2) (black) describe (amino acid) degradations producing ammonia as an end product. Due to the reversibility of all its reactions, this also includes the pathway of biosynthesis of 5-formimino-THF (blue), which - inversely followed - describes the degradation of 5-formimino-THF to glutamate (c2). All three pathways are interconnected and can be interpreted as complete degradation of L-histidine to acetate and ammonia (NH_3_). Thereby, three moles of ammonia per mole of histidine are produced (green or turquoise boxes, respectively). As histidine2 includes an alternative route from L-histidine to glutamate (dashed line), one mole of ammonia is either produced by the conversion of N-carbamoyl-L-glutamate to L-glutamate or by the conversion of N-formimino-tetrahydrofolate to 5,10-methenyl-tetrahydrofolate.

The three pathways glutamate fermentation (fnc1), degradation of histidine to L-glutamate (histidine2), and biosynthesis of 5-formimino-THF (c2) describe (amino acid) degradations producing ammonia as an end product and are predicted to be operative in the periodontal bacteria (except *A. actinomycetemcomitans*). Due to the reversibility of all its reactions, this also includes the pathway of biosynthesis of 5-formimino-THF, which - inversely followed - describes the degradation of 5-formimino-THF to glutamate. All three pathways are interconnected and can be interpreted as the complete degradation of L-histidine to acetate and (three moles) ammonia (NH_3_) (Figure 9). Studies by Niederman *et al*. [56] and Takahashi *et al*. [57] on ammonia as a mediator of periodontal infection support the biological relevance of our result. The authors showed that NH_3 _inhibits the polymorphonuclear leucocyte function of the host cells. It is known that this inhibition increases the susceptibility of humans to periodontal infection [58,59]. That ammonia plays an important role in periodontal disease is further supported by a study on the oral health of patients with chronic renal failure [60]. These patients show a higher prevalence of periodontal disease. Compared to healthy controls, a high concentration of urea is observed in the saliva of these patients. It is assumed that the increased amount of urea leads to an increased amount of ammonia due to the degradation of urea by urealytic oral bacteria such as *Actinomyces naeslundii*.

#### Urea cycle (part I)

The urea cycle is used by many organisms to convert toxic ammonia to urea. Some organisms (for example, most aquatic organisms), however, excrete ammonia directly [61]. According to our pathway prediction, the part of the urea cycle described by urea2 is not operative in *F. nucleatum*, *P. gingivalis*, and *T. denticola*, in contrast to other oral species. Thus, ammonia produced by periodontal species is presumably excreted directly to the host, whereas other oral species presumably metabolize the ammonia that they produce. This further supports the hypothesis that cytotoxic ammonia, to which the host's tissue is exposed, plays an important role in the development of periodontal disease.

#### Biosynthesis of coenzyme B12

Coenzyme B12 (cobalamin) plays an important role in fermentation processes of many microorganisms. In bacteria, one of these processes is the glutamate fermentation described above (fnc1). Glutamate mutase, which catalyzes the first step of glutamate fermentation (fnc1), requires B12 as a cofactor. Though B12-dependent enzymes are also known for animals and protists, biosynthesis of B12 is restricted to some bacteria and archaea. Our metabolic reconstruction predicted higher pathway scores for periodontal species compared to other oral species for this pathway. Thus, the relevance of the coba1 pathway is in good agreement with the relevance of the pathways described above [62].

#### Conversion of L-glutamate into L-proline/biosynthesis of L-proline (glutamate3/proline1), and conversion of L-glutamate to L-ornithine (glutamate2)

Both proline1 and glutamate3 describe the same universal biosynthesis of L-proline from L-glutamate (the pathway is duplicated in BioPath). glutamate2 and proline1 share the first two reactions. According to our metabolic reconstructions, these pathways are not available for *F. nucleatum*, *P. gingivalis*, and *T. denticola*. Regarding the similarity of the core metabolism between *F. nucleatum *and *Clostridia *spp., proline biosynthesis via ornithine could be an alternative route [63]. Like by the degradation of histidine, ammonia is produced by this alternative L-proline pathway.

The last relevant pathway listed in Table 6 represents a part of the glycolysis/gluconeogenesis (gg13) pathway. For this pathway, we could not find any relation to periodontal disease in the literature.

As stated above, the periodontal pathogens *T. forsythia *and *P. intermedia *have not been included in the identification of relevant pathways because they were not available in PEDANT at the time of our analysis. According to the lists of genome projects provided by the NCBI [64], the *T. forsythia ATCC 43037 *and *P. intermedia 17 *genome projects are still 'in progress'. However, their genomic sequences are now available [65]. In order to further test our results, we determined the pathway profiles for both genomes (after their automatic annotation in the new version of the PEDANT genome database [66]). For these genomes, the pathway scores of the relevant pathways (which are listed in Table 6) were largely consistent with the respective scores of *F. nucleatum*, *P. gingivalis*, and *T. denticola*. Analogous to Figure 7, Figure S16 in Additional data file 2 illustrates this metabolic similarity in the selected relevant pathways of the five out of six periodontal species.

### Comparison to related methods

The method presented here has several advantages over existing systems for relating phenotypes to the underlying biochemical processes. The current systems PUMA2 [17] and Genome Properties [16] allow for the retrieval of pathways (equivalent to genome properties in [16]) that are present in every genome of a set of prokaryotic genomes. In both systems these genomes can be selected by filtering for phenotypic traits. However, all pathways found for these genomes are listed without any information on their association with the phenotype. As a consequence, the list includes many pathways that are not typical of the traits but are also common in genomes showing other phenotypes. Without restricting the list of pathways to those that are in fact distinctive for the phenotype, the direct generation of mechanistic hypotheses - as possible using our method - is infeasible when using these systems.

In contrast to the PUMA2 and Genome Properties systems, Liu *et al*. [18] investigated pairwise associations of bacterial phenotypes with KEGG pathways. Thereby, this study considered phenotypes of bacteria given by their clinical laboratory characterizations; these characterizations are used to distinguish different microorganisms clinically (this includes morphological characteristics (for example, Gram stain, motility), metabolic functions (for example, urea hydrolysis, acetate utilization), and adaption to extreme living conditions (for example, growth at 6.5% sodium chloride)). The approach relates phenotypes to KEGG pathways [19] by mining their matching COGs [14] based on the correlation of COGs to phenotypes [20] and on the mapping of COGs to pathways. Relying on manual protein annotation as provided in the COG database restricts the applicability of this approach to the limited number of genomes that underwent time-consuming manual annotation.

Considering the rapidly growing number of completely sequenced genomes, the gap between the number of sequenced genomes and the number of manually annotated genomes will further increase. In order to overcome limitations in the number of genomes, we based our approach on completely automated genome and protein annotations provided by the PEDANT system. In spite of annotation gaps and errors, which always occur in automatic annotation processes, we achieved biologically reasonable phenotype-pathway associations (as demonstrated above). Some of the annotation errors might be compensated by the score-based metabolic reconstruction step used in our approach (in contrast, the approach by Liu *et al*. does not imply pathway prediction).

In the study by Liu *et al*., significant pairwise pathway-phenotype associations were estimated based on the (univariate) hypergeometric distribution. Applying the method to all phenotypes considered in the study (92), resulted in only 17 significant associations in total. In contrast, our approach goes beyond pairwise associations of pathways and phenotypes. In our approach, we apply machine learning methods that are based on multivariate statistics. Thus, our method also allows for the identification of pathways that are not associated with a phenotype individually but in the context of other pathways. Whereas univariate statistics assumes the pathways to be independent of each other, multivariate methods take existing dependencies among pathways into account. Recently, the advantages of considering multiple-to-one rather than pairwise associations have also been shown for gene-phenotype associations by Tamura and D'haeseleer [11].

## Discussion

While the number of completely sequenced genomes has been growing fast in recent years, our understanding of these genomes' biology has not kept up with the speed of sequencing. We already know the genomic sequences of several organisms that show certain microbial phenotypes. Nonetheless, the biochemical mechanisms associated with these phenotypes are still unclear in many cases. In order to reveal metabolic characteristics of phenotypic traits, exhaustive manual investigation and comparison of all sequenced genomes is infeasible considering the huge amount of data. Here, we demonstrate that our method is able to identify metabolic pathways relevant to phenotypic traits, while completely based on data automatically derived from genomic sequences.

As a case study, we show the applicability of our method to the well studied phenotype 'methanogenesis'. This phenotype is characterized by the production of methane as a major product of energy metabolism. So far, methanogenesis has been observed only for strictly anaerobe archaea. Apart from methanogenesis, these species are quite diverse in their metabolic capabilities.

Applying our method to the phenotype 'methanogenesis', we identified the pathway for the synthesis of methane as well as pathways typical of archaea and typical of an anaerobic lifestyle. Furthermore, we found two amino acid degradation pathways to be relevant in distinguishing methanogenic from non-methanogenic archaea. According to our metabolic reconstructions, methanogens typically lack these pathways. This might be related to the syntrophism of methanogens with amino acid degrading species. Finally, we identified the biosynthesis of the membrane phospholipid phosphatidylserine and the biosynthesis of 2'-deoxythymidine-5'-triphosphate (dTTP) as relevant pathways, which is also in good agreement with biological knowledge on methanogens. Thus, using our method, we were able to directly relate the phenotype 'methanogenesis' to known aspects of methanogenesis without giving these biological facts to our method in any form of prior knowledge. The application of our method to other well studied phenotypes, namely 'Gram-positive', 'obligate anaerobe', and 'obligate intracellular', also provided relevant pathways that reflect known metabolic characteristics of these phenotypes.

In general, one can divide phenotypic traits into two groups: traits that developed from the same origin and, thus, are mostly found in related species (for example, 'Gram-positive'); and traits that developed from different origins by adaptation to the same (for example, environmental) conditions (convergent evolution). These traits are typically found in distinct species of very different taxonomic groups. Phenotype analysis with gene-based comparative methods have mainly focused on traits that developed from the same origin.

As an example for traits that developed from different origins by adaptation to the same conditions, we analyzed the phenotype 'periodontal disease causing'. Periodontal disease is a bacterial infection of the tissues surrounding and supporting the teeth. The disease affects 50% of US adults over the age of 30 years. Taxonomically, the species causing periodontal disease (that is, showing the phenotype 'periodontal disease causing') are ordered into the phyla Fusobacteria, Bacteriodetes, Spirochaetes, and Proteobacteria.

Applying our method to the phenotype 'periodontal disease causing', we found the complete degradation of L-histidine to ammonia, acetate, and pyruvate to be relevant for the phenotypic trait. According to our metabolic reconstructions, this degradation is operative in periodontal species, while it is missing in other oral species. On the one hand, one could speculate that an increased amount of histidine in the saliva of the human host might favor the growth of periodontal species over other oral species (and finally lead to the shift in the composition of the oral flora observed in periodontal disease). On the other hand, several clinical studies suggested ammonia as a mediator of periodontal disease [56-59], as it has toxic effects on the cells in the tissue surrounding the teeth. However, many oral microorganisms - harmful and harmless - produce ammonia by degrading various amino acids. Nonetheless, considering the association of high ammonia concentrations and periodontal disease, the degradation of histidine is especially harmful (compared to the degradation of other amino acids), as it generates three moles of ammonia per mole of amino acid. Our analysis suggests that periodontal disease causing species differ from other oral species in their ability to degrade histidine. To the best of our knowledge, this difference has not been noticed or investigated before. Periodontal disease and the microorganisms associated with it have been intensively studied for decades. Throughout these studies several pathogenic factors (mainly related to adherence) have been revealed. However, medical treatment of periodontal disease still relies mostly on broad-spectrum antibiotics, killing both useful and harmful oral bacteria. The histidine degradation process could be a possible new 'target' for a more specific antibacterial treatment.

Three further hypotheses could be generated by our analysis of the phenotype 'periodontal disease causing'. First, according to our predictions, a part of the urea cycle is typically missing in periodontal species (in contrast to other oral species). Thus, periodontal species might secrete produced ammonia directly to the host instead of metabolizing it. This interpretation would further support that ammonia plays an important role in periodontal disease. Second, our analysis suggests that the metabolic processes associated with juvenile periodontitis (caused by the species *A. actinomycetemcomitans*) differ from other forms of periodontal disease. This is in good agreement with clinical studies, as the amount of *A. actinomycetemcomitans *does not correlate with most other forms of periodontal disease [52]. Third, according to our predictions, periodontal species lack the biosynthesis of proline from glutamate. The most probable alternative - the synthesis from ornithine - results in ammonia as a side product. Moreover, one could speculate that a high concentration of ornithine in saliva might increase the risk for periodontal disease. For diabetes patients, the activity of plasma arginase is increased compared to the healthy control [67]. As arginase catalyzes the hydrolysis of L-arginine to L-ornithine and urea, its increased activity might lead to a higher concentration of ornithine in blood. Assuming that the concentration of ornithine is also increased in human saliva, one could further speculate that the prevalence of diabetes patients to periodontal disease is connected to the alternative (bacterial) proline synthesis. Several clinical studies that related increased arginase activity in the salivary glands to periodontal disease [68,69] further support this hypothesis.

The examples of methanogenesis and periodontal disease demonstrate that our method - though completely based on automatically predicted data - is able to map phenotypic traits directly onto metabolic processes. In the case of the phenotype 'periodontal disease causing' we could generate several meaningful hypotheses. Of course, these hypotheses can only give hints as to further experimental or clinical investigations (statistical relevance of metabolic processes for phenotypic traits does not necessarily imply causality). Moreover, our approach focuses on metabolic similarities of phenotypically related species. Thus, it can only reveal pathogenic mechanisms that are in common for the majority of the periodontal species rather than specific for a single species, and that are related to (qualitative) metabolic characteristics (covered by the pathway database used) rather than regulatory effects. As an example, pathogenic mechanisms such as the increased production of a leukotoxin as result of a deletion in the ltx operon of *A. actinomycetemcomitans *[70] could not be discovered by our method.

In general, our automatic method for hypothesis generation has several limitations that we have to bear in mind when interpreting its results. First, we use automatically derived protein annotations and data that have been automatically derived from genomic sequences usually contain errors, gaps, and inconsistencies. Thus, by using these data for pathway predictions - as we did in our approach - we might miss or overpredict pathways. However, compared to other methods for finding phenotype-metabolism associations, our method should compensate for parts of wrong or missing functional protein annotation as it focuses on metabolic pathways rather than individual genes. Nonetheless, using more reliable, manual protein annotation would improve the reliability of our pathway predictions. Second, we use EC numbers for mapping proteins onto reactions: however, several enzymes lack EC numbers though the reactions that they catalyze are well known already. By using EC numbers for protein-reaction mapping, we miss these reactions, restricting the utility of our method to pathways that involve reactions and enzymes with EC numbers assigned. Third, we use similarity-based EC number predictions: although we received biologically reasonable results based on the comparatively simple, similarity-based procedure for automatic EC number predictions, using more sophisticated procedures might further improve our results. Fourth are limitations with respect to the pathways available: our method is limited to the current biochemical knowledge or - more precisely - to the metabolic knowledge represented in the pathway database that we use. As an example, a phenotypic trait could be related to a specific toxin, for which we do not know the synthetic pathway yet. Since we based our analyses on a relatively small pathway collection, the usage of a more comprehensive pathway database such as MetaCyc could complement our results. Fifth are limitations with respect to the genomic sequences available: the type of species sequenced so far is highly biased towards cultivable and disease-related species. This might influence our results.

Our method is generic regarding both the type of phenotypic traits to analyze and the set of genomes used for analysis. Thus, in principle, our method can be applied to arbitrary phenotypes using arbitrary sets of genomes (including newly sequenced genomes). However, traits such as 'habitat: soil' seem to be too unspecific to get relevant pathways by our method. This might be due to the existence of many different environmental conditions covered by this complex habitat (for example, aerobe/anaerobe conditions, different nutrition). Furthermore, syntrophic relationships (that is, metabolic interconnection) of species in microbial communities might complicate the analysis.

## Conclusions

Here, we describe a novel method for identifying metabolic pathways that are distinctive of a complex microbial phenotype and, thus, for revealing the representative biochemical features of phenotypically related microorganisms. Relying on functional (metabolic) entities rather than individual genes, our approach allows for the direct generation of hypotheses about the biochemical basis of phenotypic traits.

The potential of our method has been shown for experimentally intensively studied phenotypes such as methanogenesis, Gram-positivity, obligate anaerobicity, and obligate intracellularity. For these phenotypes, our approach was able to identify biologically reasonable pathways whose association with the phenotype is confirmed by experimental evidence. In contrast, periodontal disease is less well investigated as a microbial phenotype, though it has been intensively studied with respect to clinical aspects. Using the example of periodontal disease, we demonstrate that our approach is well suited to generate new, biologically relevant hypotheses. Our new finding - that periodontal species share the ability to degrade histidine - is supported by the results of several clinical studies and can now be used to inspire new experiments.

Our method is based on the completely automated analysis of genome data. It is generic and thus applicable to any phenotype and to thousands of genomes yet to be sequenced as a result of high-throughput sequencing technologies. This also includes species and phenotypes whose biochemistry is largely unknown so far. The number of species that are not accessible by experiments due to their lifestyle but for which the genomic sequence is available will also grow enormously in the next years. Thus, the automatic linking of phenotypes to associated metabolic processes, as provided by our method, will be highly valuable for the interpretation of genome information.

## Materials and methods

### Genomic information

For our approach, access to a large number of automatically annotated genomes is crucial. In principle, our method can use annotations from a wide range of different annotation systems and collections because it relies on EC number annotations, which are provided by most of the systems available to date. However, standardized automatic annotations (that is, annotations that have been derived by the same means for all genomes) are generally preferable as a basis for comparative analyses. For our analyses, we chose the PEDANT genome database. PEDANT provides exhaustive standardized automatic analyses for a huge number of genomic sequences by a large variety of established bioinformatics tools [23,71]. In March 2006, PEDANT contained 266 completely sequenced genomes from all domains of life (mitochondria, chloroplasts, and genomes lacking some of their plasmids were not considered).

After identifying the proteins of a newly sequenced genome, PEDANT predicts EC numbers by BLAST similarity searches [72] against the current public non-redundant set of protein sequences. Thereby, BLAST hits up to an e-value of 0.01 are stored within PEDANT. PEDANT assigns the EC numbers of all BLAST hits with e-value < 0.00001. However, Rost [73] showed that similarities in short regions and the transfer of annotations for different domains often cause wrong EC annotations. Rost demonstrated that a score relating sequence identity to alignment length outperformed BLAST e-values. Therefore, we used transferred EC annotations based on all BLAST hits stored within PEDANT that we further filtered for hits fulfilling:

$$\begin{array}{ccc} \frac{\%seqIdent*length(alignment)}{length(aa\;\;sequence\;\;hit)}\geq t & with & 0\leq t\leq 100 \end{array}$$

For our analyses, we used *t *= 12. To estimate the prediction quality, we compared the EC numbers predicted by PEDANT to manual EC assignments for *Protochlamydia amoebophila UWE25*, *Listeria monocytogenes EGD*, *Listeria innocua Clip11262*, and *Saccharomyces cerevisiae *(manual assignments available within PEDANT). For these four genomes, the sensitivity (that is, ratio of correctly predicted EC numbers to all manually assigned EC numbers) is 75%, 86%, 87%, and 73%, respectively. The positive predictive value (that is, the ratio of correctly predicted EC numbers to all predicted EC numbers) is 84%, 79%, 79%, and 76%, respectively. The quality of EC prediction was not sensitive for a wide range of *t *(6 ≤ *t *≤ 18).

### Phenotypic information

Based on phenotypic descriptions provided by the NCBI Genome Project [64], by the EBI Integr8 database [74], and by Karyn's Genomes [75], we collected information about presence or absence of different phenotypic traits to the 266 PEDANT genomes. In particular, the following phenotypic features were considered: Gram stain (Gram-positive; Gram-negative); oxygen usage ((obligate) aerobe; (obligate) anaerobe; facultative); special metabolism (methanogenic); pathogenic (causing periodontal disease; causing caries); habitat (soil; oral cavity); intracellularity (obligate; facultative) (Additional data file 1). For our analyses we restricted ourselves to simplified two-class phenotypes (for example, methanogenic; non-methanogenic).

### Reaction and pathway information

Information on biochemical reactions and known reference pathways were taken from the free, publicly available Biochemical Pathways database (BioPath) [30,76]. BioPath contains data derived from the Roche Applied Science Biochemical Pathways wall chart. About 2,000 reactions are organized in 68 global pathways, providing a generic view of the metabolism of different organisms. The global pathways (for example, histidine metabolism) are divided into 306 smaller pathways according to different processes (for example, degradation of histidine to glutamate) or phases of the global pathways. We excluded 16 of the 306 pathways for our study as they contain only reactions for which no EC number is assigned. Similar to KEGG [19], BioPath provides a generic, multi-organism view of pathways. This means that in BioPath the reference pathways include different enzyme variants. In contrast to KEGG, these pathways are defined in smaller sets (5.4 reactions per reference pathway, on average, in BioPath compared to 21 in KEGG) since different biological processes such as degradation and biosynthetic processes correspond to separate reference pathways in BioPath. For our purposes, pathway maps in KEGG summarize too many different metabolic functions in one unit (for example, 'arginine and proline metabolism').

BioPath reference pathways describe metabolic capabilities rather than specific pathway variants. Thus, using BioPath for our analyses, our method identifies metabolic similarities of phenotypically related species with respect to degrading or biosynthetic capabilities. Thereby, differences related to enzyme variants are neglected. In contrast, basing our analyses on MetaCyc [29] reference pathways as such would change the outcome of our method towards phenotype-related pathway variants. In order to get the same type of outcome, MetaCyc reference pathways describing variants of the same metabolic conversion could be merged into a single reference pathway in a preprocessing step.

### Metabolic reconstructions represented by pathway profiles

We determined the metabolic complement of each genome by a fully automated metabolic reconstruction algorithm. This algorithm is divided into two steps. In the first step, we selected all reactions that can be catalyzed by at least one of the genomes' gene products. This selection was based on the filtered EC hits for the gene products provided by PEDANT and the EC numbers of biochemical reactions contained in BioPath. The gene products were mapped onto the corresponding biochemical reactions via matching EC numbers. This implies that we could not map gene products onto enzymatic reactions without EC number assignments. For pathways containing such reactions the enzymes were treated as missing in the genome.

In the second step, we determined a score for each reference pathway *p *out of all 290 BioPath pathways considered in our analyses. The higher the score, the more probable it is for the pathway *p *(and thus the corresponding metabolic capability) to be present in a genome. The *score(p) *depends on both the completeness of *p *and the uniqueness of the involved reactions among all defined pathways. The presence of an enzymatic reaction in *p *is weighted by the number of occurrences of this reaction in all known pathways in the database. The sum of these weighted values for all reactions in *p *is normalized to scores ranging from 0 (no reaction of the pathway is catalyzed) to 1 (pathway is complete). For the normalization, this sum is divided by the maximum sum, representing the case that all reactions in *p *are available in the genome.

$$\begin{array}{lll} score(p)=\frac{\sum_{rep}\frac{k(r)}{occ(r)}}{\sum_{rep}\frac{1}{occ(r)}} & with & k(r)=\{\begin{array}{ll} 1 & if\;enzyme\;available\;for\;reaction\;r\\ 0 & if\;enzyme\;not\;available\;for\;reaction\;r \end{array}\\ & and & \begin{array}{cc} occ(r) & occurrences\;of\;reaction\;in\;all\;pathways \end{array} \end{array}$$

The metabolic reconstruction of each genome was characterized by its pathway profile defined as the vector of pathway scores for all considered reference pathways in BioPath (290).

For pathways containing reactions without EC number assignments, the maximal pathway score is always (that is, for any genome) below 1.0 because the corresponding enzymes are treated as missing. This does not allow for a reliable prediction of the presence or absence of the respective pathway when a fixed score threshold is used for the prediction (see below). Nonetheless, the relative differences of the scores determined for the genomes (according to the remaining enzymes) are taken into account when comparing score-based pathway profiles as done in our approach for uncovering phenotype-related pathways.

In order to evaluate the score-based metabolic reconstructions, we used the pathway reconstruction score to predict the presence or absence of pathways in genomes. Thereby, we predicted the presence of a pathway if the pathway received a score of at least 0.5. It is important to note that we applied this threshold only for evaluation purposes. In our approach for uncovering phenotype-related pathways, we directly compared the pathway profiles containing the score values instead of binary values for the presence or absence of pathways.

For the evaluation, we determined the pathway reconstruction scores for the well studied pantothenate pathway in the completely sequenced genomes provided by PEDANT. For 77 out of 78 genomes for which we found information on this pathway in the literature, our predictions have been correct. For two further well studied pathways - the mevalonate pathway and the Embden-Meyerhof pathway - we manually compared the EC number assignments of PEDANT to the literature for nine model organisms. In order to study the influence of automatic EC number prediction on the pathway prediction for BioPath pathways, we also compared pathway predictions based on automatically annotated EC numbers to predictions that were based on manually annotated EC numbers. Additionally, we judged the quality of our metabolic reconstructions for the genomes of *M. jannaschii *and *Escherichia coli K12 *by comparing them to previous reconstructions and the manually curated EcoCyc database [77], respectively. Detailed descriptions of the complete evaluation procedure and the results obtained are given in Additional data file 3.

### Pathway/attribute selection

In general, attribute selection denotes the identification of attributes (here, pathways) that are most relevant (informative) in distinguishing instances (here, genomes) with different class labels (here, phenotypic traits). Attribute selection can rely on univariate and multivariate statistics. Univariate methods (for example, chi square, pearson correlation) look at the relevance of individual attributes for a specific class at a time. Thereby, attributes are assumed to be independent. However, attributes that are not individually relevant for the class may become relevant in the context of other attributes. Multivariate methods take this context into account, that is, subsets of attributes are selected rather than individual attributes. Thus, attributes are not assumed to be independent by multivariate methods. As we expected dependencies of pathways for complex phenotypic traits, we used multivariate methods for our approach.

Technically, three general strategies, namely filters and wrappers and embedded methods exist to evaluate the worth of attribute subsets [78]. Filters remove irrelevant attributes based on general characteristics of the data. Wrappers, on the other hand, evaluate attribute subsets by using accuracy estimates provided by a certain classification algorithm. Thus, individual needs of the classification method are taken into account [79]. Embedded methods are also specific to a given learning machine. But these methods select attribute subsets during the training of the learning machine, whereas in wrappers the classifier is only used as a black box to score the predictive power of attribute subsets.

For our studies, we used the implementations of attribute subset selection methods found in the Waikato Environment for Knowledge Analysis suite (Weka, Version 3.5.6) [80]. Weka is a free tool based on Java. It provides a large number of machine learning algorithms and data pre-processing methods presented in a graphical user interface for data exploration and visualization. Weka has proven to be useful in bioinformatics [81,82].

We used three different attribute subset selection methods: the filter method ReliefF [34-36], a wrapper method [38] relying on a naïve Bayes classifier [83], and the embedded method SVMAttributeEval [37]. In order to avoid overfitting, we applied tenfold cross-validation in the ReliefF and SVMAttributeEval approaches, and fivefold cross-validation in the wrapper approach.

#### ReliefF

ReliefF assigns a (relevance) weight to each attribute according to how well their values distinguish among instances of different classes and according to how well they cluster instances of the same class. The algorithm repeatedly chooses a random instance from the data. For this instance the nearest instances of the same class and the nearest instances belonging to other classes are determined. The attribute weights are updated based on the differences between the selected instance and its nearest neighbors. ReliefF does not remove statistically dependent attributes. As stated above, most multivariate methods rank subsets of attributes rather than individual attributes. However, ReliefF relies on a multivariate relevance criterion that allows the ranking of individual attributes according to their relevance in the context of other attributes.

#### SVMAttributeEval

SVMAttributeEval combines a (linear) support vector machine (SVM) [84] (for a brief description see below) and the technique of recursive feature elimination (RFE) in order to assess the relevance of attribute subsets [37]. RFE is an iterative process of training a classifier (by optimizing the attribute weights for the cost function), computing the attribute ranking criterion for each attribute (by determining the change in cost function when omitting the attribute), and removing the attribute with the smallest ranking criterion. A linear SVM represents the classifier in the case of SVMAttributeEval. SVMAttributeEval results in a complete ranking of attributes; thereby, the ranking corresponds to nested subsets of attributes.

#### Wrappers

Wrappers analyze the data in the context of a certain classifier. For our studies, we used the naïve Bayes classifier (for a brief description see below). Wrappers train a classifier based on an attribute subset and score the predictive power of the subset by cross-validation. An exhaustive search for the best subset among all possible attribute subsets is very complex due to the high number of possible subsets. Therefore, heuristic methods are used instead. Here, we applied the best first search as a heuristic search strategy. In contrast to ReliefF and SVMAttributeEval, a complete ranking of all attributes is unavailable for the wrapper approach. After fivefold cross-validation, Weka prompts how often each of the attributes was part of the most relevant subset throughout the five runs (percentage). For a more thorough description of the utilized attribute subset selection methods see [78,80,85].

If not stated otherwise, we applied the default parameters set within Weka for each of the methods. We are aware that the outcome of attribute selection and classification can be optimized by adjusting the parameters. In particular, the outcome of SVMs heavily depends on parameter settings. Thus, parameter optimization might further improve our results.

### Cross-check of relevant pathways by classification

In order to estimate the significance of the identified relevant pathways for the phenotype, we cross-checked the results of attribute subset selection by classification. For this purpose, we classified the genomes represented by pathway profiles that contained only the scores for the 1, 2, 3, ..., 20 pathways with highest ranks. The resulting classification quality was compared to the classification quality achieved using all pathways (290) and randomly selected sets of 1-20 pathways. For estimating the random classification quality, we used 25 sets containing 2 randomly selected pathways and calculated the average quality, then used 25 sets containing 3 randomly selected pathways and calculated the average quality and so forth up to 25 sets containing 20 randomly selected pathways.

The implementation of four different classification algorithms - J48, IB1, naïve Bayes, and SMO - in Weka were used in order to avoid effects caused by biases of classifiers. J48 is based on pruned or unpruned C4.5 [86] decision trees learned by the algorithm. C4.5 splits the input data into smaller subsets by iteratively choosing individual attributes for the decision based on their information gain. IB1 [87] is a nearest neighbor classifier based on normalized Euclidean distance. The class of its nearest neighbor is assigned to an instance. Naïve Bayes is a probabilistic classifier [83] that applies a simplification of Bayes' law by (naïvely) assuming the independence of attributes. The (posterior) probability of a class for the given attributes of an instance is derived based on the (prior) probability of the class and the likelihood of the instance given the class. Prior probability and likelihood are estimated by the corresponding frequencies in the training data. SMO implements the sequential minimal optimization algorithm for training a SVM [88]. A SVM is looking for a hyperplane that is an optimal boundary between the classes (that is, a boundary that maximizes the surrounding margin containing no instance). The model is represented by so-called support vectors, a small set of instances that are sufficient to determine the boundary. If linear separation is not possible, the data can be transformed to a higher dimensional space by so-called kernel functions in order to allow linear separation. Here, we used a linear SVM.

After tenfold cross-validation, we multiplied the resulting selectivity and sensitivity for each classification performed. Here, this product is referred to as classification quality. We decided to use this measure because the product of selectivity and sensitivity is very sensitive to either of selectivity or sensitivity being low. Determining the ROC AUC is another possibility to assess classification quality, while considering both selectivity and sensitivity. This measure has often been used to compare the quality of different classifiers [89,90]. Formally, the ROC AUC is equivalent to the Mann-Whitney or Wilcoxon non-parametric test [91]. For a two-class (positive/negative) classification problem, the value indicates the probability that the classifier produces a higher score for a randomly picked positive instance than for a randomly picked negative instance. An ROC AUC value of 0.5 corresponds to the value reached by random guess. Usually, values above 0.7 represent statistically reasonable classification quality. (For an introduction to ROC analysis, see [92].) The ROC AUC values for all classifications performed are shown in Additional data file 2.

We applied the default parameter settings given within Weka for each classifier. We are aware that the optimization of the parameters could improve the classification quality. However, our main aim of classification was to estimate the significance of the selected pathways rather than optimal classification quality.

For each classification algorithm used, we plotted the classification quality for each attribute subset selection method depending on the number of pathways taken into account. The corresponding random classification quality values are given in the same diagram. A horizontal line marks the classification quality achieved when using all pathways for classification. In order to estimate the significance of the relevant pathways, the resulting four diagrams are checked manually. If the following holds true for at least one of the four diagrams, we assume that the phenotype under consideration is related to the selected relevant pathways: the classification quality is considerably better than random; the classification quality reaches at least the quality achieved when using all pathways for classification; the classification quality (assessed by the product of sensitivity and selectivity) reaches at least 0.6. For plotting the classification quality diagrams, we used the R statistical software package (version 2.5.1) [93].

### Availability

All software developed in the scope of the work presented and all data used in our study are freely available for non-commercial, academic research purposes. The software consists of a Java application (command line) for pathway scoring and a Perl script for pathway selection and cross-checking (using the free tools Weka and R). The software and the data can be downloaded free of charge from our website [94].

## Abbreviations

AUC: area under the curve; COG: Clusters of Orthologous groups of proteins; EC: Enzyme Commission; KEGG: Kyoto Encyclopedia of Genes and Genomes; ROC: receiver operating characteristic; SVM: support vector machine.

## Authors' contributions

GK, JG, and HWM were involved in conceiving the study. GK implemented the method, performed the data analysis and contributed to the evaluation of the metabolic reconstruction method. MES performed the evaluation of the metabolic reconstruction method. GK drafted the main manuscript. All authors read, contributed to, and approved the final manuscript.

## Additional data files

The following additional data are available with the online version of this paper: a table listing the completely sequenced genomes included in our analyses and the genomes' phenotypic traits considered (Additional data file 1); a PDF document containing the classification quality diagrams for all phenotypes mentioned in the manuscript and lists of the resulting relevant pathways (Additional data file 2); a PDF document containing detailed descriptions of the procedure used for evaluating the automatic metabolic reconstruction part of our method and the evaluation results obtained (Additional data file 3).

## Supplementary Material

Additional data file 1 Completely sequenced genomes included in our analyses and the genomes' phenotypic traits. Click here for file

Additional data file 2 Classification quality diagrams for all phenotypes and the resulting relevant pathways. Click here for file

Additional data file 3 The procedure used for evaluating the automatic metabolic reconstruction part of our method and the evaluation results obtained. Click here for file
