# Supplementary material for: Uncovering metabolic pathways relevant to phenotypic traits of microbial genomes
Source: Genome Biol. 2009 Mar 10;10(3):R28. doi: 10.1186/gb-2009-10-3-r28 (PMC2690999; doi:10.1186/gb-2009-10-3-r28)
Supplement: Additional data file 2 — Classification quality diagrams for all phenotypes and the resulting relevant pathways. [file gb-2009-10-3-r28-S2.pdf]

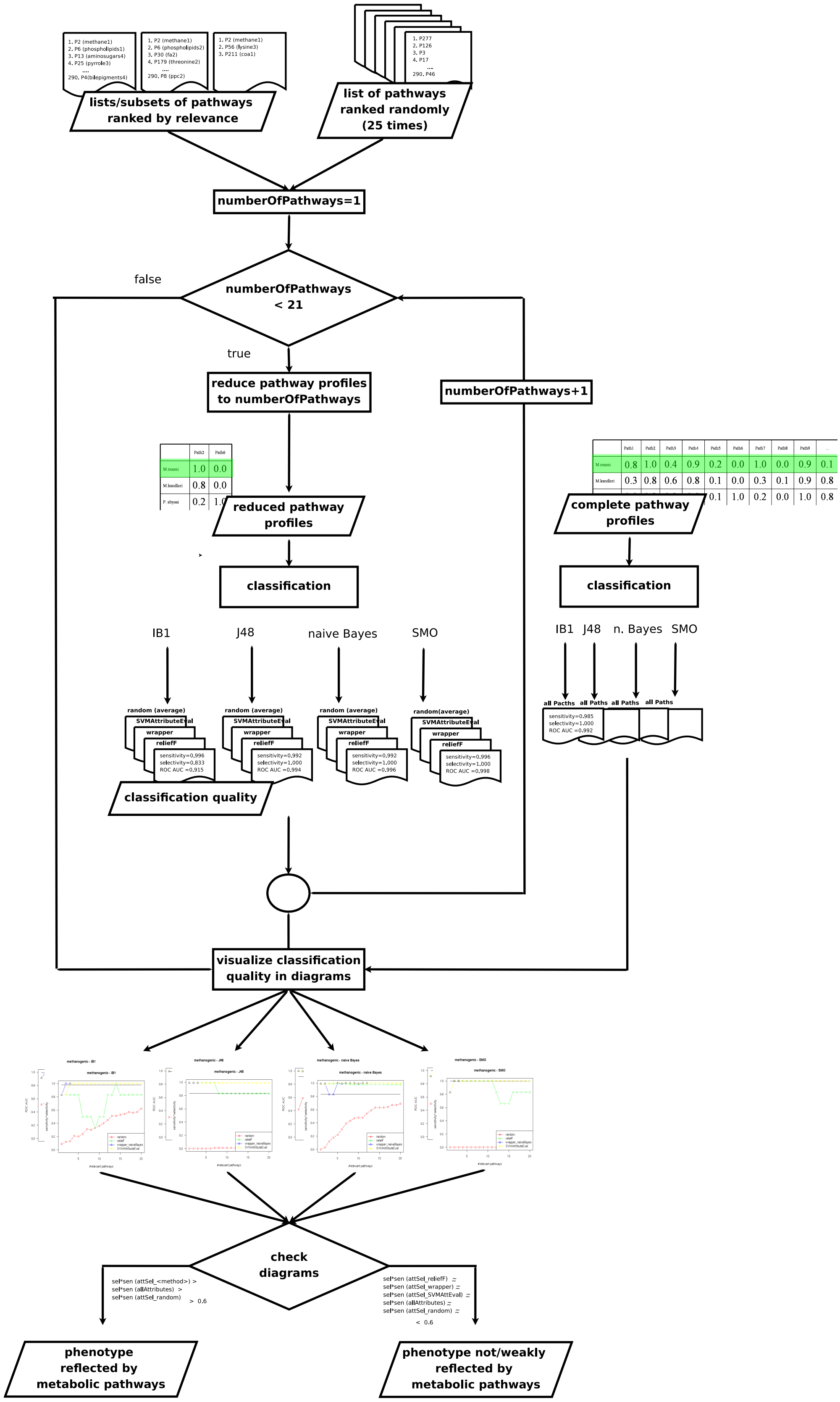

Figure S1: Overview of the Cross-checking Process. For each phenotype, the rankings of relevant pathways, which result from attribute selection, were cross-checked by classifying the genomes into those showing the phenotype an those lacking it. For this classification, we only considered the best ranking one to twenty pathways (i.e. the corresponding reduced pathway profiles), respectively. In order to estimate the significance of the top-ranking pathways, we compared the achieved classification quality to the quality reached by classification based on all pathways (i.e. the complete pathway profiles) and based on randomly chosen one to twenty pathways.

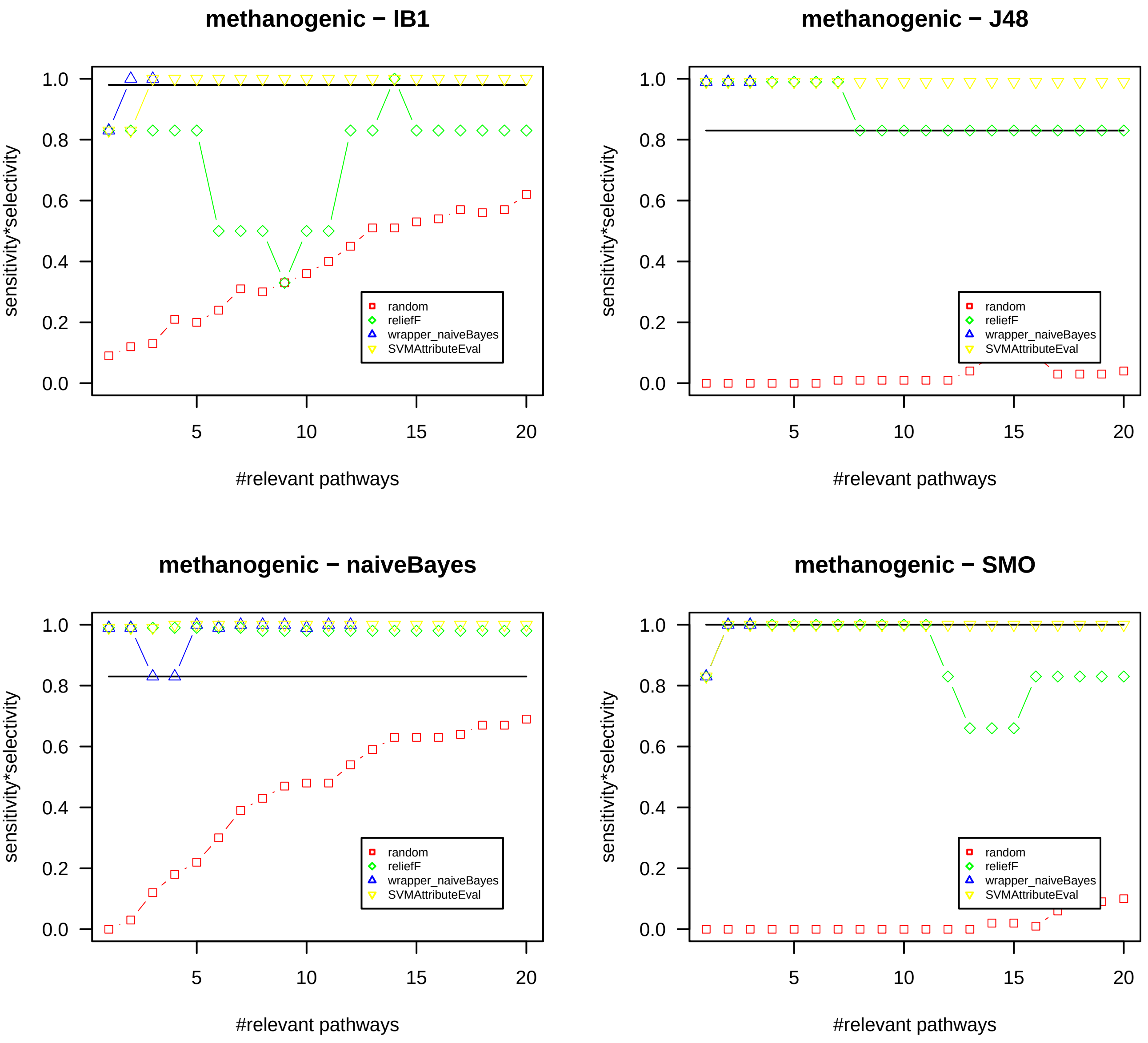

Figure S2: Cross-checking the Most Relevant Pathways for Methanogenesis by Classification. The diagrams show the classification quality (assessed by the product of sensitivity and selectivity) for the classification of the completely sequenced genomes (266) into methanogens and non-methanogens. The classification is based on reduced pathway profiles containing only the 1 to 20 most relevant pathways (green: ReliefF, yellow: SVMAttributeEval, blue: wrapper (naive Bayes)), respectively. The classification quality achieved for classification based on all (290) pathways is marked by a horizontal line (black). Red boxes depict the quality of classification based on randomly chosen 1 to 20 pathways (average quality for 25 times). For classification, we applied the nearest neighbor classifier IB1, the decision tree classifier J48, the naive Bayes classifier, and the linear support vector machine SMO. The diagrams demonstrate that the identified most relevant pathways are well suited to distinguish methanogens and non-methanogens (max(sensitivity*selectivity) = 1.0). According to the cross-check, the most relevant pathways identified by attribute selection are considered as significant (see Methods). Apart from using ReliefF top-ranking pathways (green) for the classification with IB1, the maximum classification quality is already reached for the (up to) five most relevant pathways. These pathways are listed in Table 1. The increase in classification quality for the rising number of randomly picked pathways (IB1, naive Bayes) indicates that the over-all metabolism is similar within the group of methanogens and distinctive when compared to the group of non-methanogens.

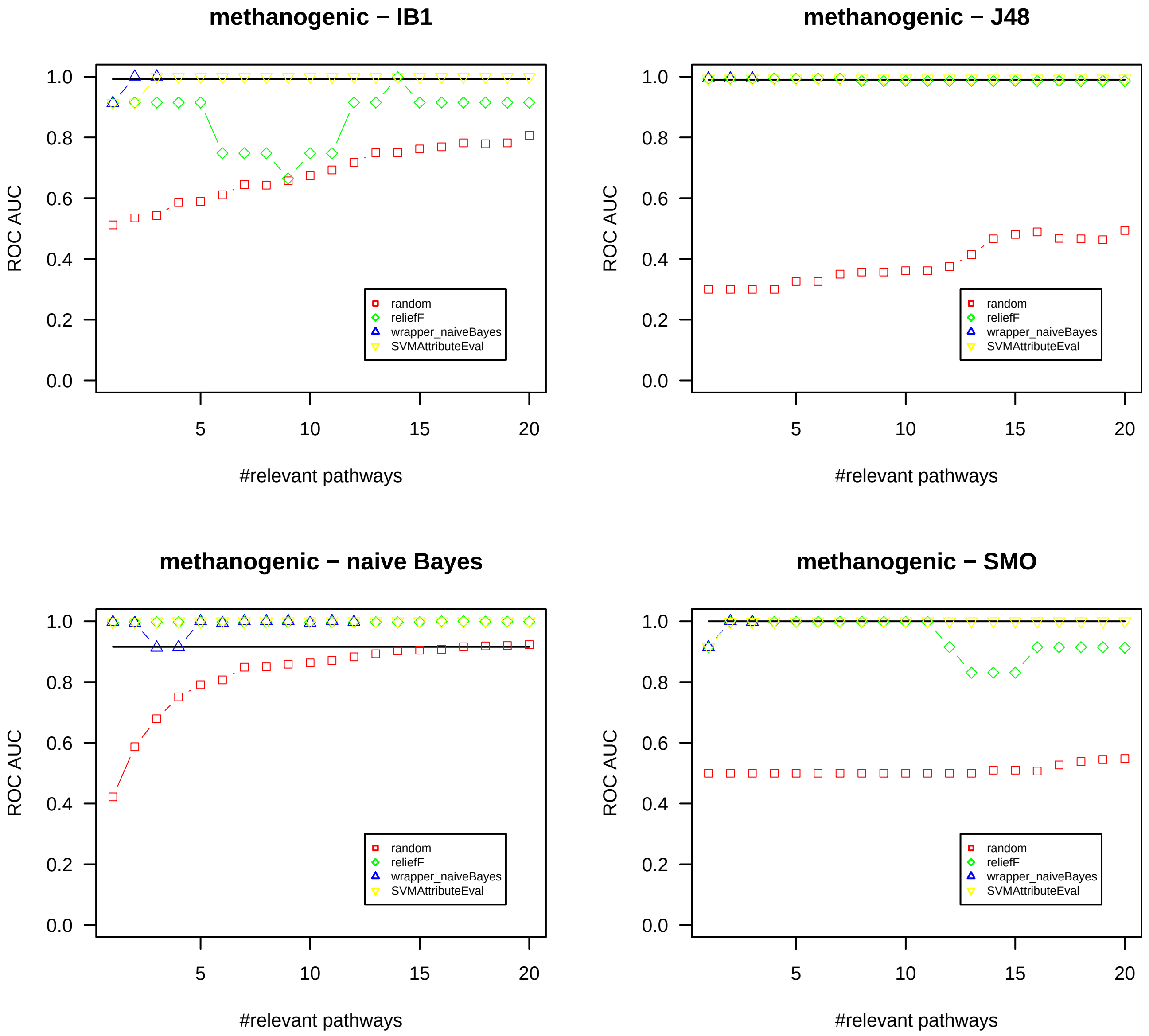

Figure S3: Cross-checking the Most Relevant Pathways for Methanogenesis by Classification Using ROC AUC Values. The diagrams (266) show the classification quality (assessed by the ROC AUC value) for the classification of the completely sequenced genomes into methanogens and non-methanogens. The classification is based on reduced pathway profiles containing only the 1 to 20 most relevant pathways (green: ReliefF, yellow: SVMAttributeEval, blue: wrapper (naive Bayes)), respectively. The classification quality achieved for classification based on all (290) pathways is marked by a horizontal line (black). Red boxes depict the quality of classification based on randomly chosen 1 to 20 pathways (average quality for 25 times). For classification, we used the nearest neighbor classifier IB1, the decision tree classifier J48, the naive Bayes classifier, and the linear support vector machine SMO. The diagrams demonstrate that the identified most relevant pathways are well suited to distinguish methanogens and non-methanogens (max(ROC AUC) = 1.0). The classification quality achieved by J48 and SMO is about 0.5 corresponding to random classification. For classification using naive Bayes only 17 randomly picked pathways are sufficient to reach the same classification quality as achieved when considering all (290) pathways. This indicates that the over-all metabolism of methanogens is similar within the group of methanogens but distinctive compared to non-methanogens.

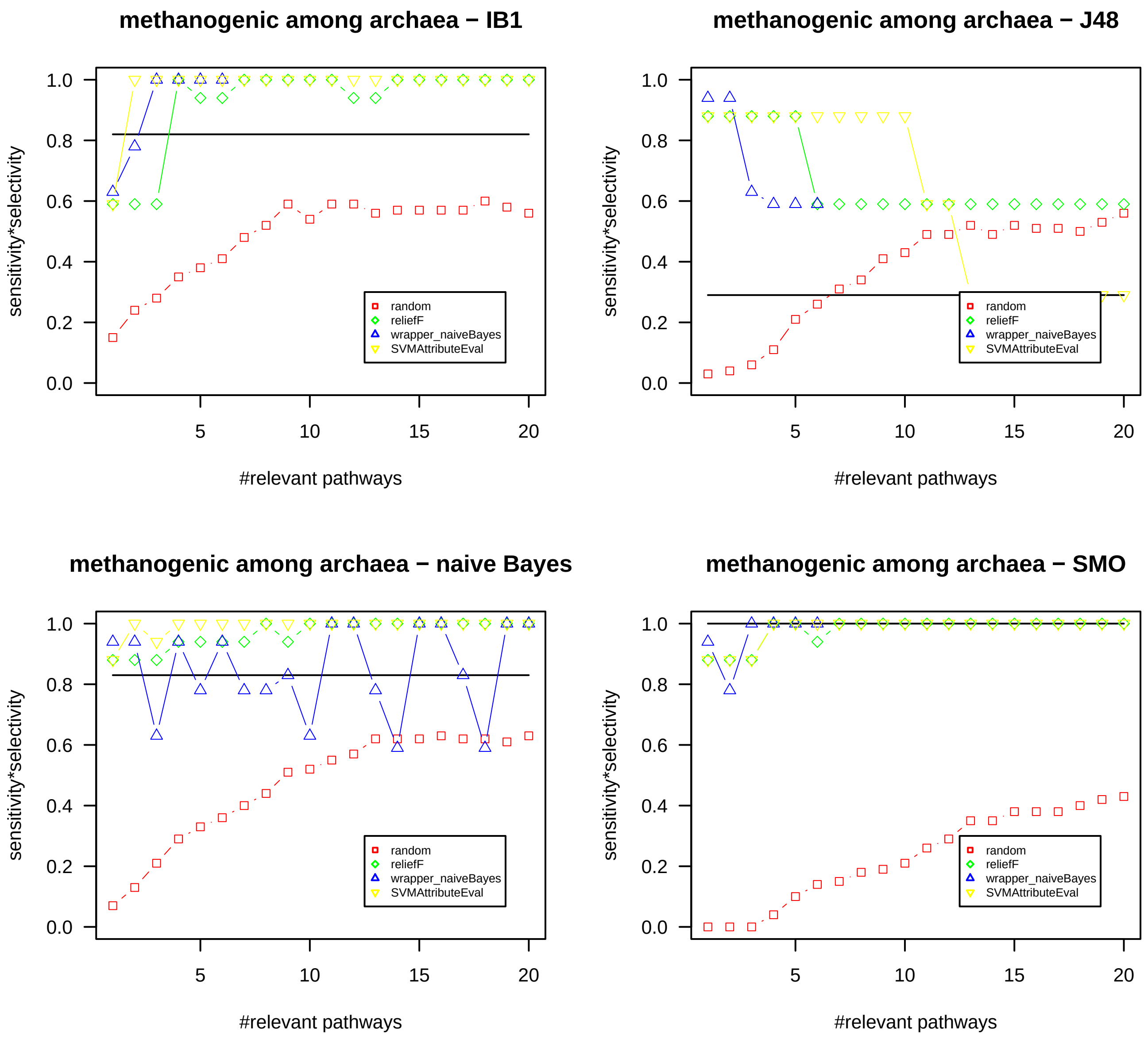

Figure S4: Cross-checking the Most Relevant Pathways for Methanogenesis among Archaea by Classification. The diagrams show the classification quality (assessed by the product of sensitivity and selectivity) for the classification of completely sequenced archaea (23) into methanogens and non-methanogens. The classification is based on reduced pathway profiles containing only the 1 to 20 most relevant pathways (green: ReliefF, yellow: SVMAttributeEval, blue: wrapper (naive Bayes)), respectively. The classification quality achieved for classification based on all (290) pathways is marked by a horizontal line (black). Red boxes depict the quality of classification based on randomly chosen 1 to 20 pathways (average quality for 25 times). For classification, we used the nearest neighbor classifier IB1, the decision tree classifier J48, the naive Bayes classifier, and the linear support vector machine SMO. The diagrams demonstrate that the identified most relevant pathways are well suited to distinguish methanogenic and non-methanogenic archaea (max(sensitivity*selectivity) = 1.0). According to the cross-check, the most relevant pathways identified by attribute selection are considered as significant (see Methods). The diagrams demonstrate that the identified most relevant pathways are well suited to distinguish methanogenic and non-methanogenic archaea. These pathways are listed in Table 1. For all classifiers, the classification quality is remarkably enhanced when considering only the 1 to 5 most relevant pathways compared to the quality achieved considering all or randomly picked pathways.

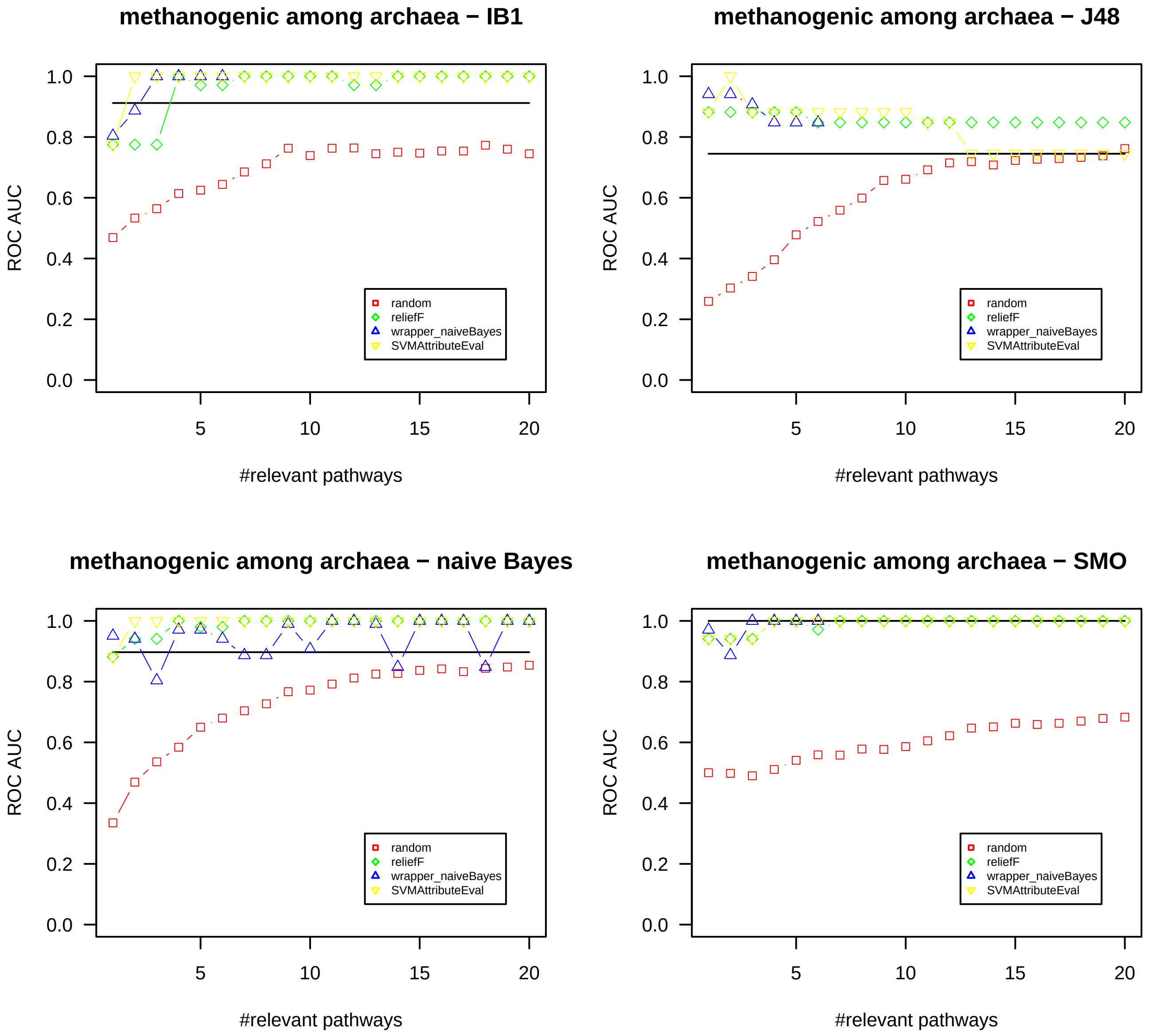

Figure S5: Cross-checking the Most Relevant Pathways for Methanogenesis among Archaea by Classification Using ROC AUC Values. The diagrams show the classification quality (assessed by the ROC AUC value) for the classification of completely sequenced archaea (23) into methanogens and non-methanogens. The classification is based on reduced pathway profiles containing only the 1 to 20 most relevant pathways (green: ReliefF, yellow: SVMAttributeEval, blue: wrapper (naive Bayes)), respectively. The classification quality achieved for classification based on all (290) pathways is marked by a horizontal line (black). Red boxes depict the quality of classification based on randomly chosen 1 to 20 pathways (average quality for 25 times). For classification, we used the nearest neighbor classifier IB1, the decision tree classifier J48, the naive Bayes classifier, and the linear support vector machine SMO. The diagrams demonstrate that the identified most relevant pathways are well suited to distinguish methanogenic and non-methanogenic archaea (max(ROC AUC) = 1.0).

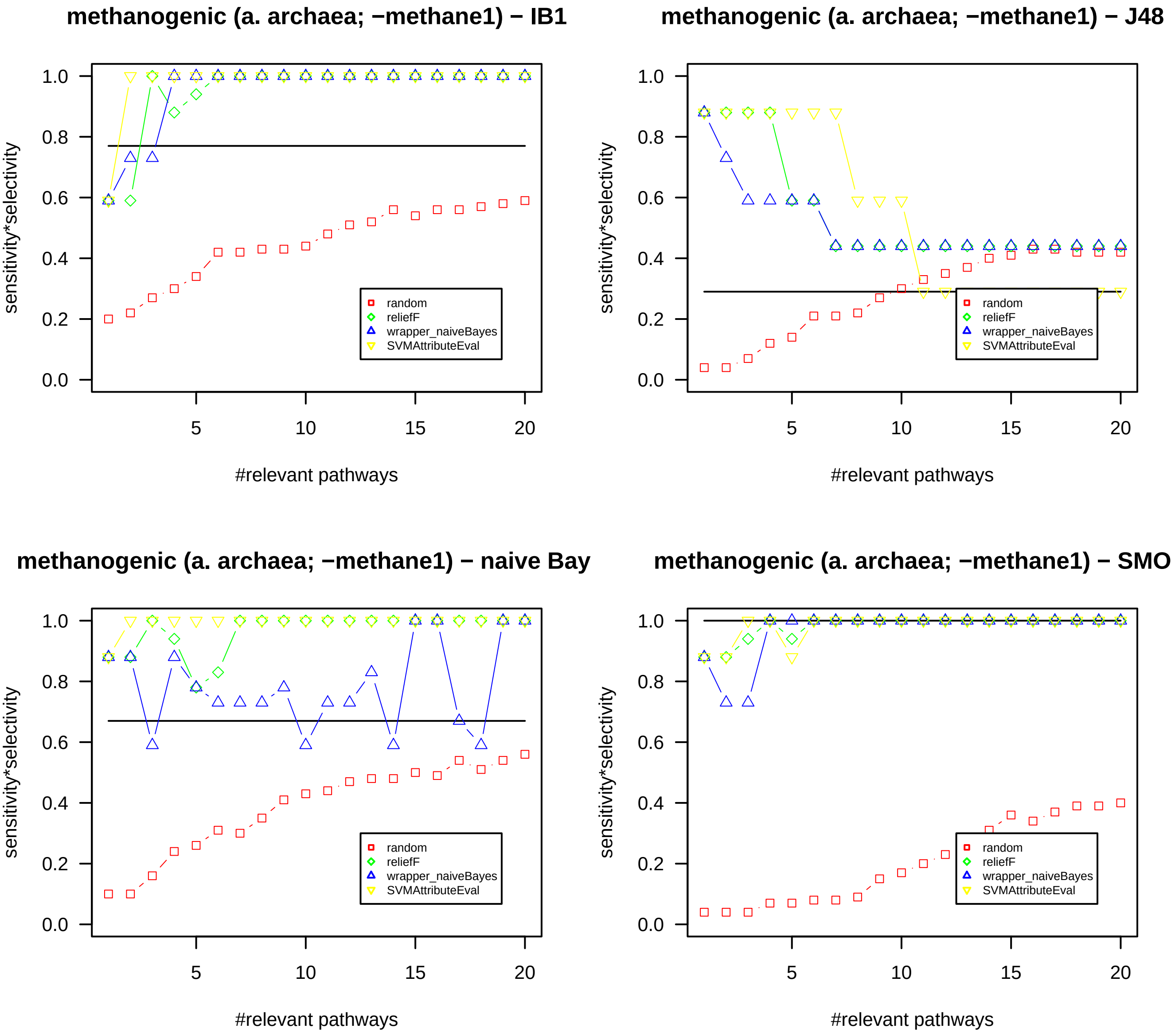

Figure S6: Cross-checking the Most Relevant Pathways for Methanogenesis among Archaea by Classification Disregarding the Methane Pathway. The diagrams show the classification quality (assessed by the product of sensitivity and selectivity) for the classification of completely sequenced archaea (23) into methanogens and non-methanogens. The classification is based on reduced pathway profiles containing only the 1 to 20 most relevant pathways (green: ReliefF, yellow: SVMAttributeEval, blue: wrapper (naive Bayes)), respectively. Thereby, attribute selection was performed on pathway profiles without the pathway for methane production. Thus, the most relevant pathways, on which classification is based, do not contain this pathway. The classification quality achieved for classification based on all (290) pathways is marked by a horizontal line (black). Red boxes depict the quality of classification based on randomly chosen 1 to 20 pathways (average quality for 25 times). For classification, we used the nearest neighbor classifier IB1, the decision tree classifier J48, the naive Bayes classifier, and the linear support vector machine SMO. The diagrams demonstrate that the identified most relevant pathways (listed in Table 1) are well suited to distinguish methanogenic and non-methanogenic archaea (max(sensitivity*selectivity) = 1.0), though the most characteristic metabolic pathway for methanogenesis (methane1) is not considered for classification.

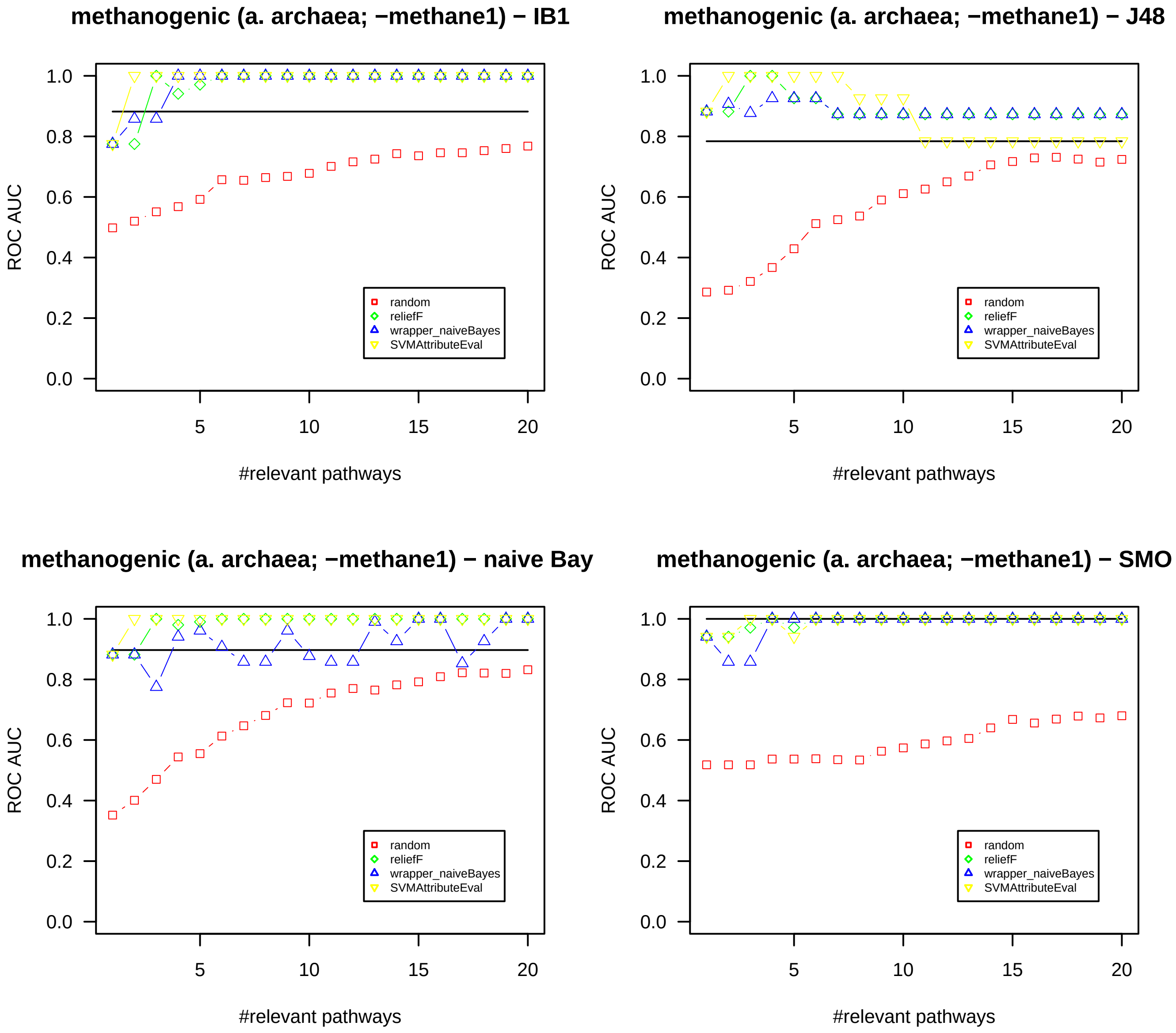

Figure S7: Cross-checking the Most Relevant Pathways for Methanogenesis among Archaea by Classification Disregarding the Methane Pathway and Using ROC AUC Values. The diagrams show the classification quality (assessed by the ROC AUC value) for the classification of completely sequenced archaea (23) into methanogens and non-methanogens. The classification is based on reduced pathway profiles containing only the 1 to 20 most relevant pathways (green: ReliefF, yellow: SVMAttributeEval, blue: wrapper (naive Bayes)), respectively. Thereby, attribute selection was performed on pathway profiles without the pathway for methane production. Thus, the most relevant pathways, on which classification is based, do not contain this pathway. The classification quality achieved for classification based on all (290) pathways is marked by a horizontal line (black). Red boxes depict the quality of classification based on randomly chosen 1 to 20 pathways (average quality for 25 times). For classification, we used the nearest neighbor classifier IB1, the decision tree classifier J48, the naive Bayes classifier, and the linear support vector machine SMO. The diagrams demonstrate that the identified most relevant pathways (listed in Table 1) are well suited to distinguish methanogenic and non-methanogenic archaea (max(ROC AUC) = 1.0), though the most characteristic metabolic pathway for methanogenesis (methane1) is not considered for classification.

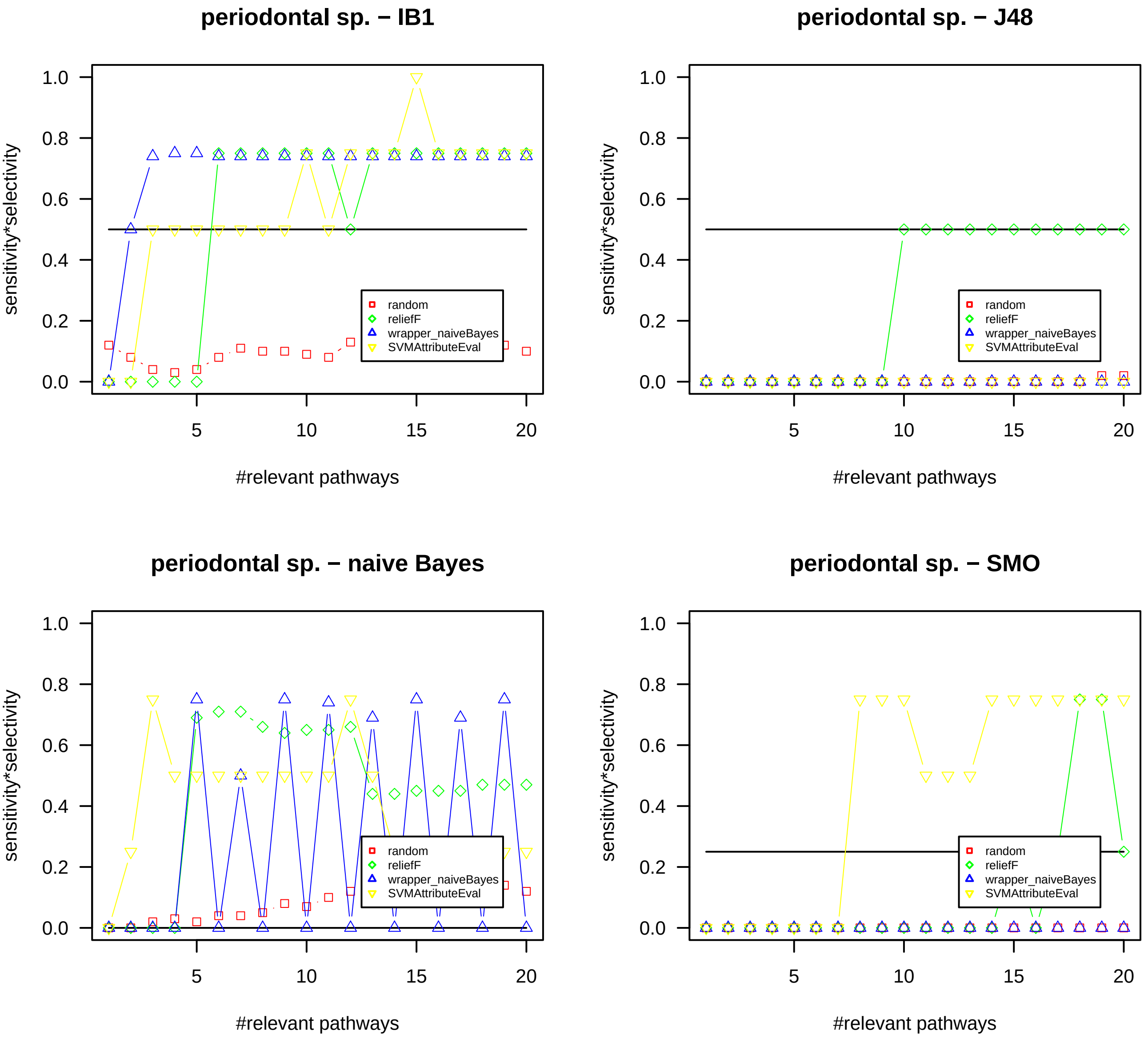

Figure S8: Cross-checking the Most Relevant Pathways for Periodontal Disease Causing Species by Classification. The diagrams show the classification quality (assessed by the product of sensitivity and selectivity) for the classification of the completely sequenced genomes (266) into species that are related and unrelated to periodontal disease. The classification is based on reduced pathway profiles containing only the 1 to 20 most relevant pathways (green: ReliefF, yellow: SVMAttributeEval, blue: wrapper (naive Bayes)), respectively. The classification quality achieved for classification based on all (290) pathways is marked by a horizontal line (black). Red boxes depict the quality of classification based on randomly chosen 1 to 20 pathways (average quality for 25 times). For classification, we applied the nearest neighbor classifier IB1, the decision tree classifier J48, the naive Bayes classifier, and the linear support vector machine SMO. The classification quality is remarkably enhanced when considering only the (up to) 10 most relevant pathways (listed in Table S2) compared to the quality achieved considering all or randomly picked pathways. Thus, the diagrams demonstrate that the identified most relevant pathways are distinctive for species related to periodontal disease. According to the cross-check, the most relevant pathways identified by attribute selection are considered as significant (see Methods).

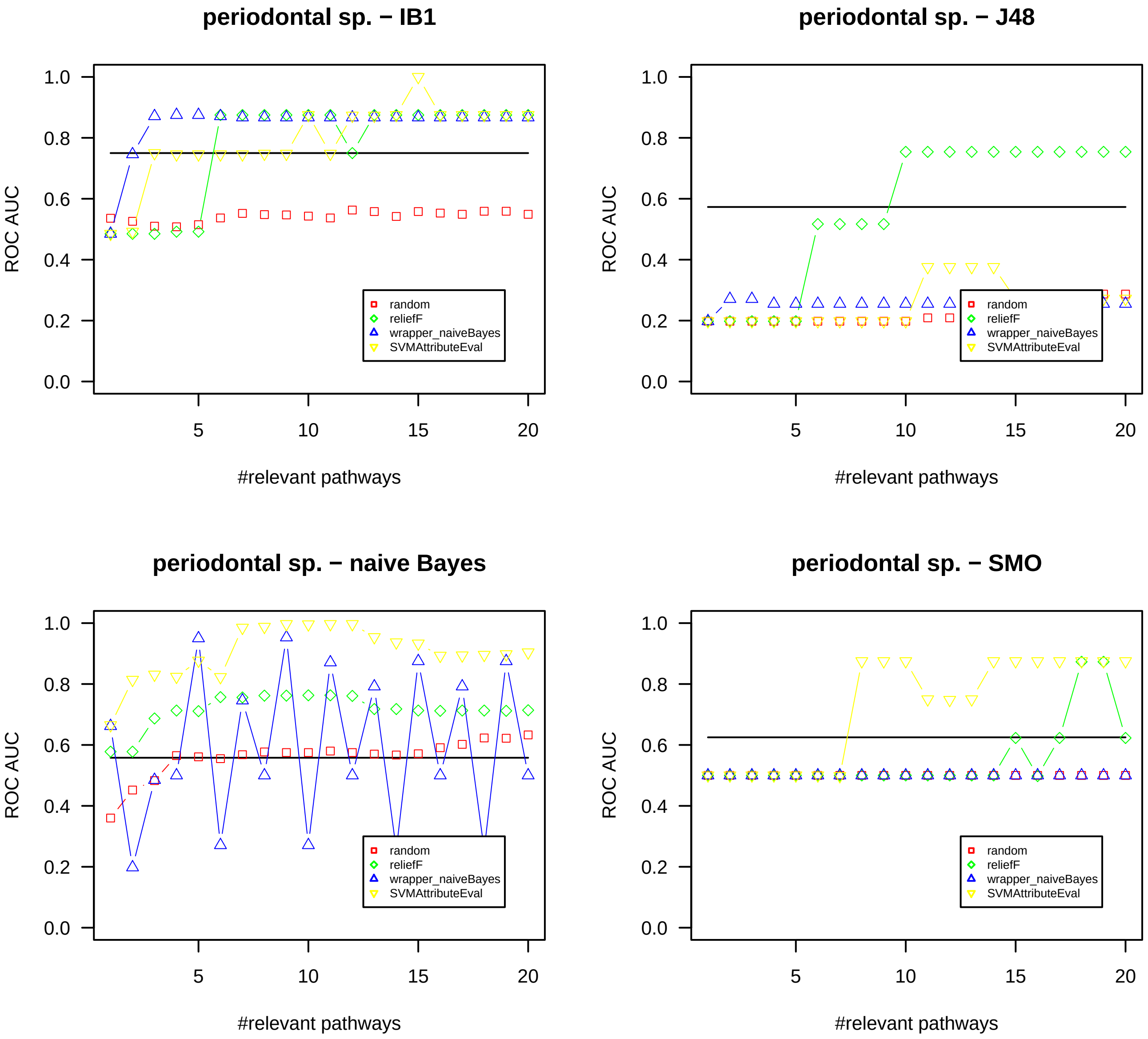

Figure S9: Cross-checking the Most Relevant Pathways for Periodontal Disease Causing Species by Classification Using ROC AUC Values. The diagrams show the classification quality (assessed by the ROC AUC value) for the classification of the completely sequenced genomes (266) into species that are related and unrelated to periodontal disease. The classification is based on reduced pathway profiles containing only the 1 to 20 most relevant pathways (green: ReliefF, yellow: SVMAttributeEval, blue: wrapper (naive Bayes)), respectively. The classification quality achieved for classification based on all (290) pathways is marked by a horizontal line (black). Red boxes depict the quality of classification based on randomly chosen 1 to 20 pathways (average quality for 25 times). For classification, we applied the nearest neighbor classifier IB1, the decision tree classifier J48, the naive Bayes classifier, and the linear support vector machine SMO. In contrast to the phenotype '*methanogenesis*', classification quality values are relatively low when considering all pathways (0.75, 0.57, 0.56, 0.63 for IB1, J48, naive Bayes, and SMO, respectively). Moreover, classification based on randomly picked pathways results in quality values that correspond to random classification (0.5) for 1 to 20 pathways. These results indicate that the over-all metabolism is not distinctive for the species related to periodontal disease. Considering only the most relevant pathways for classification remarkably improves the classification result.

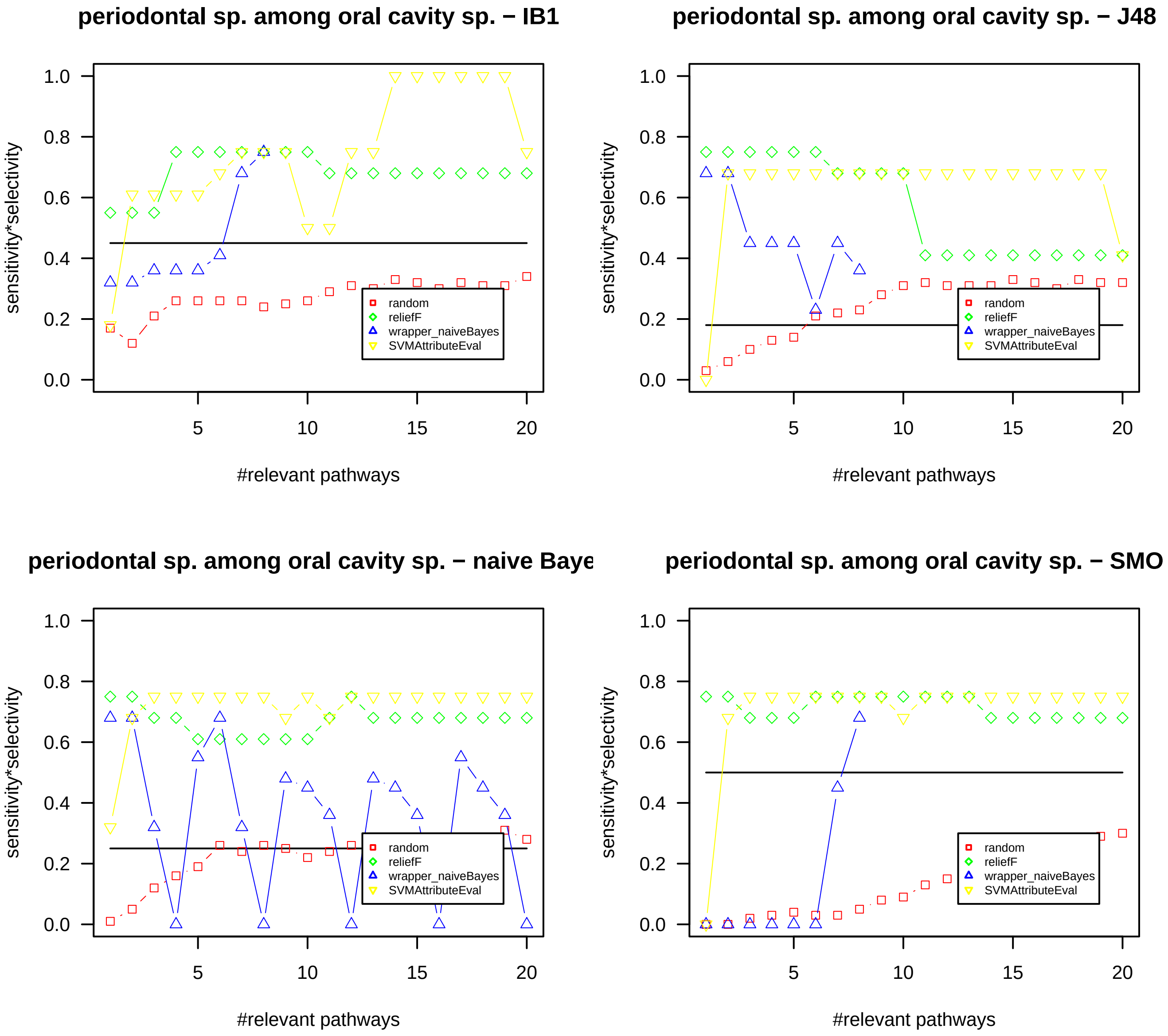

Figure S10: Cross-checking the Most Relevant Pathways for Periodontal Disease Causing among Oral Cavity Species by Classification. The diagrams show the classification quality (assessed by the product of sensitivity and selectivity) for the classification of oral cavity species (15) into those that are related and those that are unrelated to periodontal disease. The classification is based on reduced pathway profiles containing only the 1 to 20 most relevant pathways (green: ReliefF, yellow: SVMAttributeEval, blue: wrapper (naive Bayes)), respectively. The classification quality achieved for classification based on all (290) pathways is marked by a horizontal line (black). Red boxes depict the quality of classification based on randomly chosen 1 to 20 pathways (average quality for 25 times). For classification, we applied the nearest neighbor classifier IB1, the decision tree classifier J48, the naive Bayes classifier, and the linear support vector machine SMO. The classification quality is remarkably enhanced when considering only the (up to) 10 most relevant pathways (listed in Table S2) compared to the quality achieved considering all or randomly picked pathways. Thus, the diagrams demonstrate that the identified most relevant pathways are distinctive for species related to periodontal disease among the species residing in the oral cavity. According to the cross-check, the most relevant pathways identified by attribute selection are considered as significant (see Methods).

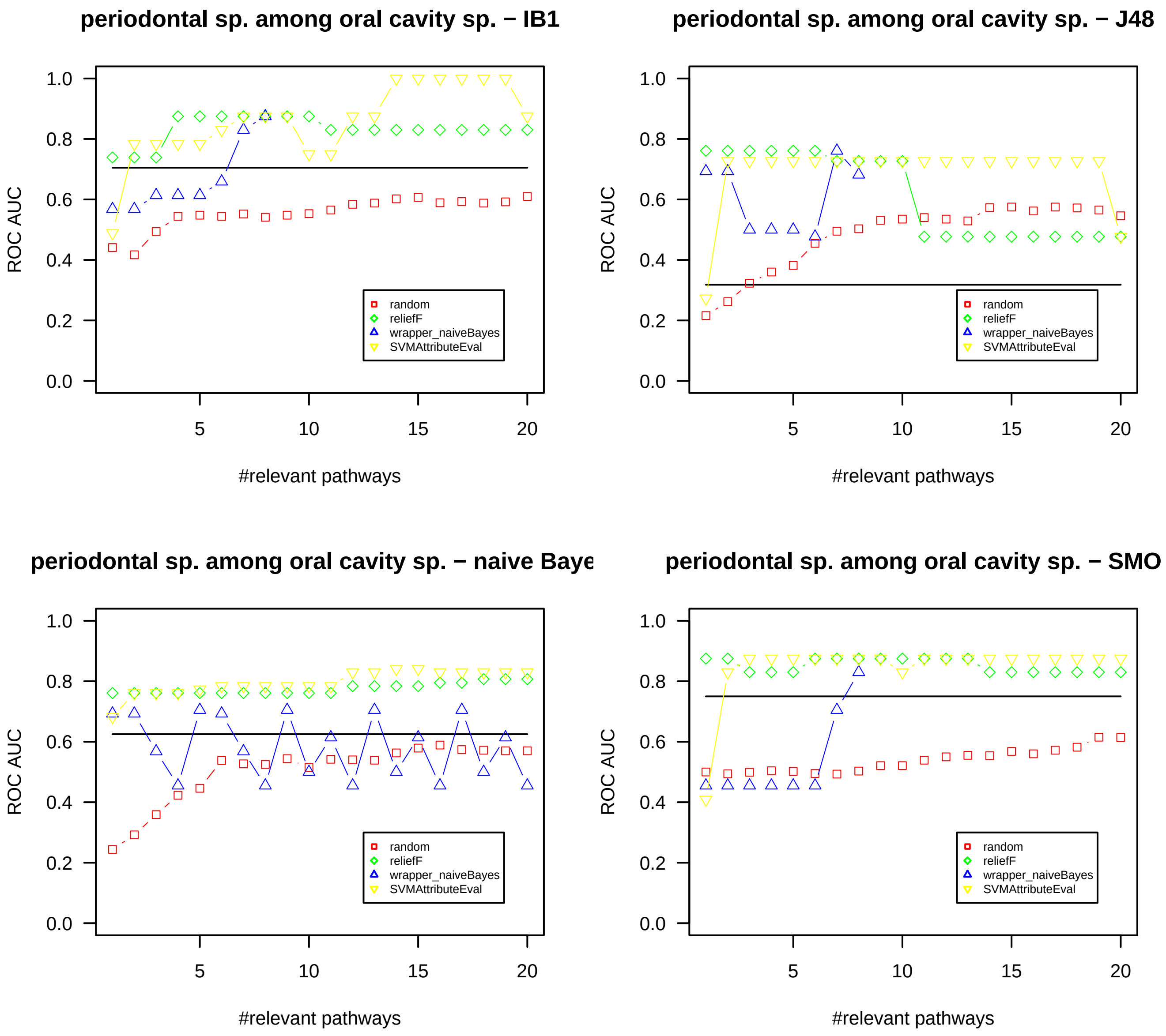

Figure S11: Cross-checking the Most Relevant Pathways for Periodontal Disease Causing among Oral Cavity Species by Classification Using ROC AUC Values. The diagrams show the classification quality (assessed by the ROC AUC value) for the classification of oral cavity species (15) into species that are related and unrelated to periodontal disease. The classification is based on reduced pathway profiles containing only the 1 to 20 most relevant pathways (green: ReliefF, yellow: SVMAttributeEval, blue: wrapper (naive Bayes)), respectively. The classification quality achieved for classification based on all (290) pathways is marked by a horizontal line (black). Red boxes depict the quality of classification based on randomly chosen 1 to 20 pathways (average quality for 25 times). For classification, we applied the nearest neighbor classifier IB1, the decision tree classifier J48, the naive Bayes classifier, and the linear support vector machine SMO. The diagrams demonstrate that the identified most relevant pathways are well suited to distinguish periodontal and non-periodontal oral species. The diagram demonstrates that considering only the most relevant pathways for the classification of periodontal and non-periodontal oral species remarkably improves the results.

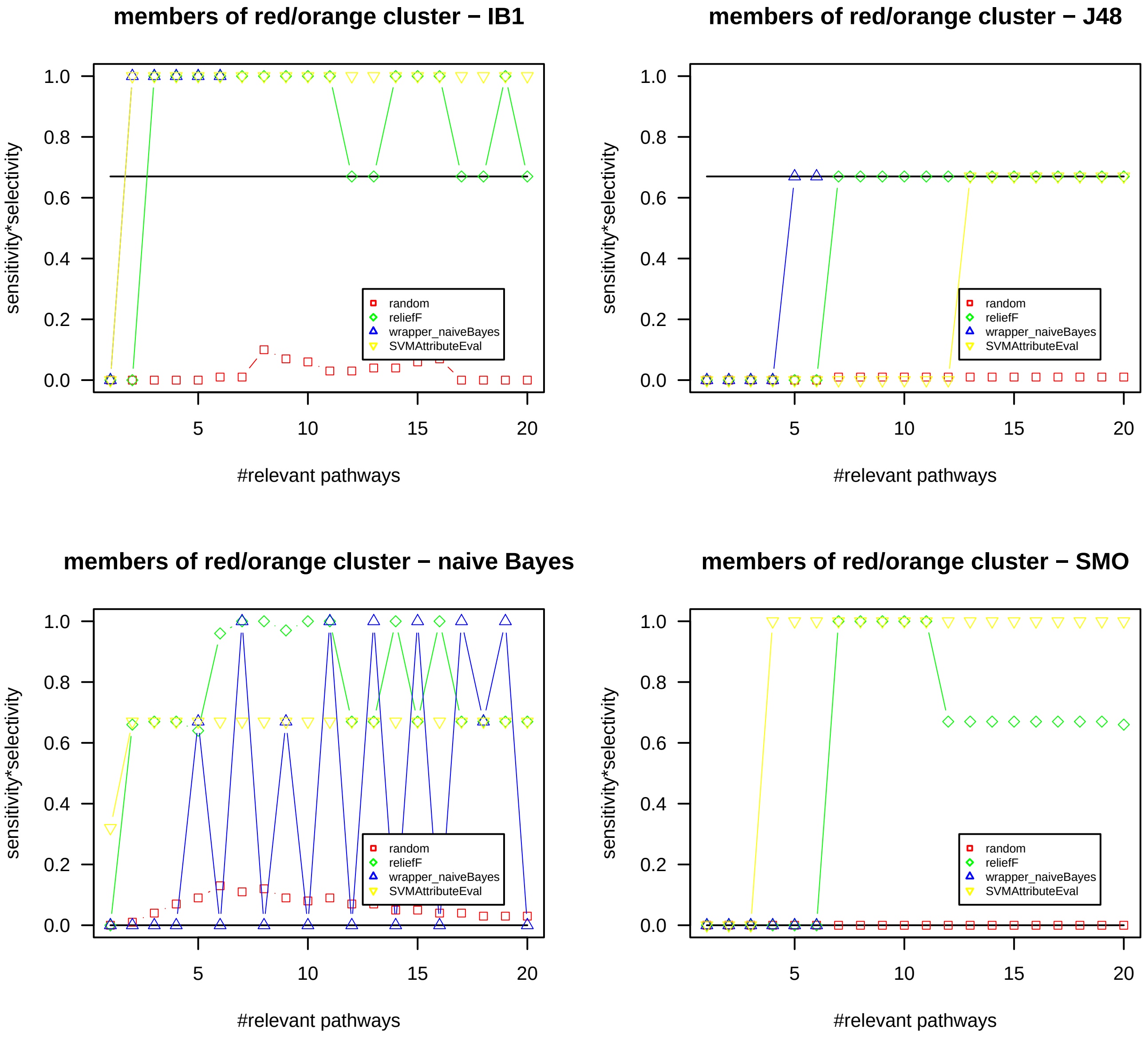

Figure S12: Cross-checking the Most Relevant Pathways for Species Contained in the Red/Orange Cluster by Classification. The diagrams show the classification quality (assessed by the product of sensitivity and selectivity) for the classification of the completely sequenced genomes (266) into species that are contained in the so-called red or orange clusters [42] and those that are not contained in these clusters. The classification is based on reduced pathway profiles containing only the 1 to 20 most relevant pathways (green: ReliefF, yellow: SVMAttributeEval, blue: wrapper (naive Bayes)), respectively. The classification quality achieved for classification based on all (290) pathways is marked by a horizontal line (black). Red boxes depict the quality of classification based on randomly chosen 1 to 20 pathways (average quality for 25 times). For classification, we applied the nearest neighbor classifier IB1, the decision tree classifier J48, the naive Bayes classifier, and the linear support vector machine SMO. The classification quality is remarkably enhanced when considering only the (up to) 10 most relevant pathways (listed in Table S2) compared to the quality achieved considering all or randomly picked pathways. Thus, the diagrams demonstrate that the identified most relevant pathways are distinctive for species which are members of the clusters (max(sensitivity*selectivity) = 1.0). According to the cross-check, the most relevant pathways identified by attribute selection are considered as significant (see Methods).

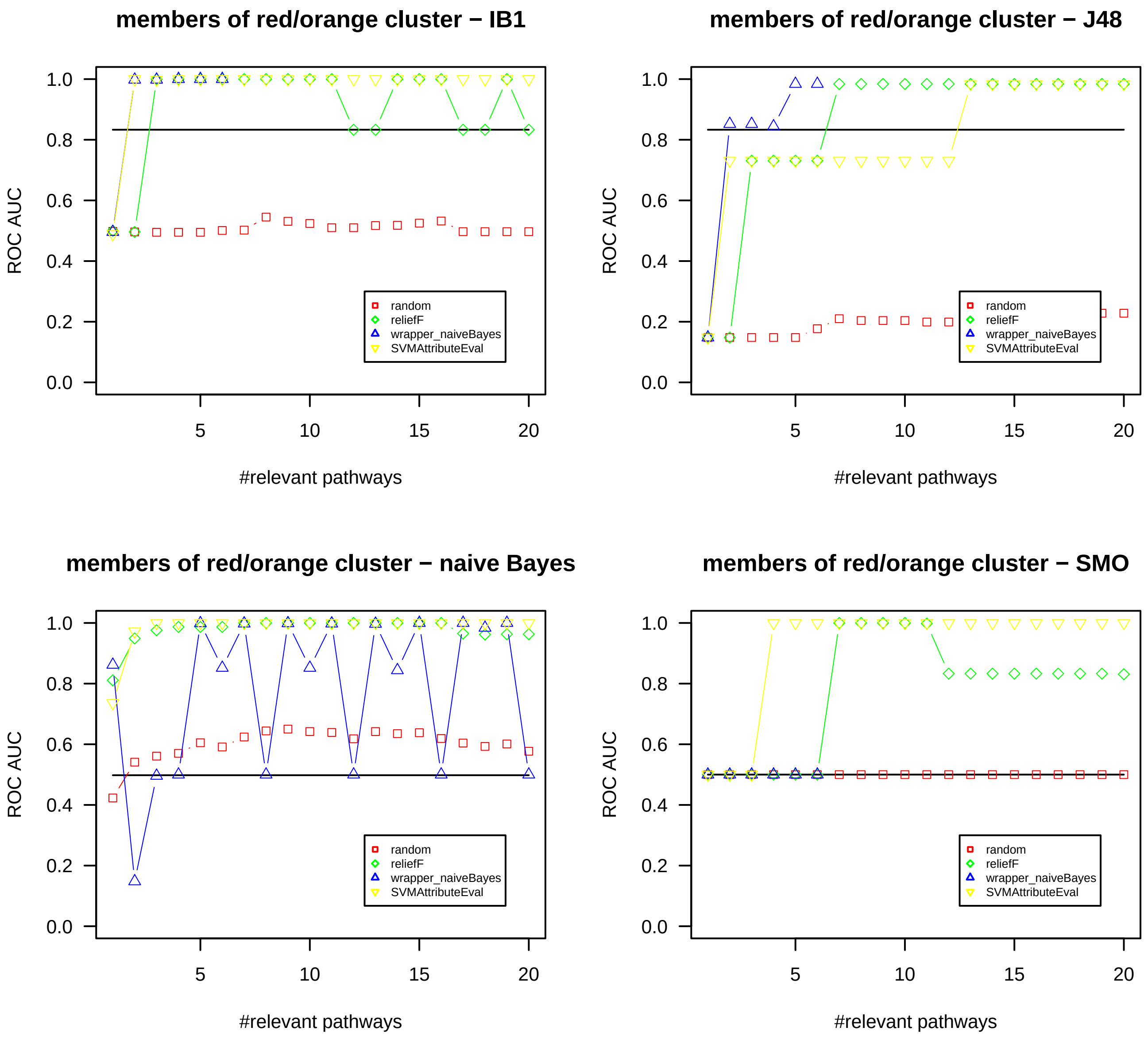

Figure S13: Cross-checking the Most Relevant Pathways for Species Contained in the Red/Orange Cluster by Classification Using ROC AUC Values. The diagrams show the classification quality (assessed by the ROC AUC value) for the classification of the completely sequenced genomes (266) into species that are contained in the so-called red or orange clusters [42] and those that are not contained in these clusters. The classification is based on reduced pathway profiles containing only the 1 to 20 most relevant pathways (green: ReliefF, yellow: SVMAttributeEval, blue: wrapper (naive Bayes)), respectively. The classification quality achieved for classification based on all (290) pathways is marked by a horizontal line (black). Red boxes depict the quality of classification based on randomly chosen 1 to 20 pathways (average quality for 25 times). For classification, we applied the nearest neighbor classifier IB1, the decision tree classifier J48, the naive Bayes classifier, and the linear support vector machine SMO. The diagrams demonstrate that the identified most relevant pathways are well suited to distinguish species that are contained in the red/orange clusters from those that are not (max(ROC AUC) = 1.0). Classification based on randomly picked pathways results in quality values that correspond to random classification (0.5) for 1 to 20 pathways. Furthermore, consideration of all pathways also provides ROC AUC values around 0.5 for the classification using naive Bayes and SMO. These results indicate that the over-all metabolism is not distinctive for the species that are members of the red/orange clusters.

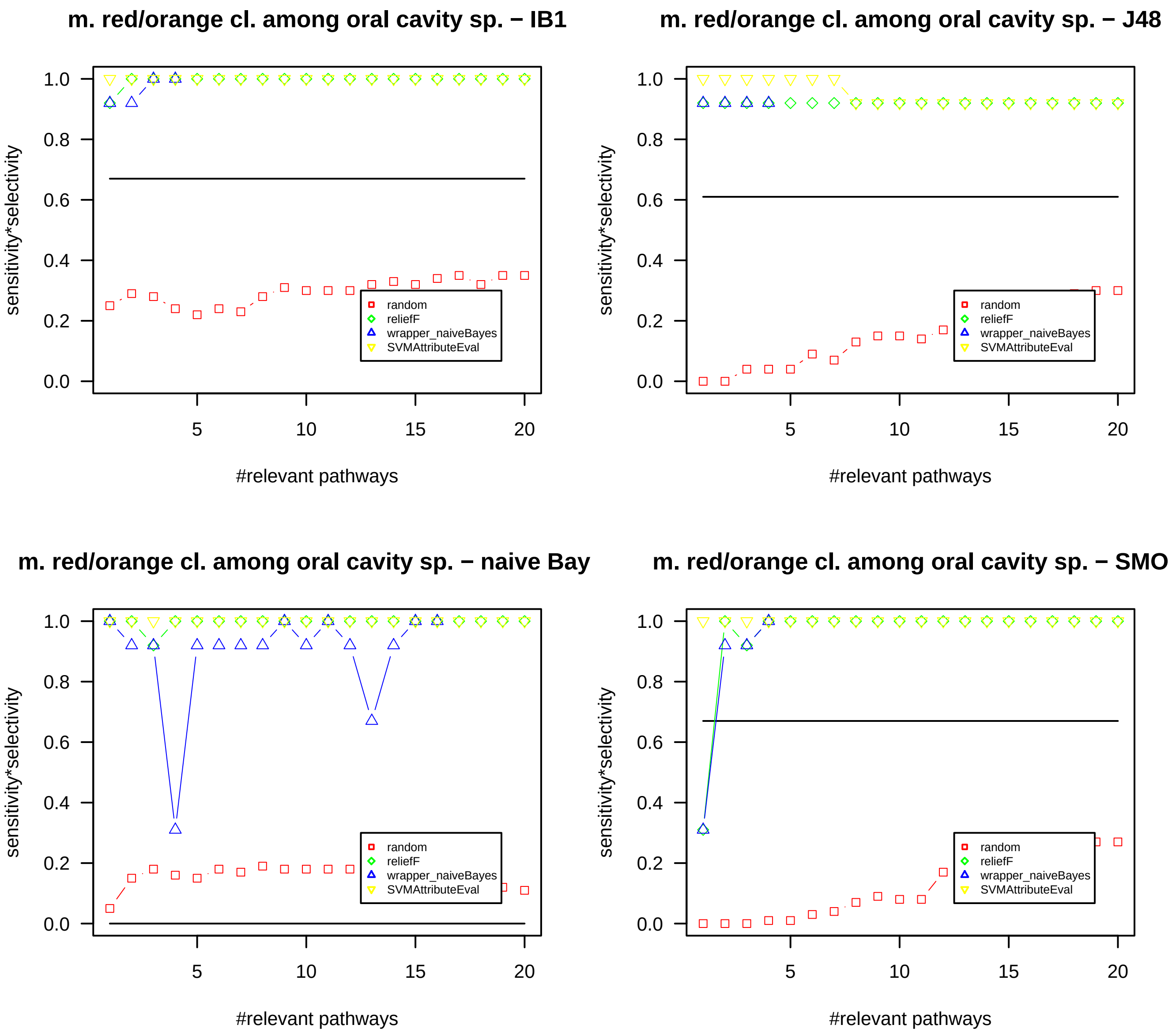

Figure S14: Cross-checking the Most Relevant Pathways for Species Contained in the Red/Orange Cluster among Oral Cavity Species by Classification. The diagrams show the classification quality (assessed by the product of sensitivity and selectivity) for the classification of oral cavity species (15) into those that are contained in the so-called red or orange clusters [42] and those that are not contained in these clusters. The classification is based on reduced pathway profiles containing only the 1 to 20 most relevant pathways (green: ReliefF, yellow: SVMAttributeEval, blue: wrapper (naive Bayes)), respectively. The classification quality achieved for classification based on all (290) pathways is marked by a horizontal line (black). Red boxes depict the quality of classification based on randomly chosen 1 to 20 pathways (average quality for 25 times). For classification, we applied the nearest neighbor classifier IB1, the decision tree classifier J48, the naive Bayes classifier, and the linear support vector machine SMO. The classification quality is remarkably enhanced when considering only the (up to) 10 most relevant pathways (listed in Table S2) compared to the quality achieved considering all or randomly picked pathways. Thus, the diagrams demonstrate that the identified most relevant pathways are distinctive for species which are members of the clusters (max(sensitivity*selectivity) = 1.0). According to the cross-check, the most relevant pathways identified by attribute selection are considered as significant (see Methods).

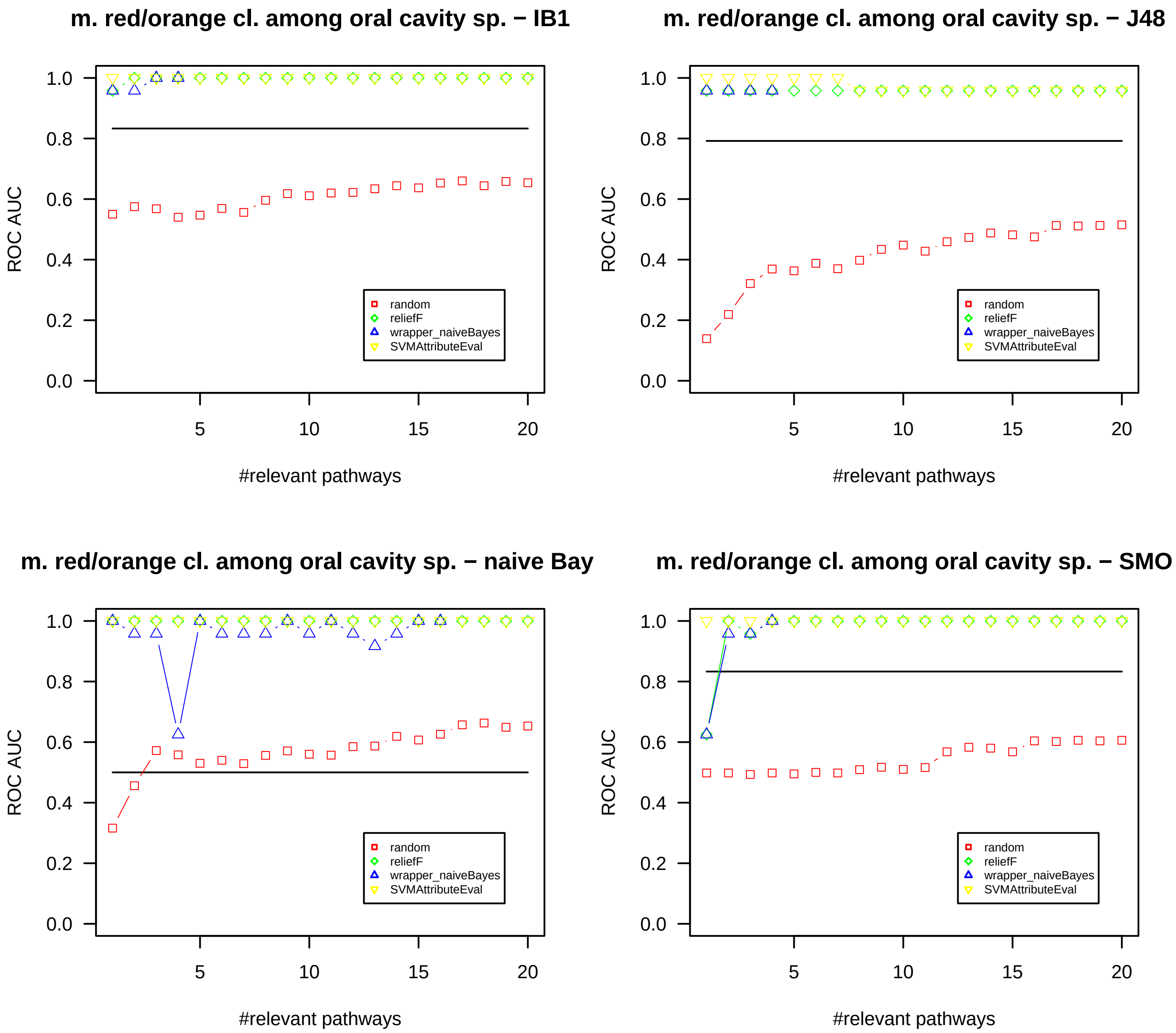

Figure S15: Cross-checking the Most Relevant Pathways for Species Contained in the Red/Orange Cluster among Oral Cavity Species by Classification Using ROC AUC Values. The diagrams show the classification quality (assessed by the ROC AUC value) for the classification of oral cavity species (15) into those that are contained in the so-called red or orange clusters [42] and those that are not contained in these clusters. The classification is based on reduced pathway profiles containing only the 1 to 20 most relevant pathways (green: ReliefF, yellow: SVMAttributeEval, blue: wrapper (naive Bayes)), respectively. The classification quality achieved for classification based on all (290) pathways is marked by a horizontal line (black). Red boxes depict the quality of classification based on randomly chosen 1 to 20 pathways (average quality for 25 times). For classification, we applied the nearest neighbor classifier IB1, the decision tree classifier J48, the naive Bayes classifier, and the linear support vector machine SMO. The diagrams demonstrate that the identified most relevant pathways are well suited to distinguish species that are contained in the red/orange clusters from those that are not (max(ROC AUC) = 1.0).

Table S2: Relevant Pathways for Periodontal Disease

dataset	ReliefF	SVMAttributeEval	wrapper (naive Bayes)
periodontal species among all genomes (266)	Biosynth. of coproporphyrin I (pyrrole6)<br>Biosynth. of coproporphyrin III (pyrrole8)<br>Biosynth. of L-proline (proline1)<br>Urea cycle (part) (urea2)<br>Conversion of L-glutamate to L-proline (glutamate3)<br>Biosynth. of 5-formimino-THF (c2)<br>Conversion of L-glutamate to L-ornithine (glutamate2)<br>Glyoxylate cycle (part) (gc1)<br>TCA cycle (part) (cc2)<br>Glutamate fermentation (fnc1)	Biosynth. of GTP (gn1)<br>Glycolysis and Gluconeogenesis (part) (gg13)<br>Biosynth. of 5-formimino-THF (c2)<br>Biosynth. of 5-aminolevulinate from succinate (pyrrole10)<br>Glycolate/glyoxylate interconversion (sg6)<br>Biosynth. of L-serine (sg1)<br>Glyoxylate cycle (part) (gc1)<br>Degrad. of L-glutamate (glutamate6)<br>Conversion of NAD+ To NADP+ (nad3)<br>Biosynth. of amylopectin (starch1)	Biosynth. of coenzyme B12 (coba1)<br>TCA cycle (part) (cc2)<br>Degrad. of histidine to imidazoleacetate (histidine5)<br>Biosynth. of L-proline (proline1)<br>Phosphorylation of arginine (arginine3)<br>Biosynth. of GTP (gn1)<br>Alcoholic fermentation of pyruvate (aar4)
periodontal species among oral cavity sp. (15)	Biosynth. of 5-formimino-THF (c2)<br>Degrad. of L-histidine to L-glutamate (histidine2)<br>Biosynth. of coenzyme B12 (coba1)<br>Biosynth. of histidine (histidine1)<br>Conversion of L-glutamate to L-proline (glutamate3)<br>Biosynth. of L-proline (proline1)<br>Glutamate fermentation (fnc1)<br>Glycolysis and Gluconeogenesis (part) (gg13)<br>Urea cycle (part) (urea2)<br>Conversion of L-glutamate to L-ornithine (glutamate2)	Glycolysis and Gluconeogenesis (part) (gg13)<br>Degrad. of L-histidine to L-glutamate (histidine2)<br>Biosynth. of 5-formimino-THF (c2)<br>Glutamate fermentation (fnc1)<br>Biosynth. of 5,10-methylene-THF (c3)<br>Biosynth. of L-leucine (vas3)<br>Odd-numbered fatty acid metabolism (glf2)<br>Biosynth. of coenzyme B12 (coba1)<br>Conversion of NAD+ To NADP+ (nad3)<br>Biosynth. of 5-aminolevulinate from succinate (pyrrole10)	Biosynth. of L-proline (proline1)<br>Degrad. of L-cysteine to taurine (cysteine4)<br>Biosynth. of 2'-deoxyuridine-5'-phosphate (dun2)<br>Degrad. of creatine (creatine2)<br>Biosynth. of giberellin GA3 (isoprenoids9)<br>Biosynth. of ornithine (urea5)<br>omega-Oxidation of alkanes (fa3)<br>Biosynth. of 5,10-methylene-THF (c3)
members of red/orange cluster among all genomes (266)	Urea cycle (part) (urea2)<br>Biosynth. of 5-formimino-THF (c2)<br>Conversion of L-glutamate to L-proline (glutamate3)<br>Biosynth. of coproporphyrin I (pyrrole6)<br>Biosynth. of L-proline (proline1)<br>Biosynth. of coproporphyrin III (pyrrole8)<br>Glutamate fermentation (fnc1)<br>Biosynth. of GTP (gn1)<br>Conversion of L-glutamate to L-ornithine (glutamate2)<br>Biosynth. of coenzyme B12 (coba1)	Biosynth. of 5-formimino-THF (c2)<br>Conversion of L-glutamate to L-proline (glutamate3)<br>Biosynth. of coenzyme B12 (coba1)<br>Urea cycle (part) (urea2)<br>Biosynth. of L-proline (proline1)<br>Glycolate/glyoxylate interconversion (sg6)<br>Biosynth. of estriol (steroidhormones10)<br>Biosynth. of GTP (gn1)<br>Degrad. of L-histidine to L-glutamate (histidine2)<br>Degrad. of sucrose (sucrose2)	Biosynth. of coenzyme B12 (coba1)<br>Biosynth. of L-proline (proline1)<br>Degrad. of histidine to imidazoleacetate (histidine5)<br>Glutamate fermentation (fnc1)<br>Biosynth. of IMP (in1)<br>Alcoholic fermentation of pyruvate (aar4)
members of red/orange cluster among oral cavity sp. (15)	Degrad. of L-histidine to L-glutamate (histidine2)<br>Biosynth. of 5-formimino-THF (c2)<br>Biosynth. of coenzyme B12 (coba1)<br>Biosynth. of L-proline (proline1)<br>Conversion of L-glutamate to L-proline (glutamate3)<br>Urea cycle (part) (urea2)<br>Glutamate fermentation (fnc1)<br>Conversion of L-glutamate to L-ornithine (glutamate2)<br>Biosynth. of dATP from ADP (dan1)<br>Biosynth. of dGTP from GDP (dgn1)	Biosynth. of 5-formimino-THF (c2)<br>Degrad. of L-histidine to L-glutamate (histidine2)<br>Degrad. of sucrose (sucrose2)<br>Biosynth. of coenzyme B12 (coba1)<br>Glycolysis and Gluconeogenesis (part) (gg13)<br>Urea cycle (part) (urea2)<br>Conversion of L-glutamate to L-proline (glutamate3)<br>Glutamate fermentation (fnc1)<br>Biosynth. of L-proline (proline1)<br>Conversion of L-glutamate to L-ornithine (glutamate2)	Degrad. of L-histidine to L-glutamate (histidine2)<br>Biosynth. of coenzyme B12 (coba1)<br>Biosynth. of L-proline (proline1)<br>Biosynth. of 2'-deoxyuridine-5'-phosphate (dun2)

The relevant pathways for the phenotypes '*periodontal disease causing*' and '*member of the red/orange cluster*' were determined by applying three different attribute selection methods (ReliefF, SVMAttributeEval, and a wrapper for the naive Bayes classifier) to two different datasets. The (up to) ten most relevant pathways received for the complete set of pathway profiles (266 genomes), and the set of pathway profiles (15 genomes) for the species residing in the oral cavity are shown here.

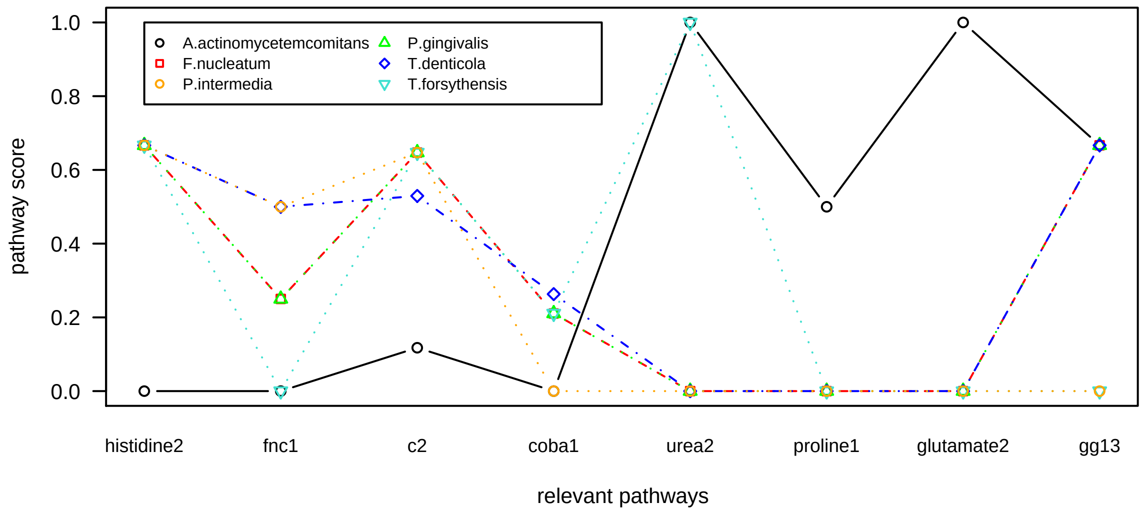

Figure S16: Pathway Scores of the Relevant Pathways for *F.nucleatum*, *P.gingivalis*, and *T.denticola* in comparison to the scores for *T.forsythia* and *P.intermedia*. This figure shows the diagram of Figure 7 with the pathway scores for *T.forsythia* (turquoise) and *P.intermedia* (orange) added. The scores obtained are largely similar to those obtained for *F.nucleatum* (red), *P.gingivalis* (green), and *T.denticola* (blue).

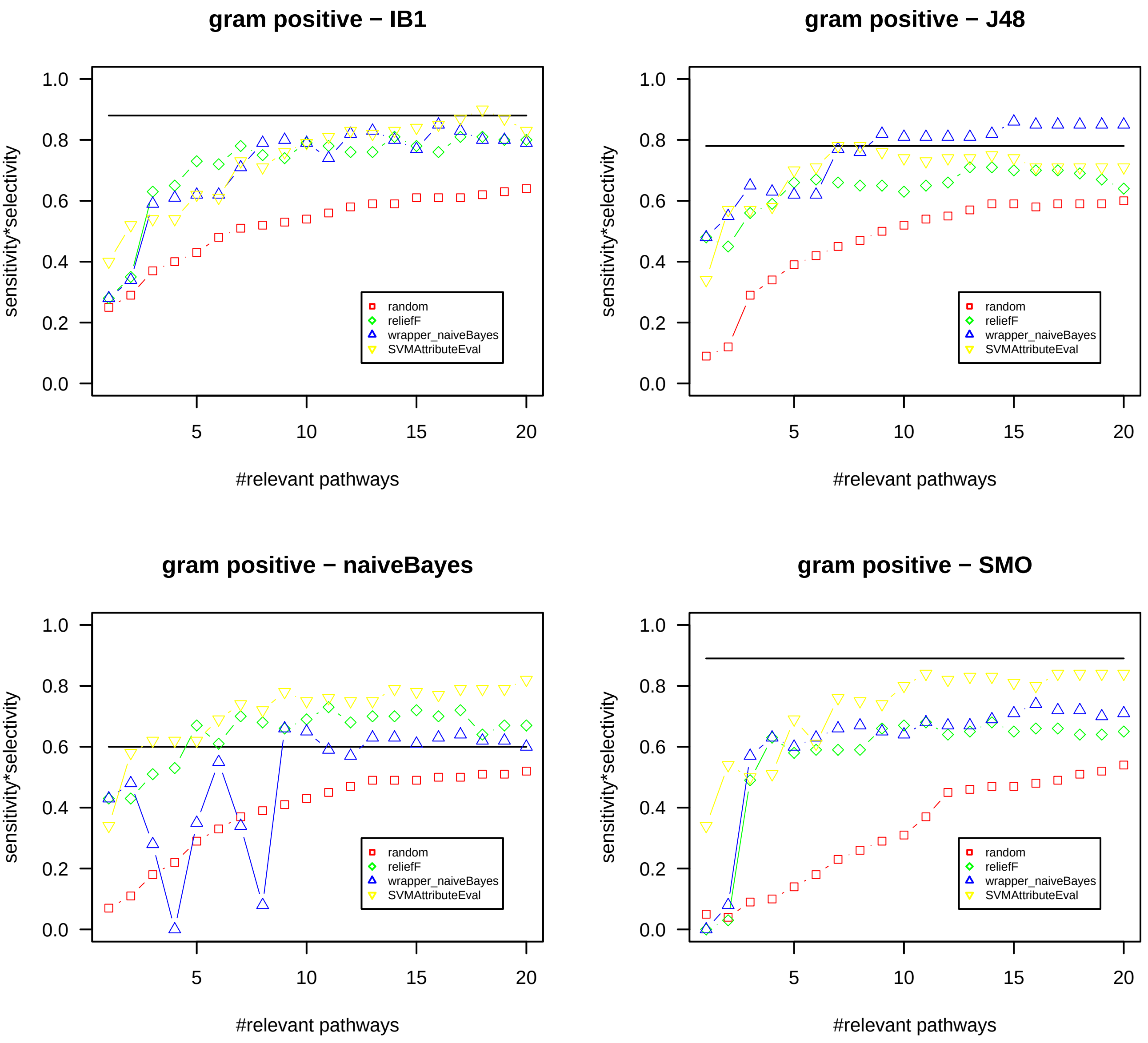

Figure S17: Cross-checking the Most Relevant Pathways for Gram-positivity by Classification. The diagrams show the classification quality (assessed by the product of sensitivity and selectivity) for the classification of the completely sequenced genomes (266) into gram-positive and gram-negative species. The classification is based on reduced pathway profiles containing only the 1 to 20 most relevant pathways (green: ReliefF, yellow: SVMAttributeEval, blue: wrapper (naive Bayes)), respectively. The classification quality achieved for classification based on all (290) pathways is marked by a horizontal line (black). Red boxes depict the quality of classification based on randomly chosen 1 to 20 pathways (average quality for 25 times). For classification, we applied the nearest neighbor classifier IB1, the decision tree classifier J48, the naive Bayes classifier, and the linear support vector machine SMO. The classification quality is remarkably enhanced when considering only the (up to) 10 most relevant pathways (listed in Table S3) compared to the quality achieved considering all or randomly picked pathways. Thus, the diagrams demonstrate that the identified most relevant pathways are distinctive for gram-positive species.

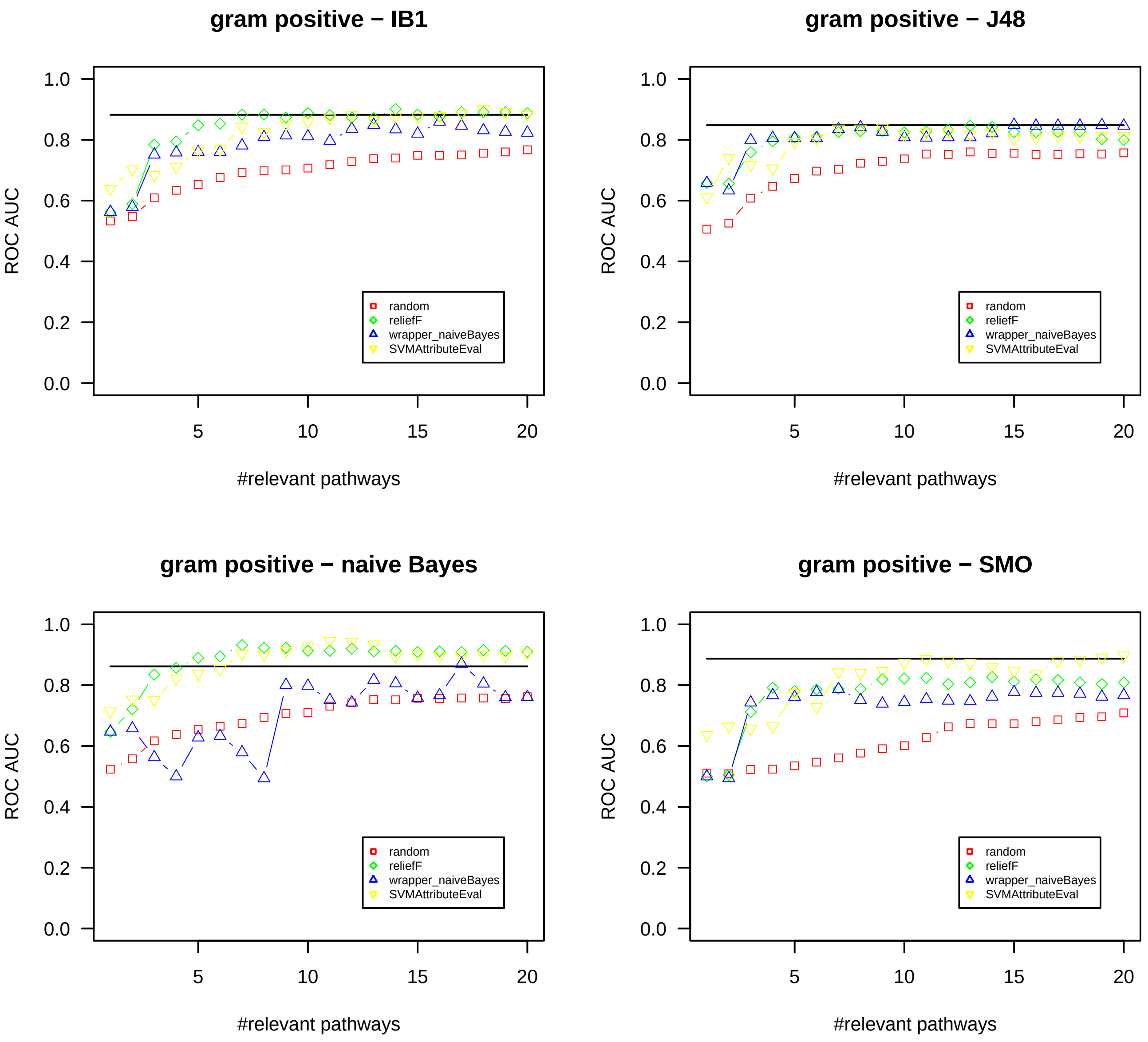

Figure S18: Cross-checking the Most Relevant Pathways for Gram-positivity by Classification Using ROC AUC Values. The diagrams (266) show the classification quality (assessed by the ROC AUC value) for the classification of the completely sequenced genomes into gram-positive and gram-negative species. The classification is based on reduced pathway profiles containing only the 1 to 20 most relevant pathways (green: ReliefF, yellow: SVMAttributeEval, blue: wrapper (naive Bayes)), respectively. The classification quality achieved for classification based on all (290) pathways is marked by a horizontal line (black). Red boxes depict the quality of classification based on randomly chosen 1 to 20 pathways (average quality for 25 times). For classification, we used the nearest neighbor classifier IB1, the decision tree classifier J48, the naive Bayes classifier, and the linear support vector machine SMO. The diagrams demonstrate that the identified most relevant pathways are well suited to distinguish gram-positive and gram-negative species (max(ROC AUC) = 0.93). The increase in classification quality for the rising number of randomly picked pathways indicates that the over-all metabolism is similar within the group of gram-positive species and distinctive when compared to the group of gram-negative species.

Table S3: Relevant Pathways for Gram-positivity

method	relevant pathways
ReliefF	Biosynthesis of phosphatidylserine (phospholipids3)<br>Glutathion metabolism (glutamate5)<br>Degradation of arginine (arginine1)<br>Degradation of sucrose (sucrose2)<br>Glycolysis and Gluconeogenesis (part) (gg12)<br>Biosynthesis of menaquinone (aaa2)<br>Biosynthesis of 5-aminolevulinate from succinate (pyrrole10)<br>TCA cycle (part) (cc4)<br>Pyruvate fermentation (gg17)<br>Biosynthesis of triacylglycerols (triacylglycerols1)
SVMAttributeEval	Degradation of arginine (arginine1)<br>Biosynthesis of limonene (isoprenoids3)<br>Degradation of 4-hydroxyproline (hydroxyprol3)<br>Degradation of L-aspartate (aspartate2)<br>Synthesis of serine and glycine (sg5)<br>Glycolysis and Gluconeogenesis (part) (gg12)<br>Biosynthesis of 5-aminolevulinate from succinate (pyrrole10)<br>Biosynthesis of coenzyme A (coa1)<br>Conversion of NAD+ To NADP+ (nad3)<br>Biosynthesis of triacylglycerols (triacylglycerols1)
wrapper (naive Bayes)	Biosynthesis of phosphatidylserine (phospholipids3)<br>Degradation of 4-hydroxyproline (hydroxyprol3)<br>Degradation of arginine (arginine1)<br>Synthesis of serine and glycine (sg5)<br>Degradation of L-cysteine to taurine (cysteine4)<br>Degradation of 2'-deoxyadenosine to adenine (dan4)<br>Alcoholic fermentation (glf5)<br>Biosynthesis of dolichol (isoprenoids1)<br>Biosynthesis of alpha-pinene (isoprenoids2)<br>Biosynthesis of spermine from arginine (polyamine4)

The relevant pathways for gram positivity were determined by applying three different attribute selection methods (ReliefF, SVMAttributeEval, and a wrapper for the naive Bayes classifier) to the complete set of pathway profiles (266 genomes). Here, the ten most relevant pathways identified by the different attributes selection methods are listed, respectively.

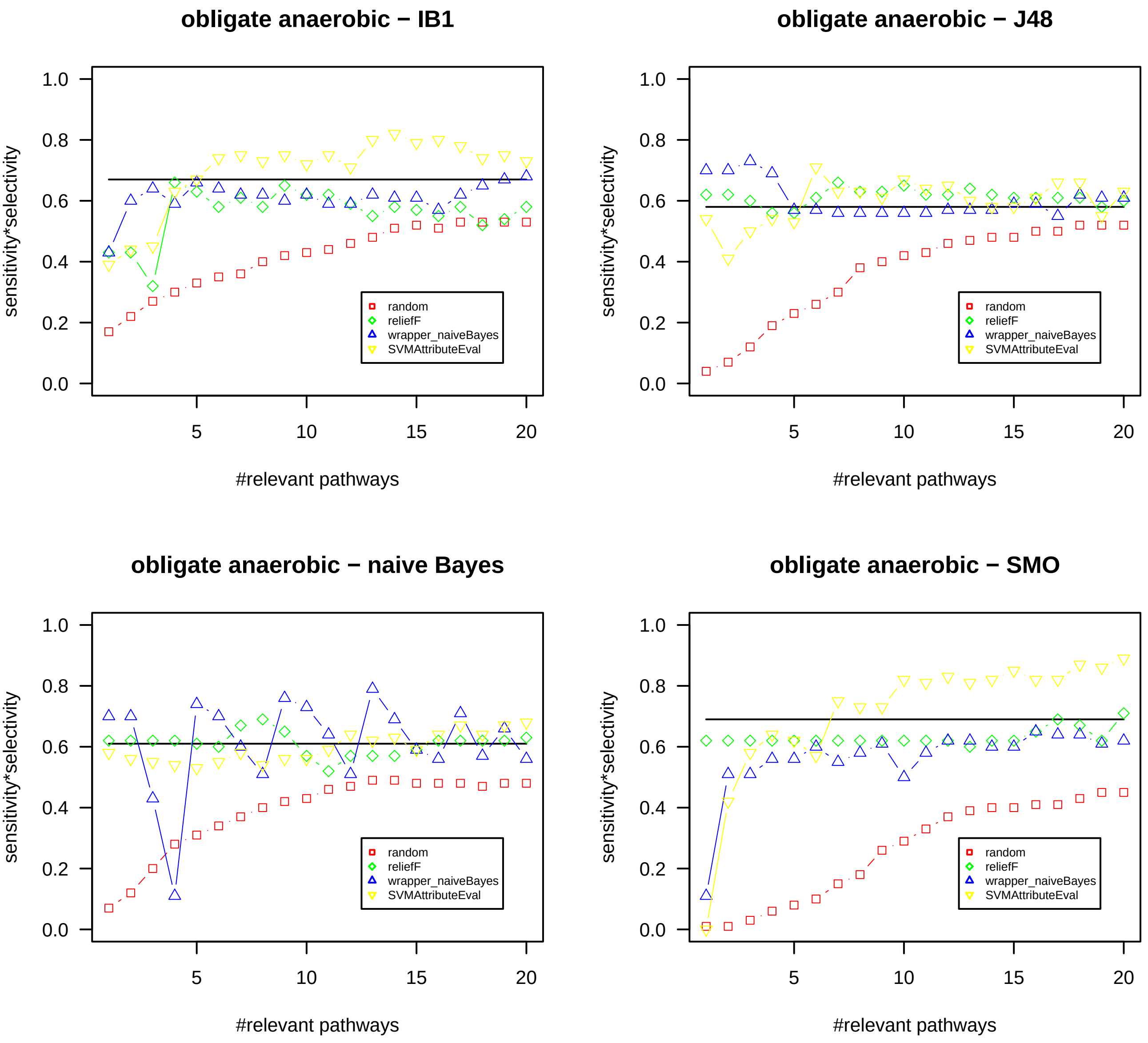

Figure S19: Cross-checking the Most Relevant Pathways for Anaerobicity by Classification. The diagrams show the classification quality (assessed by the product of sensitivity and selectivity) for the classification of the completely sequenced (obligate) aerobe and (obligate) anaerobe genomes (113) into these two groups. The classification is based on reduced pathway profiles containing only the 1 to 20 most relevant pathways (green: ReliefF, yellow: SVMAttributeEval, blue: wrapper (naive Bayes)), respectively. The classification quality achieved for classification based on all (290) pathways is marked by a horizontal line (black). Red boxes depict the quality of classification based on randomly chosen 1 to 20 pathways (average quality for 25 times). For classification, we applied the nearest neighbor classifier IB1, the decision tree classifier J48, the naive Bayes classifier, and the linear support vector machine SMO. The classification quality is remarkably enhanced when considering only the (up to) 5 most relevant pathways (listed in Table S4) compared to the quality achieved considering all or randomly picked pathways. Thus, the diagrams demonstrate that the identified most relevant pathways are distinctive for (obligate) anaerobe species.

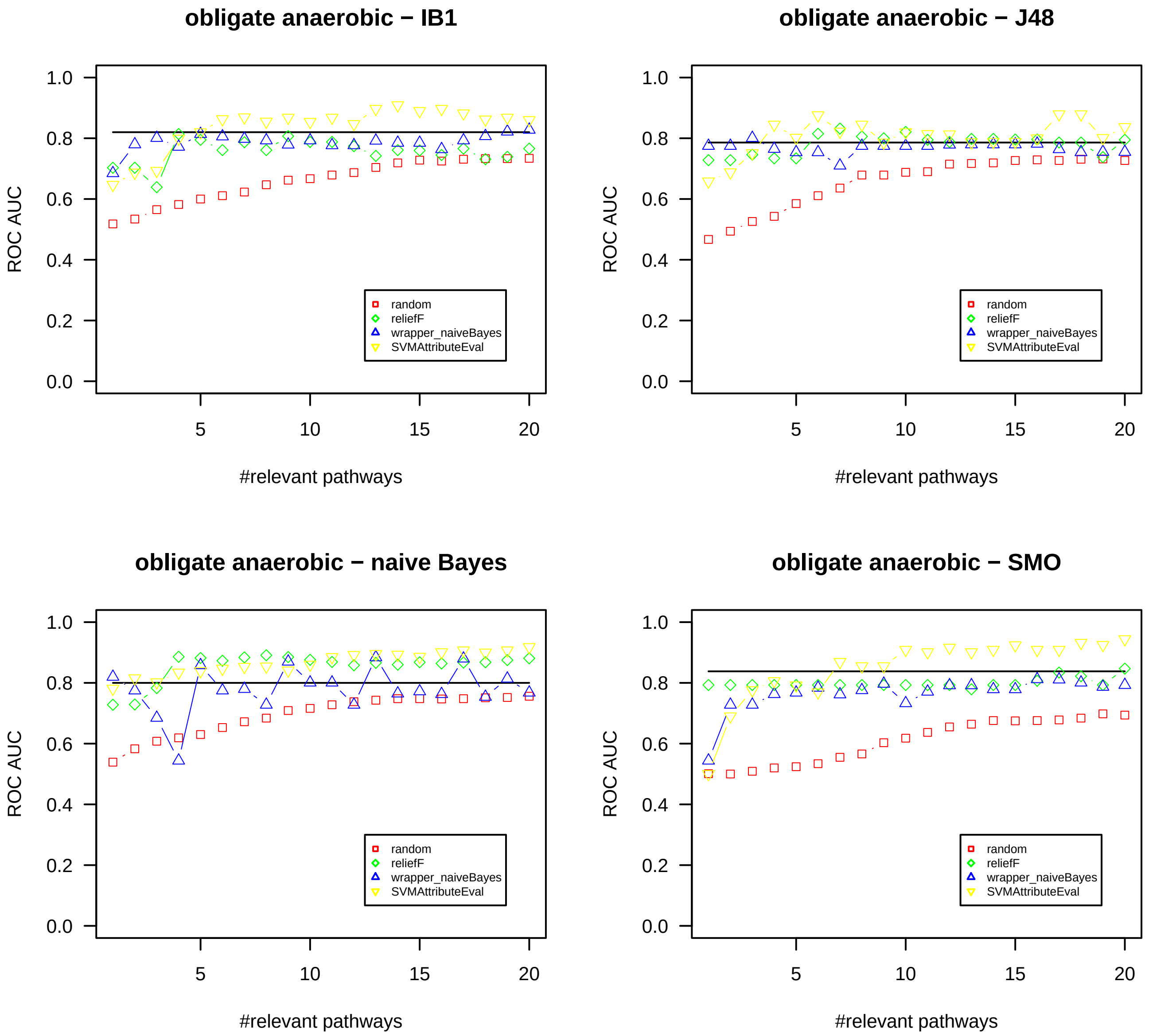

Figure S20: Cross-checking the Most Relevant Pathways for Anaerobicity by Classification Using ROC AUC Values. The diagrams show the classification quality (assessed by the ROC AUC value) for the classification of the completely sequenced (obligate) aerobe and (obligate) anaerobe genomes (113) into these two groups. The classification is based on reduced pathway profiles containing only the 1 to 20 most relevant pathways (green: ReliefF, yellow: SVMAttributeEval, blue: wrapper (naive Bayes)), respectively. The classification quality achieved for classification based on all (290) pathways is marked by a horizontal line (black). Red boxes depict the quality of classification based on randomly chosen 1 to 20 pathways (average quality for 25 times). For classification, we applied the nearest neighbor classifier IB1, the decision tree classifier J48, the naive Bayes classifier, and the linear support vector machine SMO. The diagrams demonstrate that the identified most relevant pathways are well suited to distinguish (obligate) anaerobe and (obligate) aerobe species (max(ROC AUC) = 0.81). The increase in classification quality for the rising number of randomly picked pathways indicates that the over-all metabolism is similar within the group of anaerobe species and distinctive when compared to the group of aerobe species.

Table S4: Relevant Pathways for Obligate Anaerobicity

method	relevant pathways
ReliefF	Biosynthesis of coproporphyrin I (pyrrole6)<br>Biosynthesis of coproporphyrin III (pyrrole8)<br>Heme biosynthesis (pyrrole3)<br>beta-Oxidation of fatty acids (fa2)<br>Biosynthesis of 5-aminolevulinate from L-glutamate (pyrrole1)
SVMAttributeEval	Degradation of L-isoleucin (vas5)<br>Degradation of mannose (gg8)<br>beta-Oxidation of fatty acids (fa2)<br>Glyoxylate cycle (part) (gc1)<br>Alcoholic fermentation of pyruvate (aar4)
wrapper (naive Bayes)	Degradation of L-lysine to crotonyl-CoA (lysine3)<br>Glyoxylate cycle (part) (gc1)<br>Degradation of histidine to imidazoleacetate (histidine5)<br>Biosynthesis of L-aspartate (aspartate1)<br>Degradation of urea (urea6)

The relevant pathways for (obligate) anaerobicity were determined by applying three different attribute selection methods (ReliefF, SVMAttributeEval, and a wrapper for the naive Bayes classifier) to the set of pathway profiles representing all (obligate) anaerobe and (obligate) aerobe genomes (113). Here, the five most relevant pathways identified by the different attributes selection methods are listed, respectively.

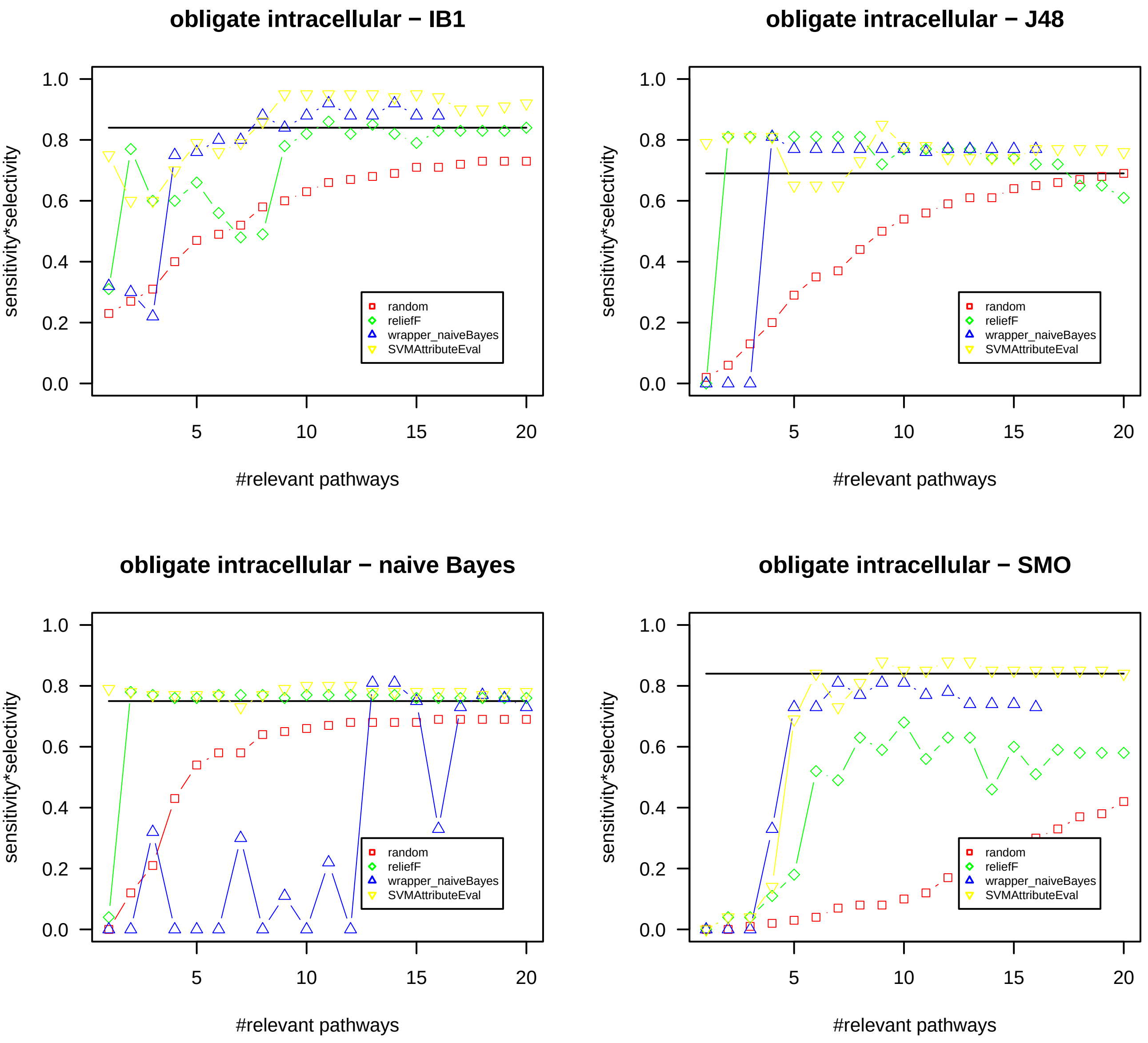

Figure S21: Cross-checking the Most Relevant Pathways for Obligate Intracellularity by Classification. The diagrams show the classification quality (assessed by the product of sensitivity and selectivity) for the classification of the completely sequenced genomes (266) into species that are obligate intracellular and those that are not. The classification is based on reduced pathway profiles containing only the 1 to 20 most relevant pathways (green: ReliefF, yellow: SVMAttributeEval, blue: wrapper (naive Bayes)), respectively. The classification quality achieved for classification based on all (290) pathways is marked by a horizontal line (black). Red boxes depict the quality of classification based on randomly chosen 1 to 20 pathways (average quality for 25 times). For classification, we applied the nearest neighbor classifier IB1, the decision tree classifier J48, the naive Bayes classifier, and the linear support vector machine SMO. According to the cross-check, the most relevant pathways identified by attribute selection are considered as significant (see Methods). The increase in classification quality for the rising number of randomly picked pathways indicates that the over-all metabolism is similar within the group of obligate intracellular species and distinctive when compared to other species.

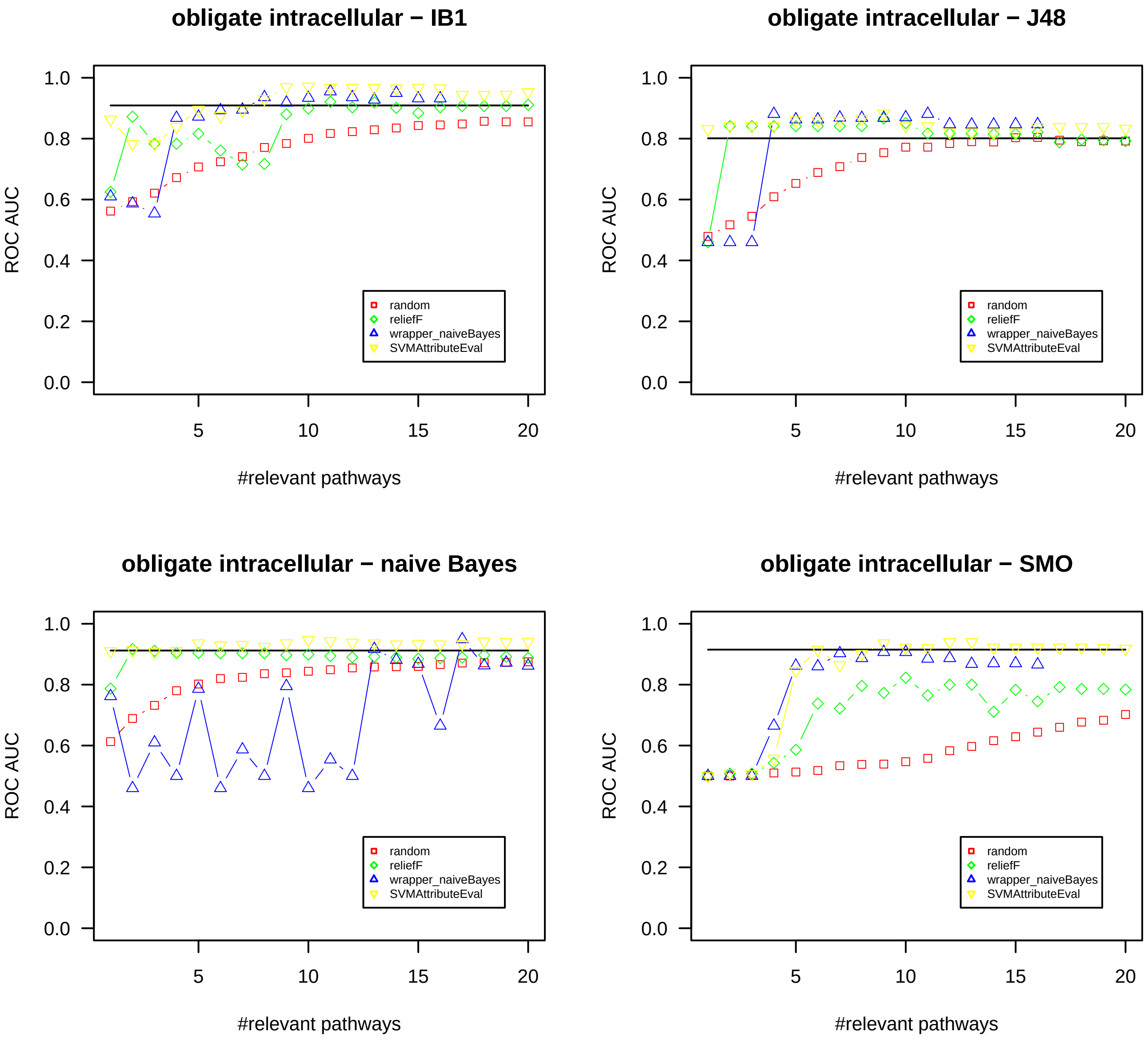

Figure S22: Cross-checking the Most Relevant Pathways for Obligate Intracellularity by Classification Using ROC AUC Values. The diagrams show the classification quality (assessed by the ROC AUC value) for the classification of the completely sequenced genomes (266) into species that are obligate intracellular and those that are not. The classification is based on reduced pathway profiles containing only the 1 to 20 most relevant pathways (green: ReliefF, yellow: SVMAttributeEval, blue: wrapper (naive Bayes)), respectively. The classification quality achieved for classification based on all (290) pathways is marked by a horizontal line (black). Red boxes depict the quality of classification based on randomly chosen 1 to 20 pathways (average quality for 25 times). For classification, we applied the nearest neighbor classifier IB1, the decision tree classifier J48, the naive Bayes classifier, and the linear support vector machine SMO. The diagrams demonstrate that the identified (up to) five most relevant pathways are already well suited to distinguish obligate intracellular species from other species (max(ROC AUC) = 0.92) . The increase in classification quality for the rising number of randomly picked pathways indicates that the over-all metabolism is similar within the group of obligate intracellular species and distinctive when compared to other species.

Table S5: Relevant Pathways for Obligate Intracellularity

method	relevant pathways
ReliefF	Conversion of L-glutamate to L-ornithine (glutamate2)<br>Oxidative ethanol degradation (glf4)<br>Conversion of L-glutamate to L-proline (glutamate3)<br>Biosynthesis of L-proline (proline1)<br>Degradation of L-aspartate (aspartate2)
SVMAttributeEval	Oxidative ethanol degradation (glf4)<br>Conversion of L-glutamate to L-proline (glutamate3)<br>Biosynthesis of L-proline (proline1)<br>Conversion of estrone into estradiol (steroidhormones13)<br>Biosynthesis of protoporphyrin IX (pyrrole9)
wrapper (naive Bayes)	Degradation of L-lysine to crotonyl-CoA (lysine3)<br>Phosphorylation of arginine (arginine3)<br>Biosynthesis of protoporphyrin IX (pyrrole9)<br>omega-Oxidation of alkanes (fa3)<br>Oxidative ethanol degradation (glf4)

The relevant pathways for obligate intracellularity were determined by applying three different attribute selection methods (ReliefF, SVMAttributeEval, and a wrapper for the naive Bayes classifier) to the complete set of pathway profiles (266 genomes). Here, the five most relevant pathways identified by the different attributes selection methods are listed, respectively.

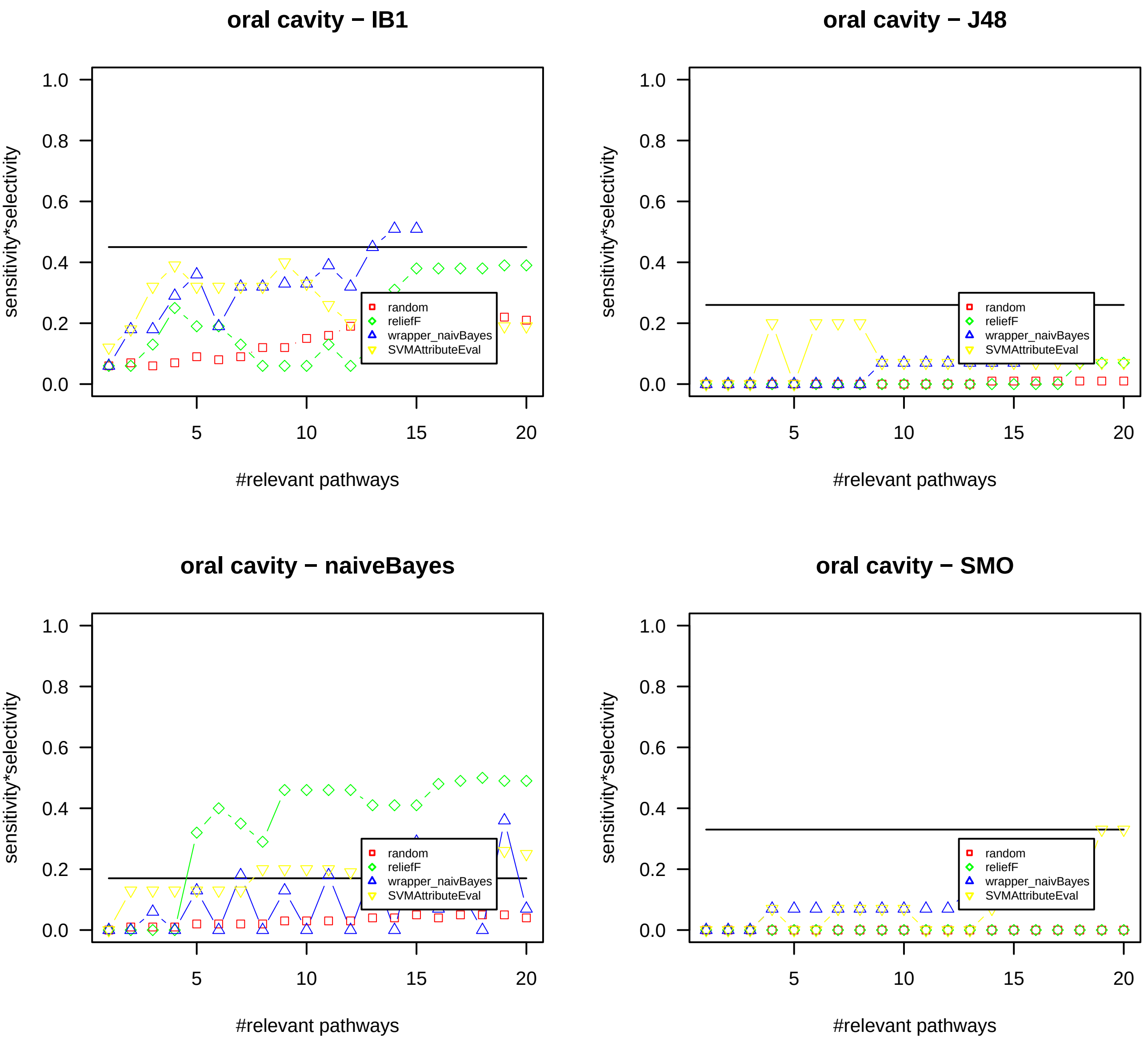

Figure S23: Cross-checking the Most Relevant Pathways for Oral Cavity Topism by Classification. The diagrams show the classification quality (assessed by the product of sensitivity and selectivity) for the classification of the completely sequenced genomes (266) into species living in the oral cavity and species not living in this habitat. The classification is based on reduced pathway profiles containing only the 1 to 20 most relevant pathways (green: ReliefF, yellow: SVMAttributeEval, blue: wrapper (naive Bayes)), respectively. The classification quality achieved for classification based on all (290) pathways is marked by a horizontal line (black). Red boxes depict the quality of classification based on randomly chosen 1 to 20 pathways (average quality for 25 times). For classification, we applied the nearest neighbor classifier (IB1), the decision tree classifier J48, the naive Bayes classifier, and the linear support vector machine SMO. The product of sensitivity and selectivity did not reach 0.6 for any of the four classifiers. Thus, according to the cross-check, the most relevant pathways identified by attribute selection are not considered as significant (see Methods).

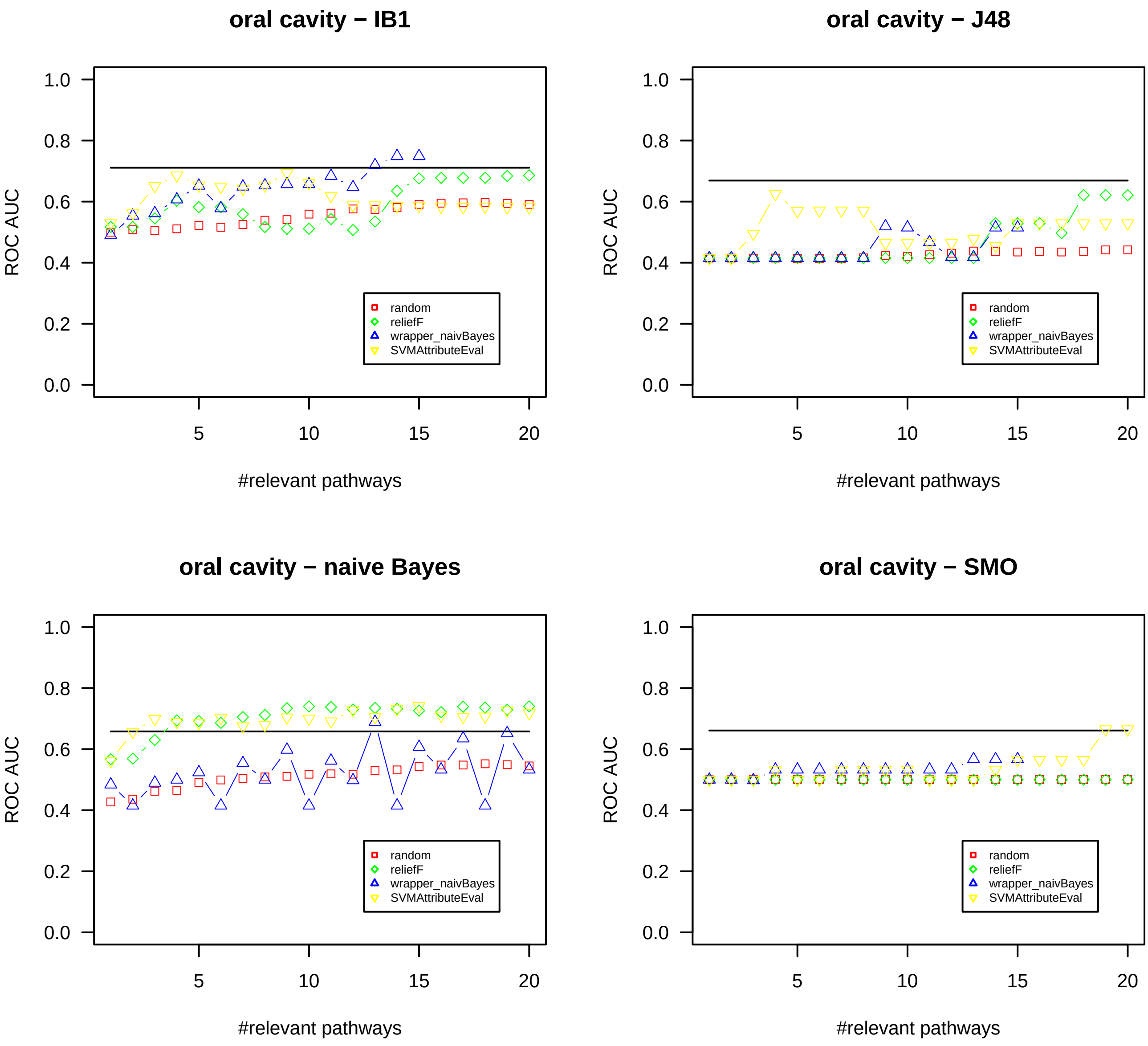

Figure S24: Cross-checking the Most Relevant Pathways for Oral Cavity Topism by Classification Using ROC AUC Values. The diagrams (266) show the classification quality (assessed by the ROC AUC value) for the classification of the completely sequenced genomes into species living in the oral cavity and species not living in this habitat. The classification is based on reduced pathway profiles containing only the 1 to 20 most relevant pathways (green: ReliefF, yellow: SVMAttributeEval, blue: wrapper (naive Bayes)), respectively. The classification quality achieved for classification based on all (290) pathways is marked by a horizontal line (black). Red boxes depict the quality of classification based on randomly chosen 1 to 20 pathways. For classification, we used the nearest neighbor classifier IB1, the decision tree classifier J48, the naive Bayes classifier, and the linear support vector machine SMO.

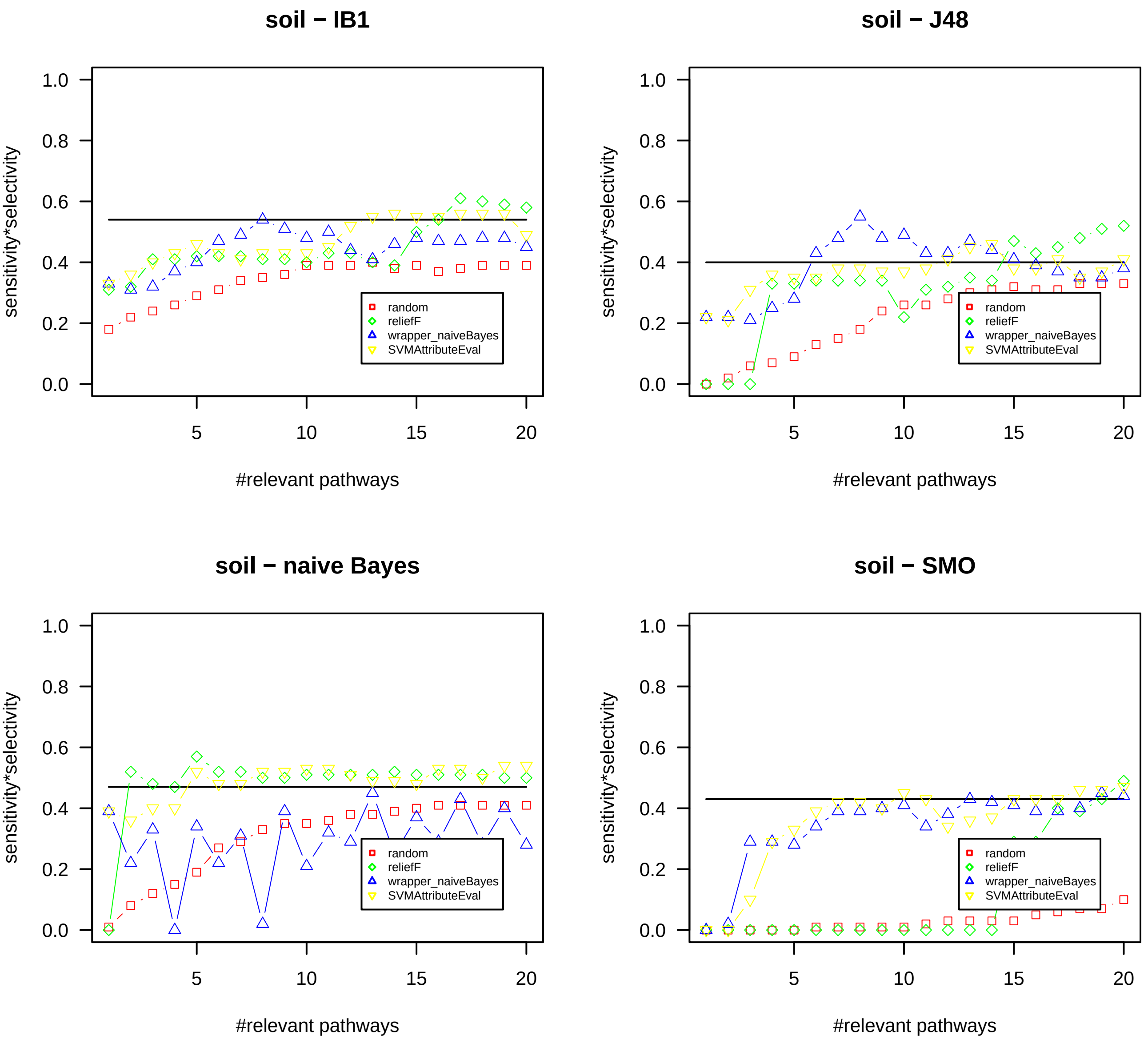

Figure S25: Cross-checking the Most Relevant Pathways for the Habitat Soil by Classification. The diagrams show the classification quality (assessed by the product of sensitivity and selectivity) for the classification of the completely sequenced genomes (266) into species living in the soil and species not living in this habitat. The classification is based on reduced pathway profiles containing only the 1 to 20 most relevant pathways (green: ReliefF, yellow: SVMAttributeEval, blue: wrapper (naive Bayes)), respectively. The classification quality achieved for classification based on all (290) pathways is marked by a horizontal line (black). Red boxes depict the quality of classification based on randomly chosen 1 to 20 pathways (average quality for 25 times). For classification, we applied the nearest neighbor classifier IB1, the decision tree classifier J48, the naive Bayes classifier, and the linear support vector machine SMO. The product of sensitivity and selectivity did not reach 0.6 for any of the four classifiers. Thus, according to the cross-check, the most relevant pathways identified by attribute selection are not considered as significant (see Methods).

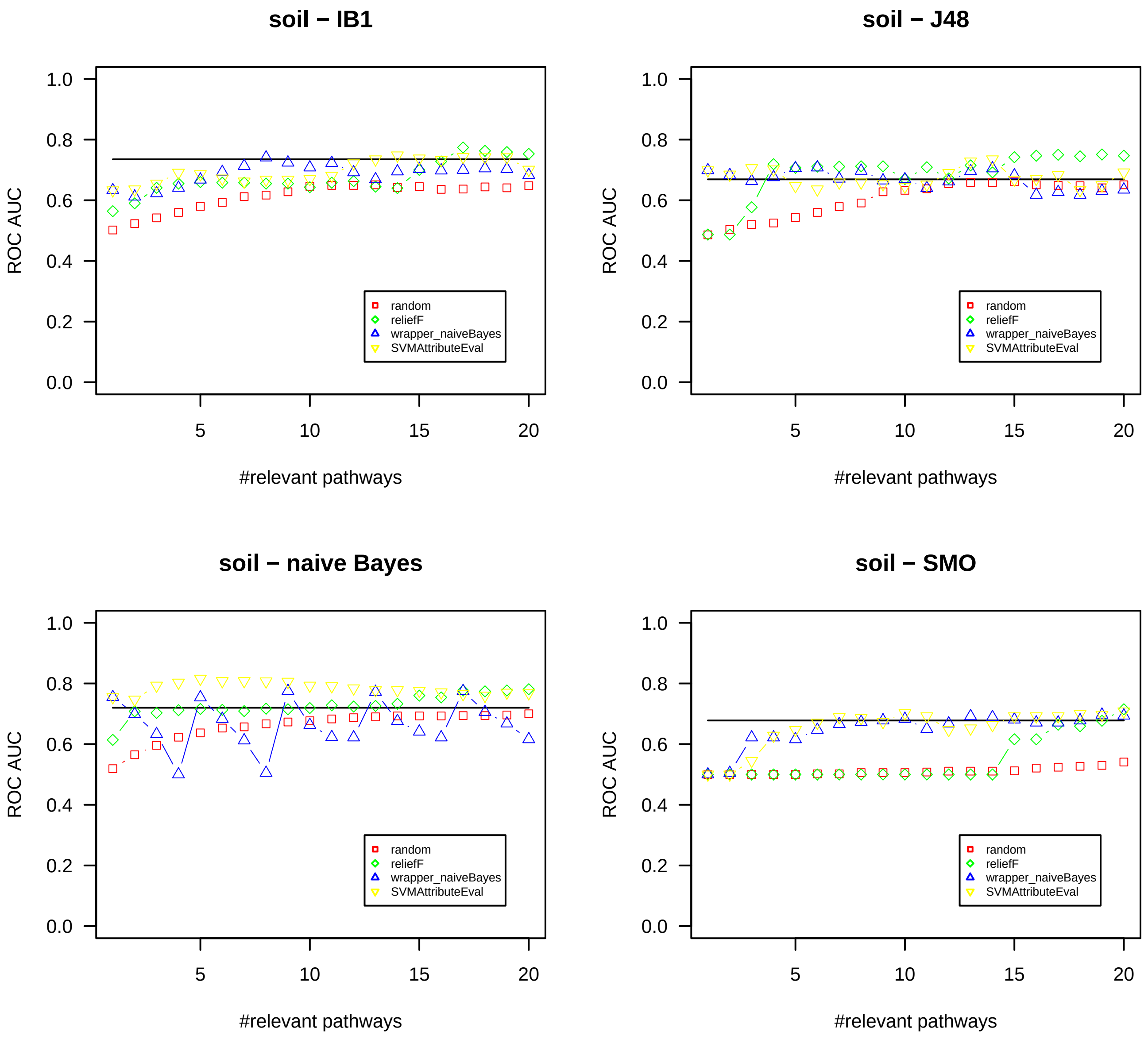

Figure S26: Cross-checking the Most Relevant Pathways for the Habitat Soil by Classification. The diagrams show the classification quality (assessed by the ROC AUC value) for the classification of the completely sequenced genomes (266) into species living in the soil and species not living in this habitat. The classification is based on reduced pathway profiles containing only the 1 to 20 most relevant pathways (green: ReliefF, yellow: SVMAttributeEval, blue: wrapper (naive Bayes)), respectively. The classification quality achieved for classification based on all (290) pathways is marked by a horizontal line (black). Red boxes depict the quality of classification based on randomly chosen 1 to 20 pathways (average quality for 25 times). For classification, we applied the nearest neighbor classifier IB1, the decision tree classifier J48, the naive Bayes classifier, and the linear support vector machine SMO.
