# Supplementary material for: Uncovering metabolic pathways relevant to phenotypic traits of microbial genomes
Source: Genome Biol. 2009 Mar 10;10(3):R28. doi: 10.1186/gb-2009-10-3-r28 (PMC2690999; doi:10.1186/gb-2009-10-3-r28)
Supplement: Additional data file 3 — The procedure used for evaluating the automatic metabolic reconstruction part of our method and the evaluation results obtained. [file gb-2009-10-3-r28-S3.pdf]

Evaluation of the score-based metabolic reconstruction method

For judging the quality of automatic metabolic reconstructions, one has to compare the pathway predictions to the knowledge on experimentally verified pathways for the respective genomes. However, such knowledge is limited to several well studied pathways in only a few model organisms. (Among them, *Escherichia coli K12* is presumably the best investigated species.) Most of this pathway information is distributed over many scientific publications. As a consequence, a systematic evaluation of metabolic reconstructions by an automated comparison to reliable experimental evidences is currently infeasible. Thus, several strategies have been followed for evaluation so far: (i) For the evaluation of PathoLogic pathway predictions for the genomes of *Helicobacter pylori* and *Methanococcus jannaschii*, the predictions have been compared to previous manual or automatic metabolic reconstructions, respectively [24,95]. (ii) The reliability of pathway predictions provided by metaSHARK [96] were demonstrated by the comparison of the predictions for the biosynthesis of coenzyme A in *Plasmodium falciparum* and *Eimeria tenella* to experimental evidences [96]. (iii) PathoLogic pathway predictions for the pathogen *Vibrio cholerae* were compared to experimental evidences from gene expression data and clinical tests [97]. (Thereby, experimental evidence supporting more than half of the predicted pathways was reported).

For the evaluation of our score-based metabolic reconstruction procedure, we chose the following strategy:

1. We performed a literature search to find experimental information on the presence or absence of the well studied pantothenate pathway in the completely sequenced genomes provided by PEDANT [23]. These evidences have then be compared to our score-based predictions for this pathway.

2. We performed a literature search to find experimental or reliable homology evidences for all enzymes involved in two well studied pathways (mevalonate pathway, Embden-Meyerhof pathway) for nine selected model organisms. These evidences have then be compared to the EC number assignments by PEDANT. (Moreover, we compared the automatic EC number assignments for four genomes in PEDANT to the manual annotations which are also provided in PEDANT for these genomes. The results of this comparison are shown in the Methods section of the main manuscript.)

3. We compared the pathway predictions for all pathways in BioPath [30] (i.e. the complete metabolic reconstruction) based on automatic EC numbers to the predictions based on manually assigned EC numbers for two species, for which manual EC number assignments have been available in PEDANT.

4. We manually compared our metabolic reconstruction for *M.jannaschii* to the reconstruction by PathoLogic provided within the BioCyc database [25]. Additionally, we compared our predictions for *Escherichia coli K12* to the data in the manually curated EcoCyc [77] database. Thereby, we compared only those BioPath pathways that we could map onto an EcoCyc pathway by an automated procedure.

For evaluating the predictive power of our score-based metabolic reconstruction procedure, we predicted the presence of a pathway if the pathway received a score of at least 0.5 (in analogy to the PathoLogic algorithm). (Please note that we did not use predictions on the presence or absence of pathways (and thus did not apply any score threshold) in our approach for the detection of relevant pathways. In contrast, we therein directly compared the predicted completeness of the pathways (represented by the respective pathway score).)

Prediction of the pantothenate pathway for PEDANT genomes

In order to evaluate the score-based pathway prediction across the completely sequenced genomes available in PEDANT, we compared our predictions for the *de novo* biosynthesis of pantothenate to evidences from literature. Pantothenate is the most common precursor of coenzyme A, which is an important cofactor in numerous biochemical reactions for nearly all organisms. The pantothenate pathway is well suited for this evaluation as it has been intensively studied for many sequenced genomes [44, 98]. Figure S26 (A) shows the four reaction steps from aspartate and ketovalerate to pantothenate. Plants, fungi, and many bacteria are able to produce pantothenate from aspartate and ketovalerate. In contrast, animals depend on pantothenate (= vitamin B5) in their diet. Many microbes that show a parasitic lifestyle also lack the biosynthesis of pantothenate.

We calculated the pathway reconstruction scores for the 242 completely sequenced genomes (25 eukaryotes (including 1 plant and 2 animals), 18 archaea, 199 bacteria) that have been automatically annotated by PEDANT at the time of the evaluation. We predicted the presence of pantothenate biosynthesis for 156 genomes and its absence for 86 genomes. The 156 positive predictions included 20 of the 22 fungi and the only plant in the data set. For the fungus *Saccharomyces kudriavzevii IFO1802*, we received a pathway score of 0.4 and thus a false negative pathway prediction. For the intracellular, parasitic fungus *Encephalitozoon cuniculi*, we calculated a score of 0.0. However, in this case our prediction agreed with evidence from literature [44]. In agreement with the literature, we also predicted the absence of the pantothenate biosynthesis for the two animal genomes in the data set. By a literature survey, we found reliable information on the presence or absence of pantothenate biosynthesis for another 53 genomes. According to our predictions, 29 of these genomes (see Table S6) are able to produce pantothenate *de novo*, whereas 24 lack the pantothenate biosynthesis (see Table 7). All 53 predictions were confirmed by literature evidences. Thus, for 77 out of the 78 genomes listed in Table S6 and Table S7, our predictions were correct. The pathway reconstruction scores determined for the remaining genomes are shown in Table S8 and Table S9. Only for 10 (19) out of all 242 genomes, the pathway score was 0.4 (0.6) and thus in the range of our prediction threshold. Thereby, 7 of the 10 genomes with the score 0.4 are non-methanogenic archaea, which are known to code for at least part of the enzymes in the pantothenate pathway. Neither homology based nor non-homology based methods could identify the remaining enzymes of this pathway in archaea [44]. Using a score instead of presence/absence values, this type of "partial" information can be preserved for the comparison of metabolic reconstructions.

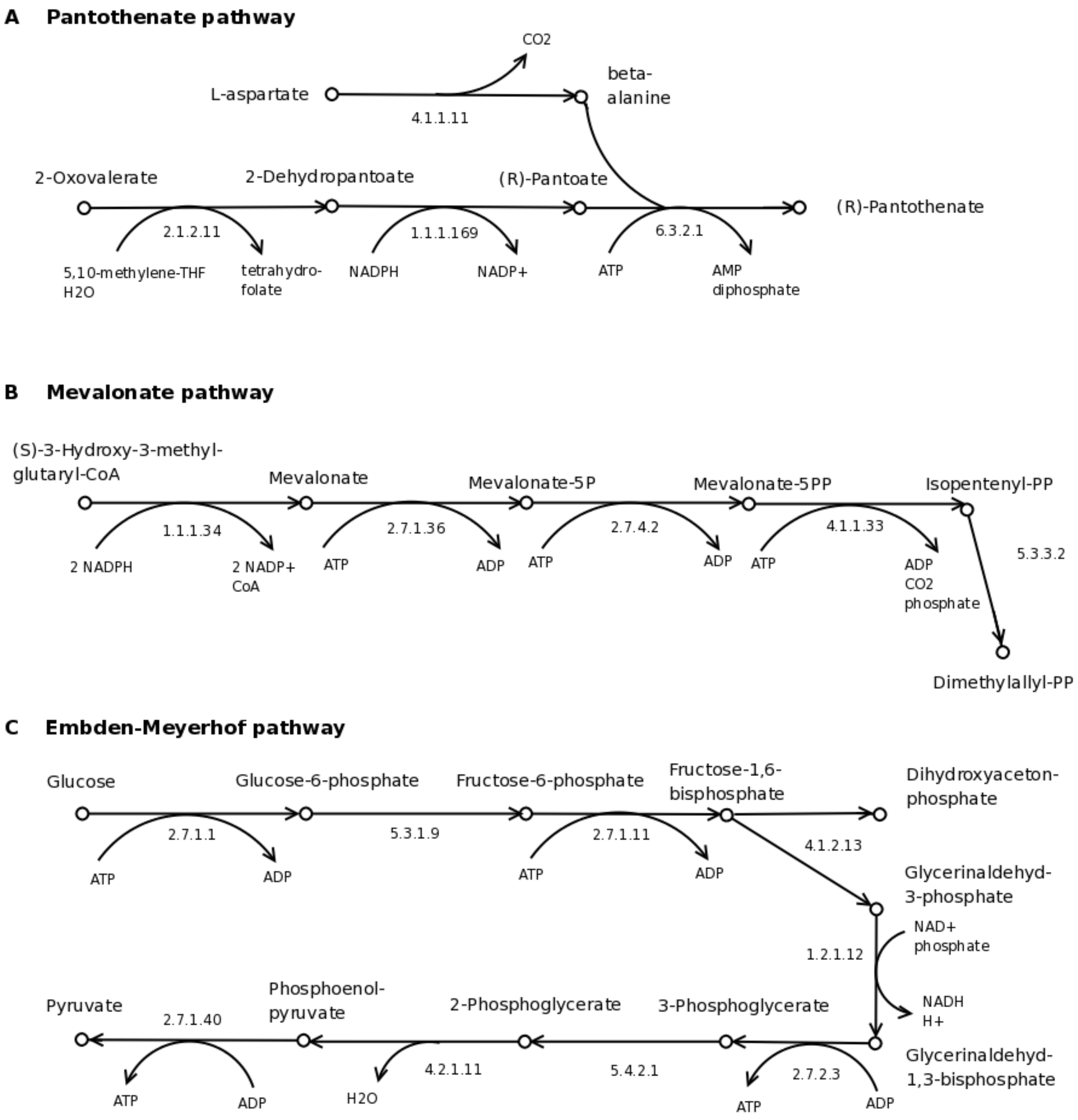

Figure S26: Evaluated pathways. (A) pantothenate pathway (B) mevalonate pathway (C) Embden-Meyerhof pathway

organism	path score	pre-diction	literature evidence
Arabidopsis thaliana	0.6	+ (+)	plant
Candida albicans SC 5314	1.0	+ (+)	fungus
Ashbya gossypii	0.8	+ (+)	fungus
Candida dubliniensis	0.8	+ (+)	fungus
Candida glabrata CBS 138	0.8	+ (+)	fungus
Coccidioides immitis	0.8	+ (+)	fungus
Coprinus cinereus	0.8	+ (+)	fungus
Debaryomyces hansenii CBS 767	0.8	+ (+)	fungus
Fusarium graminearum	0.8	+ (+)	fungus
Kluyveromyces lactis	0.8	+ (+)	fungus
Magnaporthe grisea BI	0.8	+ (+)	fungus
Phanerochaete chrysosporium	0.8	+ (+)	fungus
Saccharomyces bayanus MCYC 623	0.8	+ (+)	fungus
Saccharomyces castellii NRRL Y12630	0.8	+ (+)	fungus
Saccharomyces cerevisiae	0.8	+ (+)	fungus
Saccharomyces paradoxus NRRL Y17217	0.8	+ (+)	fungus
Aspergillus nidulans	0.6	+ (+)	fungus
Neurospora crassa	0.6	+ (+)	fungus
Saccharomyces kluyveri NRRL Y12651	0.6	+ (+)	fungus
Saccharomyces mikatae IFO1815	0.6	+ (+)	fungus
Ustilago maydis	0.6	+ (+)	fungus
Bacillus anthracis Ames	1.0	+ (+)	[99]
Bacillus anthracis str Sterne	1.0	+ (+)	[99]
Bacillus anthris Ames 0581	1.0	+ (+)	[99]
Bacillus subtilis 168	1.0	+ (+)	[100]
Escherichia coli CFT073	1.0	+ (+)	[101]
Escherichia coli K12	1.0	+ (+)	[101]
Escherichia coli O157 H7	1.0	+ (+)	[101]
Escherichia coli O157 H7 EDL933	1.0	+ (+)	[101]
Listeria monocytogenes 4b F2365	1.0	+ (+)	[102]
Listeria monocytogenes EGD-e	1.0	+ (+)	[102]
Mycobacterium avium paratuberculosis	1.0	+ (+)	[103]
Salmonella enterica ent. Typhi CT18	1.0	+ (+)	[101]
Salmonella enterica ent. Typhi Ty2	1.0	+ (+)	[101]
Salmonella typhimurium LT2	1.0	+ (+)	[101]
Salmonella typhi Ty2	1.0	+ (+)	[101]
Staphylococcus aureus N315	1.0	+ (+)	[99]
Staphylococcus aureus aureus MRSA252	1.0	+ (+)	[99]
Staphylococcus aureus aureus MSSA476	1.0	+ (+)	[99]
Staphylococcus aureus aureus MW2	1.0	+ (+)	[99]
Staphylococcus aureus Mu50	1.0	+ (+)	[99]
Pseudomonas aeruginosa PA01	1.0	+ (+)	[99]
Yersinia pestis CO92	1.0	+ (+)	[98]
Yersinia pestis KIM	1.0	+ (+)	[98]
Yersinia pseudotuberculosis IP32953	1.0	+ (+)	[98]
Agrobacterium tumefaciens C58 UWash	0.8	+ (+)	[104]
Corynebacterium glutamicum ATCC1303	0.8	+ (+)	[105]
Helicobacter pylori 26695	0.8	+ (+)	[99]
Wigglesworthia brevipalpis	0.8	+ (+)	[106]
Helicobacter pylori J99	0.6	+ (+)	[99]

Table S6: Positive Predictions. 50 organisms for which we found information about the *de novo* pantothenate pathway in the literature and for which the biosynthesis of pantothenate was predicted to be present.

organism	path score	pre-diction	literature evidence
Caenorhabditis elegans WS75	0.0	- (-)	animal
Drosophila melanogaster	0.0	- (-)	animal
Encephalitozoon cuniculi	0.0	- (-)	[44]
Saccharomyces kudriavzevii IFO1802	0.4	- (+)	fungus
Bifidobacterium longum NCC2705	0.0	- (-)	[107]
Borrelia burgdorferi B31	0.0	- (-)	[44]
Buchnera aphidicola Bp	0.0	- (-)	[106]
Candidatus Blochmannia floridanus	0.0	- (-)	[106]
Chlamydia trachomatis A HAR 13	0.0	- (-)	[99]
Chlamydia trachomatis	0.0	- (-)	[99]
Haemophilus influenzae 86 028NP	0.0	- (-)	[99]
Lactobacillus johnsonii NCC 533	0.0	- (-)	[107]
Mannheimia succiniciproducens	0.0	- (-)	[108]
Methanopyrus kandleri AV19	0.0	- (-)	[44]
Methanosarcina acetivorans C2A	0.0	- (-)	[44]
Methanosarcina mazei Goe1	0.0	- (-)	[44]
Methanothermobacter thermautotrophicus	0.0	- (-)	[44]
Mycoplasma genitalium G37	0.0	- (-)	[99]
Rickettsia conorii Malish 7	0.0	- (-)	[99]
Rickettsia prowazekii Madrid E	0.0	- (-)	[99]
Rickettsia typhi Wilmington	0.0	- (-)	[99]
Streptococcus pneumoniae R6	0.0	- (-)	[99]
Streptococcus pneumoniae TIGR4	0.0	- (-)	[99]
Thermoplasma acidophilum	0.0	- (-)	[44]
Methanococcus maripaludis	0.2	- (-)	[44]
Propionibacterium acnes	0.2	- (-)	[109]
Streptococcus thermophilus CNRZ1066	0.2	- (-)	[110]
Streptococcus thermophilus LMG 18311	0.2	- (-)	[110]

Table S7: Negative predictions. 28 organisms for which we found information about the *de novo* pantothenate pathway in the literature and for which the biosynthesis of pantothenate was predicted to be absent.

organism	pathway score
Anabaena variabilis	1.0
Aquifex aeolicus VF5	1.0
Bacillus cereus ATCC 10987	1.0
Bacillus cereus ATCC14579	1.0
Bacillus cereus ZK	1.0
Bacillus clausii KSM K16	1.0
Bacillus licheniformis ATCC 14580	1.0
Bacillus licheniformis DSM 13	1.0
Bacillus thuringiensis konkukian	1.0
Bacillus halodurans	1.0
Bordetella bronchiseptica RB50	1.0
Bordetella parapertussis 12822	1.0
Bordetella pertussis Tohama I	1.0
Burkholderia pseudomallei K96243	1.0
Clostridium acetobutylicum ATCC824	1.0
Corynebacterium efficiens YS314T	1.0
Corynebacterium glutamicum	1.0
Desulfotalea psychrophila	1.0
Enterococcus faecalis V583	1.0
Erwinia carotovora atroseptica SCRI1043	1.0
Geobacillus kaustophilus HTA426	1.0
Leptospira interrogans 56601	1.0
Leptospira interrogans serovar Copenhageni	1.0
Listeria innocua Clip11262	1.0
Mesorhizobium loti MAFF303099	1.0
Methylococcus capsulatus Bath	1.0
Mycobacterium bovis AF2122	1.0
Nocardia farcinica IFM10152	1.0
Oceanobacillus iheyensis	1.0
Photorhabdus luminescens laumondii TTO1	1.0
Pirellula sp	1.0
Porphyromonas gingivalis W83	1.0
Pseudomonas fluorescens Pf5	1.0
Shigella flexneri 2a 2457T	1.0
Staphylococcus epidermidis ATCC12228	1.0
Staphylococcus epidermidis RP62A	1.0
Staphylococcus haemolyticus	1.0
Streptomyces avermitilis MA4680	1.0
Acinetobacter ADP1	0.8
Anabaena sp PCC7120	0.8
Azoarcus sp EbN1	0.8
Bacteroides fragilis NCTC 9434	0.8
Bacteroides fragilis YCH46	0.8
Bacteroides thetaiotaomicron VPI5482	0.8
Bdellovibrio bacteriovorus	0.8
Campylobacter jejuni NCTC 11168	0.8

Campylobacter jejuni RM1221	0.8
Caulobacter crescentus	0.8
Chlorobium tepidum TLS	0.8
Chromobacterium violaceum ATCC12472	0.8
Colwellia psychrerythraea 34H	0.8
Corynebacterium jeikeium K411	0.8
Deinococcus radiodurans R1	0.8
Francisella tularensis tularensis	0.8
Geobacter sulfurreducens	0.8
Gloeobacter violaceus PCC7421	0.8
Helicobacter hepaticus ATCC51449	0.8
Idiomarina loihiensis L2TR	0.8
Legionella pneumophila Lens	0.8
Legionella pneumophila Paris	0.8
Legionella pneumophila Philadelphia1	0.8
Leifsonia xyli xyli CTCB0	0.8
Mycobacterium leprae TN	0.8
Neisseria gonorrhoeae FA 1090	0.8
Neisseria meningitidis Z2491	0.8
Photobacterium profundum SS9	0.8
Pseudomonas putida KT2440	0.8
Pseudomonas syringae pv B728a	0.8
Pseudomonas syringae tomato DC3000	0.8
Rhodopseudomonas palustris CGA009	0.8
Shewanella oneidensis MR1	0.8
Silicibacter pomeroyi DSS 3	0.8
Synechocystis PCC6803	0.8
Thermus thermophilus HB27	0.8
Thermus thermophilus HB8	0.8
Vibrio fischeri ES114	0.8
Vibrio parahaemolyticus O3K6	0.8
Vibrio vulnificus CMCP6	0.8
Vibrio vulnificus YJ016	0.8
Wolinella succinogenes DSMZ1740	0.8
Xanthomonas axonopodis citri 306	0.8
Xanthomonas campestris 8004	0.8
Xanthomonas campestris campestris ATCC33913	0.8
Xanthomonas oryzae KACC10331	0.8
Xylella fastidiosa 9a5c	0.8
Xylella fastidiosa Temecula1	0.8
Anaplasma marginale St Maries	0.6
Bartonella henselae Houston 1	0.6
Bartonella quintana	0.6
Brucella abortus 9 941	0.6
Brucella melitensis 16M	0.6
Buchnera sp APS	0.6
Candidatus Pelagibacter ubique HTCC1062	0.6
Desulfovibrio vulgaris Hildenborough	0.6

Nitrobacter winogradskyi Nb 255	0.6
Nitrosomonas europaea ATCC19718	0.6
Prochlorococcus marinus marinus CCMP1375	0.6
Prochlorococcus marinus MED4	0.6
Prochlorococcus marinus MIT9313	0.6
Sinorhizobium meliloti 1021	0.6
Synechococcus elongatus PCC 6301	0.6
Synechococcus sp WH8102	0.6
Thermosynechococcus elongatus BP1	0.6
Tropheryma whipplei TW08 27	0.6
Tropheryma whipplei Twist	0.6
Zymomonas mobilis ZM4	0.6

Table S8: Positive predictions without evidences. 106 organisms for which we did not find any information about the *de novo* pantothenate pathway in the literature and for which the biosynthesis of pantothenate was predicted to be present.

organism	pathway score
Actinobacillus actinomycetemcomitans HK1651	0.0
Borrelia garinii PBi	0.0
Chlamydia muridarum	0.0
Chlamydophila caviae GPIC	0.0
Chlamydophila pneumoniae CWL029	0.0
Chlamydophila pneumoniae J138	0.0
Chlamydophila pneumoniae TW183	0.0
Clostridium tetani E88	0.0
Ehrlichia ruminantium Gardel	0.0
Ehrlichia ruminantium str Welgevonden	0.0
Ehrlichia ruminantium Welgevonden	0.0
Fusobacterium nucleatum ATCC25586	0.0
Haemophilus ducreyi 35000HP	0.0
Lactobacillus acidophilus NCFM	0.0
Mesoplasma florum	0.0
Mycoplasma gallisepticum R	0.0
Mycoplasma hyopneumoniae 232	0.0
Mycoplasma hyopneumoniae J	0.0
Mycoplasma mobile 163K	0.0
Mycoplasma mycoides	0.0
Mycoplasma penetrans	0.0
Mycoplasma pneumoniae M129	0.0
Nanoarchaeum equitans	0.0
Onion yellows phytoplasma	0.0
Parachlamydia sp UWE25	0.0
Pasteurella multocida PM70	0.0
Picrophilus torridus DSM 9790	0.0
Streptococcus mutans UA159	0.0
Symbiobacterium thermophilum IAM14863	0.0

Thermoanaerobacter tengcongensis MB4T	0.0
Treponema pallidum Nichols	0.0
Ureaplasma urealyticum serovar3	0.0
Wolbachia endosymbiont of Brugia malayi TRS	0.0
Wolbachia endosymbiont of Drosophila melanogaster	0.0
Archaeoglobus fulgidus	0.2
Lactococcus lactis lactis IL1403	0.2
Mycoplasma pulmonis	0.2
Streptococcus agalactiae 2603	0.2
Streptococcus agalactiae A909	0.2
Streptococcus agalactiae NEM316	0.2
Streptococcus pyogenes MGAS10394	0.2
Streptococcus pyogenes MGAS315	0.2
Streptococcus pyogenes MGAS8232	0.2
Streptococcus pyogenes SF370	0.2
Streptococcus pyogenes SSI1	0.2
Sulfolobus acidocaldarius DSM 639	0.2
Sulfolobus solfataricus P2	0.2
Sulfolobus tokodaii	0.2
Treponema denticola ATCC 35405	0.2
Aeropyrum pernix K1	0.4
Gluconobacter oxydans 621H	0.4
Haloarcula marismortui ATCC 43049	0.4
Halobacterium NRC 1	0.4
Lactobacillus plantarum WCFS1	0.4
Natronomonas pharaonis DSM 2160	0.4
Pyrobaculum aerophilum IM2	0.4
Pyrococcus furiosus DSM3638	0.4
Thermococcus kodakaraensis KOD1	0.4

Table S9: Negative predictions without evidences. 58 organisms for which we did not find any information about the *de novo* pantothenate pathway in the literature and for which the biosynthesis of pantothenate was predicted to be absent.

PEDANT EC number assignments

For nine model organisms (listed in Table S10) and two well studied pathways (mevalonate and Embden-Meyerhof pathway), we inferred the presence or absence of the pathway enzymes according to the respective PEDANT EC number assignments. For each pathway and each enzyme involved, we searched for experimental or reliable homology evidences in the literature. (Thereby, we only searched for the evidence regarding presence or absence of any corresponding enzyme in the literature. We did not check the correctness of PEDANT predictions with respect to the genetic element, for which the EC number has been predicted.)

(Additionally, we compared automatic EC number assignments by PEDANT to manual EC number annotations (by the MIPS annotation group) for four organisms (see the main

manuscript).

organism	abbrev.	domain	short taxonomy
Saccharomyces cerevisiae	SC	Eukaryota	Fungi/Metazoa group/Fungi
Arabidopsis thaliana MAtDB	AT	Eukaryota	Viridiplantae/Streptophyta
Methanothermobacter thermo-autotrophicus	MT	Archaea	Euryarchaeota/Methanobacteria
Bacillus subtilis 168	BS	Bacteria	Firmicutes/Bacilli
Escherichia coli K12	EC	Bacteria	Proteobacteria/gamma
Helicobacter pylori 26695	HP	Bacteria	Proteobacteria/epsilon
Haemophilus influenzae 86 028NP	HI	Bacteria	Proteobacteria/gamma
Mycoplasma genitalium G37	MG	Bacteria	Firmicutes/Mollicutes
Chlamydia trachomatis A HAR 13	CT	Bacteria	Chlamydiae/Verrucomicrobia group/Chlamydiae

Table S10: Model organisms used for the evaluation of the PEDANT EC number assignments. In order to cover all domains of life, we chose a fungus, a plant, an archaeon, and several pathogenic and non-pathogenic bacteria from different taxonomic groups.

The evaluation results for the mevalonate pathway and for the Embden-Meyerhof pathway are shown in Table S11 and S12, respectively. In these tables all mismatches between prediction and literature evidence are indicated by blue (false positives) and red (false negatives) boxes. We also provide the pathway reconstruction scores, which have been determined for the pathway based on the PEDANT EC number assignments.

The mevalonate pathway, which is shown in Figure S26 (B), describes one of the two known biological processes (starting from different educts) for the biosynthesis of isopentenyl diphosphate. Isopentenyl diphosphate is a precursor of isoprenoids and thus plays an important role in the steroid biosynthesis. The methylerithol or glyceraldehyde 3-phosphate pathway is the alternative route to this precursor and shares only one reaction (EC 5.3.3.2) with the mevalonate pathway. Apart from plants, most organisms can only use either of these processes for the isopentenyl diphosphate biosynthesis. The mevalonate pathway is usually operative in archaea [123], fungi [124], animals [111], and plants [125] (which is reflected by a high pathway score) and absent in most eubacteria. Thus, *Escherichia coli*, *Chlamydia trachomatis*, *Bacillus subtilis*, and *Mycobacterium tuberculosis* lack this pathway [117, 126].

For the enzymes of the mevalonate pathway, we encountered two wrong predictions out of 45 EC number predictions in total. In both cases (EC 4.1.1.33 and EC 5.3.3.2), PEDANT missed the enzymes (false negatives).

The glycolysis is the general route for the degradation of glucose to pyruvate. It is the initial process of many other carbohydrate pathways. In several variants, glycolysis occurs in nearly all organisms. For our analysis, we used the standard glycolytic pathway as known in higher organisms (see Figure S26) [146]. This standard pathway is often referred to as Embden-Meyerhof pathway.

For the enzymes of the Embden-Meyerhof pathway, we encountered only 3 wrong predictions out of 81 EC number predictions in total. All three enzymes have been missed by the PEDANT prediction (false negatives).

organism	1.1.1.34	2.7.1.36	2.7.4.2	4.1.1.33	5.3.3.2	path score
AT	+ (+) [111]	+ (+) [112]	+ (+) [111]	+ (+) [111]	+ (+) [113]	1.00
BS	- (-) [111]	- (-) [114]	- (-) [114]	- (-) [114]	- (+) [115]	0.00
CT	- (-) [111]	- (-) [111]	- (-) [111]	- (-) [111]	- (-) [116]	0.00
EC	- (-) [111]	- (-) [111]	- (-) [111]	- (-) [111]	+ (+) [?]	0.07
HI	- (-) [117]	- (-) [117]	- (-) [117]	- (-) [117]	- (-) [116]	0.00
HP	- (-) [111]	- (-) [111]	- (-) [111]	- (-) [111]	- (-) [116]	0.00
MT	+ (+) [118]	+ (+) [118]	- (-) [118]	- (-) [118]	+ (+) [118]	0.87
MG	- (-) [111]	- (-) [111]	- (-) [111]	- (-) [111]	- (-) [111]	0.00
SC	+ (+) [111]	+ (+) [119]	+ (+) [120]	- (+) [121]	+ (+) [122]	0.93

Table S11: Mevalonate pathway: comparison of PEDANT enzyme predictions to evidences from literature. '+'/'-' indicates that the reaction (described by its EC number) can/cannot be catalyzed by the organism according to the PEDANT EC number predictions. '(+)'/'(-)' indicates that the reaction can/cannot be catalyzed by the organism according to the literature. The references are given in the line below. False negative and false positive predictions are marked red and blue, respectively. The corresponding pathway reconstruction scores are shown in the last column of the table. Scores equal to or greater than 0.5 are highlighted by yellow boxes.

Pathway predictions based on manual versus automatic EC number assignments

For several genomes, PEDANT provided both automatically predicted and manually annotated EC numbers [23]. The comparison of automatic and manual EC number assignments (which were assumed to be correct) resulted in the sensitivity values 86% and 75% for the genomes *Listeria monocytogenes EGD-e* and *Protochlamydia amoebophila UWE25*, respectively. The corresponding positive predictive values were 79% and 84% (see the main manuscript).

In order to estimate how the quality of protein annotation influences the results of pathway predictions, we compared the predictions based on manual EC number assignments to the predictions based on automatic EC number assignments for both genomes.

For *Listeria monocytogenes EGD-e*, 87 out of 290 pathways in BioPath have been predicted (i.e. $score(p) \geq 0.5$) for both, predictions based on manual and predictions based on automatic EC number assignments. In addition, 13 pathways have been predicted only for the automatic variant and 6 pathways only for the manual variant. These pathways are listed in Table S13. Assuming that the pathway predictions based on the manual EC number assignments are correct (which is not necessarily true), we can determine the sensitivity and the positive predictive value for the completely automated pathway pre-

org.	2.7.1.1	5.3.1.9	2.7.1.11	4.1.2.13	1.2.1.12	2.7.2.3	5.4.2.1	4.2.1.11	2.7.1.40	score
AT	+ (+) [127]	+ (+) [128]	+ (+) [129]	+ (+) [128]	+ (+) [127]	+ (+) [128]	+ (+) [127]	+ (+) [127]	+ (+) [127]	1.00
BS	- (-) [130]	+ (+) [131]	+ (+) [131]	+ (+) [131]	+ (+) [131]	+ (+) [131]	+ (+) [132]	+ (+) [131]	+ (+) [131]	0.89
CT	- (-) [133]	+ (+) [133]	- (+) [133]	- (+) [133]	+ (+) [134]	+ (+) [134]	+ (+) [133]	+ (+) [133]	+ (+) [134]	0.67
EC	- (-) [133]	+ (+) [135]	+ (+) [136]	+ (+) [137]	+ (+) [138]	+ (+) [139]	+ (+) [140]	+ (+) [141]	+ (+) [142]	0.89
HI	- (-) [143]	+ (+) [143]	+ (+) [143]	+ (+) [143]	+ (+) [143]	+ (+) [143]	+ (+) [143]	+ (+) [143]	+ (+) [143]	0.89
HP	- (-) [133]	+ (+) [133]	- (-) [133]	+ (+) [133]	+ (+) [133]	+ (+) [133]	+ (+) [133]	+ (+) [133]	- (-) [133]	0.67
MT	- (-) [133]	- (-) [133]	- (-) [133]	+ (+) [133]	- (+) [133]	+ (+) [133]	+ (+) [133]	+ (+) [133]	- (-) [133]	0.44
MG	- (-) [144]	+ (+) [144]	+ (+) [144]	+ (+) [144]	+ (+) [144]	+ (+) [144]	+ (+) [144]	+ (+) [144]	+ (+) [144]	0.89
SC	+ (+) [145]	+ (+) [145]	+ (+) [145]	+ (+) [145]	+ (+) [145]	+ (+) [145]	+ (+) [145]	+ (+) [145]	+ (+) [145]	1.00

Table S12: Embden-Meyerhof pathway: comparison of PEDANT enzyme predictions to evidences from literature. '+'/'-' indicates that the reaction (described by its EC number) can/cannot be catalyzed by the organism according to the PEDANT EC number predictions. '(+)'/'(-)' indicates that the reaction can/cannot be catalyzed by the organism according to the literature. The references are given in the line below. False negative and false positive predictions are marked red and blue, respectively. The corresponding pathway reconstruction scores are shown in the last column of the table. Scores equal to or greater than 0.5 are highlighted by yellow boxes.

diction compared to the semi-automatic pathway prediction based on manually assigned EC numbers. Thereby, we achieved a sensitivity of 0.94 and a positive predictive value of 0.87. This compares to the sensitivity of 0.86 and the positive predictive value of 0.79 for the automatic prediction of EC numbers versus manual EC numbers. Thus, the score-based pathway prediction seems to compensate some of the gaps and the overpredictions made by automatic EC number predictions.

For *Protochlamydia amoebophila UWE25*, 54 out of 290 pathways in BioPath have been predicted for both, predictions based on manual and automatic EC number assignments. 4 pathways have been predicted only for the pathway prediction derived from automatic EC number assignments and 8 pathways only for the predictions derived from manual EC number assignments (see Table S14). This results in a sensitivity of 0.87 and a positive predictive value of 0.93 for the completely automated pathway prediction. In comparison, the automatic EC number prediction reached a sensitivity of 0.75 and a positive predictive value of 0.84.

Unique pathways for prediction based on manual EC	Unique pathways for prediction based on automatic EC
Biosynthesis of N-acetyl-D-glucosamine-6-phosphate (aminosugars1)	Fructose metabolism IX (fructose9)
Biosynthesis of UDP-N-acetyl-D-glucosamine (aminosugars3)	Glycolysis and gluconeogenesis XIII (gg13)
Degradation of cellulose (cellulose2)	Biosynthesis of colanic acid building blocks (uronicacids1)
Biosynthesis of ornithine (urea5)	Alcoholic fermentation of pyruvate (aar4)
Degradation of L-threonine to glycine and acetaldehyde (threonine3)	Degradation of L-threonine to L-2-aminoacetate (threonine2)
Biosynthesis of 2'-deoxyuridine-5'-phosphate from uridine-5'-diphosphate (dun1)	Biosynthesis of L-proline (proline1)
	Conversion of L-glutamate to L-proline (glutamate3)
	Biosynthesis of isoprenoids XI (isoprenoids11)
	Biosynthesis of 2'-deoxyuridine-5'-phosphate (dun2)
	Degradation of ATP to adenosine (an2)
	Degradation of GTP to guanosine (gn2)
	Degradation of xanthosine-5'-phosphate to xanthine (xn2)
	Degradation of IMP to xanthine (in2)

Table S13: Unique pathways predicted for *Listeria monocytogenes EGD-e* based on manual and automatic EC number assignments, respectively.

Comparison of score-based metabolic reconstructions to previous reconstructions

In order to evaluate the score-based pathway prediction relying on PEDANT and BioPath with respect to the complete metabolic reconstruction of a genome, we compared our reconstruction for the archaeon *Methanococcus jannaschii* to the corresponding reconstruction by PathoLogic. For this purpose, we manually mapped the predicted BioPath pathways onto the pathways that have been predicted by PathoLogic (and vice versa).

We also mapped BioPath pathways that we predicted for *Escherichia coli K12* onto the pathways of the manually curated EcoCyc database [77] by an automated procedure.

Metabolic reconstruction for *Methanococcus jannaschii*

Based on the PEDANT EC numbers for *M.jannaschii*, we determined the pathway reconstruction scores of the 290 BioPath reference pathways. All pathways for which the pathway score reached at least 0.5 have been predicted to be present in the genome. In total, we predicted the presence of 53 pathways. Table S15 lists the 24 BioPath pathways which are supposed to be complete in *M.jannaschii* according to our predictions.

Unique pathways for prediction based on manual EC	Unique pathways for prediction based on automatic EC
Biosynthesis of UDP-N-acetyl-D-glucosamine (aminosugars3)	Fructose metabolism IX (fructose9)
Biosynthesis of colanic acid building blocks (uronicacids1)	Glycolysis and gluconeogenesis X (gg10)
Arginine metabolism III (arginine3)	Biosynthesis of L-serine (sg1)
Biosynthesis of L-lysine from L-aspartate (lysine1)	Biosynthesis of phosphatidylserine (phospholipids3)
Biosynthesis of chorismate (aaa1)	
Biosynthesis of ubiquinone (aaa3)	
Biosynthesis of 2'-deoxyuridine-5'-phosphate from uridine-5'-phosphate (dun1)	
Degradation of ATP to 2'-deoxyadenosine (dan2)	

Table S14: Unique pathways predicted for *Protochlamydia amoebophila UWE25* based on manual and automatic EC number assignments, respectively.

Table S16 lists the remaining 29 pathways that have been predicted by applying the score threshold.

For the evaluation, we used the metabolic reconstruction for *M.jannaschii* by PathoLogic as provided within the BioCyc database. (The reconstruction reported by Tsoka et al. [95] was not accessible at the time of our analysis.) Reconstructions in BioCyc are based on MetaCyc pathways. The pathway definitions in MetaCyc differ from the definitions in BioPath (see the main manuscript) complicating the comparison of the predictions.

Given the 57 pathways predicted for *M.jannaschii* according to BioCyc, we manually mapped the 53 predicted BioPath pathways onto the pathways in BioCyc (if possible). BioPath pathways which correspond to a predicted BioCyc pathway (or to parts of it) are designated with '+' in Table S15 and Table S16. As can be seen from these tables, the predictions for 14 out of 21 complete BioPath pathways and 15 out of 29 incomplete BioPath pathways (in total 29 out of 53) are predicted in both reconstructions. Pathways which have been predicted by our reconstruction procedure but not by PathoLogic are designated with '-' in Table S15 and Table S16 (10 complete, 14 incomplete). Those pathways for which there is evidence according to the metabolic reconstruction of *M.jannaschii* by Selkov et al. [147] are additionally marked by '*'. Thus, 12 of the pathways predicted by our reconstruction procedure are presumably correct but missed by PathoLogic. As described in the main manuscript, the prediction for the biosynthesis of phosphatidylserine (phospholipids3) might also be correct. In contrast, the prediction for the biosynthesis of amylopectin (starch1) is a false positive. (Since there is evidence for glycogen storage in *M.jannaschii* [147], this prediction is presumably due to the prediction of a glycogen-branching enzyme.) The biosynthesis of ubiquinone is predicted in our reconstruction due to an error in the definition of the BioPath pathway: all reactions except a reaction catalyzed by the chorismate synthase are wrongly marked as spontaneous reactions. Thus, the prediction can be explained by the presence of the chorismate synthase in *M.jannaschii*.

BioPath pathway	description	score(p)	mapped
vas3	Biosynthesis of L-leucine	1.00	+
arginine4	Synthesis of L-arginyl-tRNA	1.00	+
aspartatecl1	Aspartate cycle (Biosynthesis of arginine)	1.00	+
urea1	Urea cycle I	1.00	+
urea2	Urea cycle II	1.00	+
glutamate4	Synthesis of L-glutamyl-tRNA	1.00	+
threonine1	Biosynthesis of L-threonine	1.00	+
methionine4	Synthesis of L-methionyl-tRNA	1.00	+
lysine1	Biosynthesis of L-lysine from L-aspartate	1.00	-*
histidine1	Biosynthesis of histidine	1.00	-*
aaa3	Biosynthesis of ubiquinone I	1.00	-
dra1	Biosynthesis of deoxyribonucleic acid from dTTP	1.00	-
dra2	Biosynthesis of deoxyribonucleic acid from dCTP	1.00	-
ra2	Biosynthesis of ribonucleic acid from CTP	1.00	-
agdra1	Biosynthesis of deoxyribonucleic acid from dGTP	1.00	-
agdra2	Biosynthesis of deoxyribonucleic acid from dATP	1.00	-
fructose4	Fructose metabolism IV (glyceraldehyde-3-P/glycerone-P)	1.00	+
cc4	Citrate cycle IV	1.00	+
ppc3	Pentose phosphate cycle (non-oxidative branch)	1.00	+
ppc4	Pentose phosphate cycle IV	1.00	+
ppc5	Pentose phosphate cycle (M.pneumoniae)	1.00	+
uronicacids1	Synthesis of UDP-D-glucuronate	1.00	-*
pyrrole1	Biosynthesis of 5-aminolevulinate from L-glutamate	1.00	+
isoprenoids11	Biosynthesis of isoprenoids XI	1.00	+

Table S15: BioPath pathways predicted to be complete in *M.jannaschii*. Pathways whose reconstruction score reached 1.0 are listed. +/- in the last column denote whether the pathway could/could not be (manually) mapped onto a corresponding pathway predicted in BioCyc. (For the mapping see Table S17 and Table S18.) * designates pathways that have not been mapped but for which there is evidence according to [147]. The colors group pathways related to the categories *Amino acids and derivatives* (red), *Nucleotides and Nucleosides* (grey) and *Carbohydrate metabolism and citric acid cycle* (yellow).

From the perspective of the predicted BioCyc pathways, 29 out of 57 pathways could be mapped onto one ore more BioPath pathways. These BioCyc pathways are listed in Table S17. Whereas the predictions agree for 25 of these pathways (designated with 'y' in Table S17), our reconstruction procedure did not predict 4 of the pathways (designated with 'n' in Table S17).

For 28 out of the 57 pathways in BioCyc, we could not find a corresponding BioPath pathway. These pathways are listed in Table S18. In some cases (11 pathways designated with '?' in Table S18), we could not find a corresponding BioPath pathway due to major differences in the definitions of the pathways. This problem mainly affects common pathways in the carbohydrate metabolism. BioPath completely lacks the remaining 17 pathways in BioCyc (designated with '-' in Table S18. This comparatively high number of pathways (or pathway variants) that are not included in BioPath in comparison to BioCyc/MetaCyc shows that MetaCyc is more comprehensive compared to BioPath.

In summary, this evaluation of the score-based pathway prediction has shown the applicability of score-based metabolic reconstructions for predicting the metabolic capabilities of species. Regarding the percentage of overlapping, unique, presumably correct and pre-

BioPath pathway	description	score(p)	mapped
glutamate7	Conversion of L-glutatmate to L-glutamine	0.70	+
vas1	Biosynthesis of L-valine	0.52	+
aaa6	Biosynthesis of L-tryptophane from chorismate	0.85	+
aaa5	Biosynthesis of L-tyrosine from chorismate	0.60	+
aaa1	Biosynthesis of chorismate	0.57	+
sg5	Serine and glycine V	0.50	+
polyamine4	Biosynthesis of spermine from L-arginine	0.53	+
urea4	Degradation of arginine	0.80	+
urea5	Biosynthesis of ornithine	0.60	+
vas2	Biosynthesis of L-isoleucine	0.67	-*
aaa4	Biosynthesis of L-phenylalanine from chorismate	0.60	-*
sg1	Biosynthesis of L-serine	0.75	-*
cn1	Biosynthesis of cytidine-5'-triphosphate	0.59	+
dtn1	Biosynthesis of 2'-deoxythymidine-5'-triphosphate	0.50	-*
un1	Biosynthesis of uridine-5'-phosphate	0.80	-*
dan2	Degradation of ATP to 2-deoxyadenosine	0.71	-*
an1	Biosynthesis of ATP	0.75	-*
in1	Biosynthesis of IMP	0.86	-*
ra1	Biosynthesis of ribonucleic acid from UTP	0.60	-
agra1	Biosynthesis of ribonucleic acid from ATP	0.50	-
agra2	Biosynthesis of ribonucleic acid from GTP	0.50	-
cc2	Citrate cycle II	0.53	+
gg1	Glycolysis and gluconeogenesis I	0.67	+
gg4	Glycolysis and gluconeogenesis IV	0.67	+
gg11	Glycolysis and gluconeogenesis XI	0.50	-*
starch1	Biosynthesis of amylopectin	0.50	-
methane1	Reduction of CO2 to CH4	0.67	+
pyrrole2	Biosynthesis of protoporphyrine IX	0.92	+
phospholipids3	Biosynthesis of phosphatidylserine	0.75	-

Table S16: BioPath pathways predicted for *M.jannaschii* by threshold. Pathways with $0.5 \leq score(p) < 1.0$ are listed. +/- in the last column denote whether the pathway could/could not be (manually) mapped onto a corresponding pathway predicted in BioCyc. (For the mapping see Table S17 and Table S18.) designates pathways that have not been mapped but for which there is evidence according to [147]. The colors group pathways related to the categories *Amino acids and derivatives* (red), *Nucleotides and Nucleosides* (grey) and *Carbohydrate metabolism and citric acid cycle* (yellow).

sumably false predictions, this comparison is within the range found in other comparisons of metabolic reconstructions [24, 95].

Metabolic reconstruction for *Escherichia coli*

In order to evaluate the score-based metabolic reconstruction of *Escherichia coli K12*, we used the manually curated EcoCyc database [77]. In contrast to the previous example, we mapped BioPath pathways onto EcoCyc pathways by an automatic procedure. For this analysis, we assigned an EcoCyc pathway to a BioPath pathway if the EcoCyc pathway at least covered half of the BioPath pathway (with respect to the reactions involved in the pathway). (Please note that this is not necessarily a biological reasonable mapping (in contrast to the manual mapping in the previous section), since the overlap of the pathways might only contain unspecific enzymes.) Provided that the mapping is reasonable, a

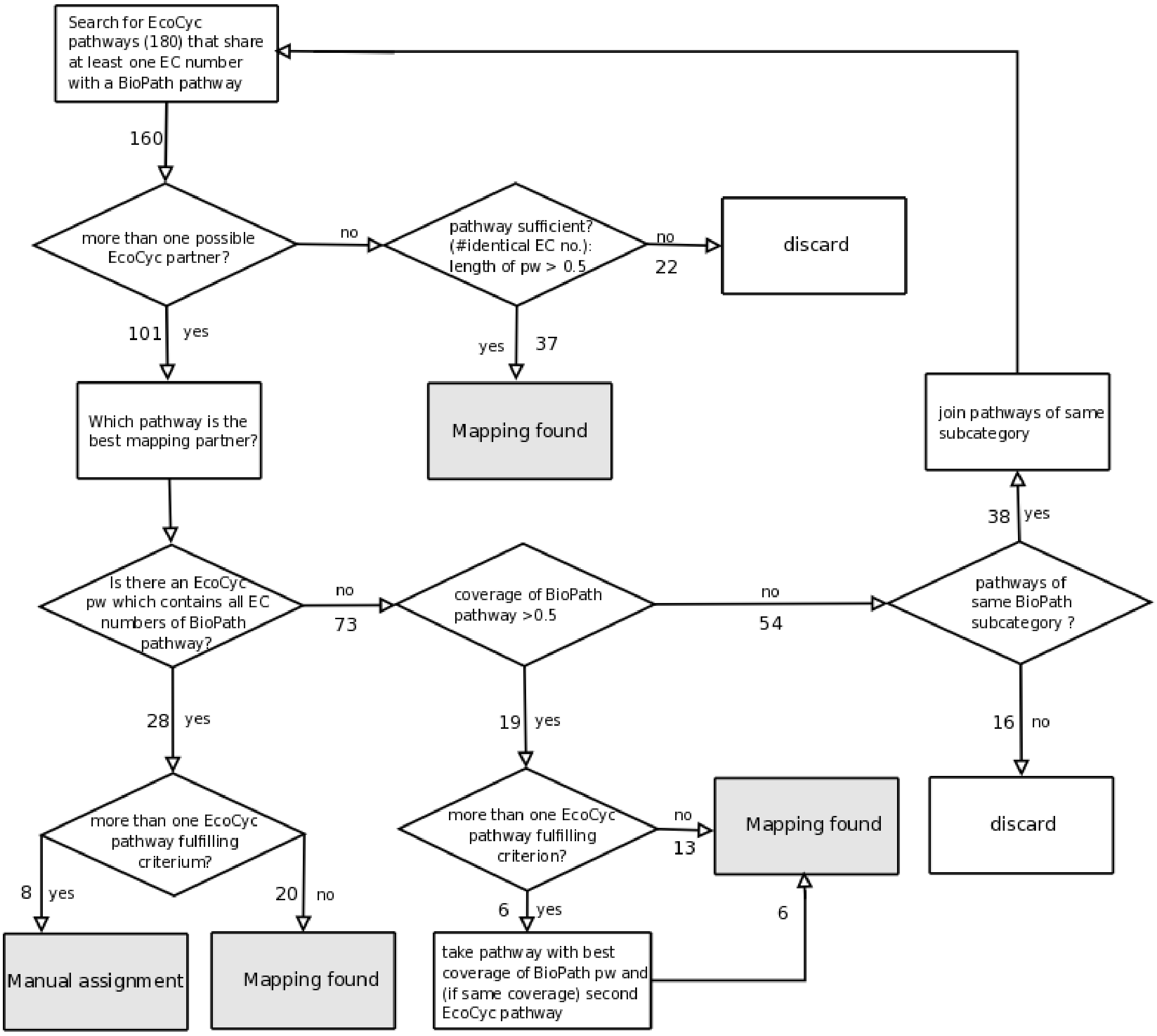

Figure S27: Automatic mapping of BioPath pathways onto EcoCyc pathways. The procedure described in the flow chart produces a list of BioPath pathways (or combinations of them) that are at least half-covered by an EcoCyc pathway.

mapped BioPath pathway should be predicted as present for *E.coli* by our reconstruction procedure since there is evidence for at least half of the enzymes according to EcoCyc. This has been the case for 100 (79%) out of 126 mappings. The complete mapping procedure is shown in Figure S27.

BioCyc pathway	mapped BioPath pathway(s) (score)	match of pred.	comment
chorismate biosynthesis	aaa1 (0.57)	y	
tryptophan biosynthesis	aaa6 (0.85)	y	
tyrosine biosynthesis I	aaa5 (0.60)	y	
glutamine biosynthesis I	glutamate7 (0.70)	y	
leucine biosynthesis	vas3 (1.00)	y	
valine biosynthesis	vas1 (0.52)	y	
arginine degradation III (arginine decarboxylase/agmatinase pathway)	urea4 (0.80)	y	
arginine biosynthesis II (acetyl cycle)	[urea1] (1.00), [urea2] (1.00), [urea5] (0.60)	y	
aspartate biosynthesis	[aspartatecl1] (1.00)	y	this MetaCyc pathway contains only one reaction; identical to 'aspartate degradation I'
aspartate degradation I	[aspartatecl1] (1.00)	y	(see above)
glycine biosynthesis I	[sg5] (0.50)	y	sg5 comprises both the biosynthesis and the cleavage
glycine cleavage complex	[sg5] (0.50)	y	(see above)
tRNA charging pathway	glutamate4 (1.00), arginine4 (1.00), methionine4 (1.00)	y	
homoserine biosynthesis	[threonine1] (1.00)	y	threonine1 comprises both homoserine and threonine biosynthesis
threonine biosynthesis from homoserine	[threonine1] (1.00)	y	(see above)
ornithine biosynthesis	urea5 (0.60)	y	
putrescine biosynthesis I	[polyamine4] (0.53)	y	polyamine4 describes the biosynthesis of spermine from L-arginine
spermidine biosynthesis	[polyamine4] (0.53)	y	(see above)
ribose degradation	[in1] (0.86)	y	this MetaCyc pathway contains only one reaction (= one reaction step in in1)
salvage pathways of pyrimidine ribonucleotides	[cn1] (0.59)	y	
PRPP biosynthesis I	[in1]/[un1] (0.86/0.80)	y	this MetaCyc pathway contains only one reaction (= one reaction step in in1 and un1)
pentose phosphate pathway (non-oxidative branch)	[ppc3] (1.00), [ppc4] (1.00)	y	
pentose phosphate pathway (partial)	ppc5 (1.00)	y	
methyl-coenzyme M reduction to methane	methane1 (0.67)	y	
tetrapyrrole biosynthesis I	[pyrrole1] (1.00), [pyrrole2] (0.92)	y	
glutamate dependent acid resistance	[glutamate6]	n	
isoleucine degradation I	vas5	n	
methionine degradation I (to homocysteine)	[methionine2]	n	
adenosylcobalamin biosynthesis II (late cobalt incorporation)	[coba1]	n	The corresponding pathway in BioPath lacks EC numbers for some of its reactions. Such reactions are treated as missing for the calculation of the pathway reconstruction score. Therefore, this pathway is not predicted when using the threshold.

Table S17: Pathways predicted for *M.jannaschii* by PathoLogic and mappable onto BioPath pathways. Pathways as provided by BioCyc are listed with the corresponding BioPath pathway(s) mapped. Mapped pathways are printed in brackets if the pathway definitions differ significantly. 'y' denotes pathways for which the mapped BioPath pathway is also predicted. 'n' denotes pathways for which the corresponding BioPath pathway(s) is/are not predicted.

BioCyc pathway	mapped BioPath pathway(s) (score)	match of pred.	comment
asparagine degradation I	?	?	contains only one reaction (= one out of five reactions of aspartate1 (score(p) = 0.4))
asparagine degradation II	?	?	is identical to 'asparagine degradation I'
canavanine biosynthesis	?	?	all reactions in this MetaCyc pathway for which enzymes have been identified are part of the urea cycle: urea1 and urea2 in BioPath
citrulline-nitric oxide cycle	?	?	no enzyme has been identified by PathoLogic for the only reaction which is different from (parts of) the urea cycle (corresponding to urea2 in BioPath); this unique reaction corresponds to arginine1 (score(p) = 1.0) in BioPath
respiration (anaerobic)–electron donors rct list	?	?	this "pathway" is a collection of single reactions; some of these reactions have been predicted by our reconstruction but are part of other pathways
respiration (anaerobic)	?	?	no such pathway in BioPath but all reactions are part of other pathways (e.g. gc1)
UDP-glucose conversion	?	?	no such pathway in BioPath but all reactions are part of other pathways (e.g. uronicacids1)
formate oxidation to CO2	?	?	corresponds to only one reaction out of the "pathway" above
glycolysis I	?	?	the glycolysis in BioPath is split into partial pathways (gg1, gg2, gg3, gg4, fructose4), each comprising several enzyme variants
TCA cycle	?	?	the TCA cycle is split into partial pathways (cc2,cc3,cc4,cc5) (see above)
polyisoprenoid biosynthesis	?	?	various BioPath pathways overlap: [isoprenoids11] (1.00), [isoprenoids1-9] (0.2-0.49)
asparagine biosynthesis I	-	-	contains only one reaction
2-keto glutarate dehydrogenase complex	-	-	
S-adenosyl-L-methionine cycle	-	-	
UDP-N-acetyl-D-glucosamine biosynthesis I	-	-	
selenocysteine biosynthesis	-	-	
biotin biosynthesis I	-	-	
biotin biosynthesis II	-	-	
biotin-carboxyl carrier protein	-	-	no such path in BioPath but two of three enzymatic reactions are part of other pathways (fa1,glf1)
branched-chain alpha-keto acid dehydrogenase complex	-	-	
factor 420 biosynthesis	-	-	
glycerol degradation II	-	-	
pyruvate dehydrogenase complex	-	-	
pyruvate fermentation to acetate III	-	-	
pyruvate fermentation to lactate	-	-	
reductive monocarboxylic acid cycle	-	-	
thioredoxin pathway	-	-	
adenosylcobalamin biosynthesis from cobyrinate a,c-diamide II	-	-	

Table S18: Pathways predicted for *M.jannaschii* by PathoLogic but not mappable onto BioPath pathways. '?' denotes pathways that are not mappable onto BioPath pathways due to differences in pathway definitions. '-' denotes pathways lacked by BioPath.
